# Reaction of Papaverine with Baran Diversinates^TM^

**DOI:** 10.3390/molecules24213938

**Published:** 2019-10-31

**Authors:** Folake A. Egbewande, Mark J. Coster, Ian D. Jenkins, Rohan A. Davis

**Affiliations:** Griffith Institute for Drug Discovery, Griffith University, Brisbane, QLD 4111, Australia; f.egbewande@griffith.edu.au (F.A.E.); m.coster@griffith.edu.au (M.J.C.); i.jenkins@griffith.edu.au (I.D.J.)

**Keywords:** late-stage functionalization, sulfinate, Diversinate^TM^, natural product, medicinal chemistry, papaverine, scaffold, library, biodiscovery

## Abstract

The reaction of papaverine with a series of Baran Diversinates^TM^ is reported. Although the yields were low, it was possible to synthesize a small biodiscovery library using this plant alkaloid as a scaffold for late-stage C–H functionalization. Ten papaverine analogues (**2**–**11**), including seven new compounds, were synthesized. An unexpected radical-induced exchange reaction is reported where the dimethoxybenzyl group of papaverine was replaced by an alkyl group. This side reaction enabled the synthesis of additional novel fragments based on the isoquinoline scaffold, which is present in numerous natural products. Possible reasons for the poor yields in the Diversinate^TM^ reactions with this particular scaffold are discussed.

## 1. Introduction

Late-stage functionalization of organic molecules has emerged as an important strategy in modern drug discovery programs as it allows for direct derivatization without the need for pre-functionalized synthetic handles [1]. This strategy, which involves the direct substitution of a C–H bond with a new functionality, is currently under-utilized in the field of natural products where the generation of analogues provides structure–activity relationships (SAR), a critical component often missing in current biodiscovery programs. The ability to make unusual derivatives of a bioactive scaffold via a simple one-step procedure is of particular relevance in the field of natural products where typically, only small amounts of material or analogues are available. Nitrogen-rich heterocyclic compounds sourced from nature have played a profound role in human health and these motifs are found in many of the current drugs that are used to treat various diseases [2]. Transition-metal-mediated cross-coupling reactions that require pre-functionalized starting materials have been used extensively in the synthesis of such molecules [3,4,5]; however, the direct C–H functionalization of biologically active heterocycles is still underdeveloped and worthy of further investigations [6,7,8,9,10,11,12,13,14].

Recent developments in radical-mediated C–H functionalization of heterocycles, including Minisci [15], borono-Minisci [7], and reactions with sulfinate reagents [2], have led to a resurgence in the use of radical-based methods, due primarily to improvements in substrate scope and mild reaction conditions.

In 1971, Minisci et al. reported the addition of carbon-centered radicals to heteroaromatic bases through the silver-mediated decarboxylation of carboxylic acids in the presence of persulfate [15]. One feature that makes this form of innate C–H functionalization appealing for pharmaceutical applications is that protection and deprotection protocols are rarely needed [6]. While these conditions are compatible with alkyl and acyl radicals (derived from alkyl halides, carboxylic acids, and related derivatives), limitations in functional group compatibility, high reaction temperatures (>70 °C), and the requirement of transition-metal additives and strongly oxidizing conditions [6] make them unsuitable for more complex chemical structures. Since this initial publication, numerous methods of C–H functionalization have been reported by Baran [2,7,9,14], Molander [8], and others [16,17], which have significantly increased the scope and generality of this strategy. With the goal of the direct transformation of C–H bonds into C–C bonds in a more practical manner, Baran et al. have developed a radical-based functionalization strategy that involves the use of zinc bis(alkanesulfinate) reagents [12,14]. The most notable features of C–C bond formation using this sulfinate chemistry are that it involves a one-pot reaction, occurs under mild conditions with no need for pre-functionalized starting materials, and the reactions can be conducted in open flasks as they do not require the exclusion of air or moisture [2]. This approach has the advantage of rapidly accelerating drug discovery timelines regardless of whether the compounds of interest are natural or synthetic.

With the intent of identifying hit or lead compounds that are based on bioactive natural products, our research focuses on the semi-synthesis of biodiscovery libraries using unique natural product scaffolds that have been isolated from sources such as fungi, plants and marine invertebrates [18,19,20,21,22,23,24]. Herein, we report late-stage functionalization studies on papaverine, a nitrogen-containing heterocyclic natural product, using the commercially available sulfinate reagents known as Diversinates^TM^. Some unexpected chemistry was identified during these studies.

## 2. Results and Discussion

Commercially available papaverine hydrochloride (**1a**) [25] was chosen as a model compound for our initial foray into C–H functionalization studies utilizing the sulfinate chemistry that has been described by Baran and other research groups [2,9,14]. Fluoroalkyl substituents have become increasingly valuable in modern drug discovery due to their resistance toward oxidation by cytochrome P450 oxidases. Also, incorporation of halogen atoms on hit/lead compounds has been performed in order to exploit their steric effects through the ability of these atoms to occupy the active site of molecular targets [26,27,28], and establish intermolecular bonds in a manner that resembles H-bonding [29,30,31].

Baran et al. have published a number of sulfinate reaction conditions, where the Diversinate^TM^ reagents are compatible with different organic solvents (e.g., DMSO, CH_2_Cl_2_, ClCH_2_CH_2_Cl, perfluorotoluene, perfluorohexane, and anisole). Furthermore, it has been determined that fluoroalkyl zinc sulfinate reagents react best in halogenated solvents, such as CH_2_Cl_2_, alkyl zinc sulfinate salts react more favorably in DMSO [2], and stoichiometric conditions for the peroxide and Diversinate^TM^ reagents, as well as reaction temperatures and times, vary greatly in the literature. Before synthesizing the targeted papaverine library, we initially conducted several experiments that tested the effect of solvents (e.g., DMSO/CH_2_Cl_2_^/^H_2_O), reagent stoichiometry, and additives [e.g., trifluoroacetic acid (TFA)] with papaverine HCl (**1a**) and the commonly used Diversinate^TM^, zinc trifluoromethanesulfinate [(CF_3_SO_2_)_2_Zn] (Table S71). From this data, it was clear that the best yield for the major mono-CF_3_ analogue (**2**) was obtained using 6 mol eq. of (CF_3_SO_2_)_2_Zn and 6 mol eq. of *tert*-butyl hydroperoxide (TBHP) in CH_2_Cl_2_ for 16 h. In order to ascertain whether the presence of HCl was affecting the yields (the chloride ion could be competitively oxidized by TBHP), we generated the free base of papaverine (**1b**) and repeated the test reactions. Surprisingly, the reaction on the free base gave a lower yield of **2** (15%) compared to the papaverine HCl reaction (compound **2**, 24%). Subsequently, the effect of the addition of TFA was investigated using both the free base and HCl salt of papaverine and a mixture of products was produced, with the free base (**1b**) affording the best yield (10%) with the solvent conditions CH_2_Cl_2_/TFA/H_2_O (1 mol eq. TFA). Based on these data (Table S71), all subsequent reactions were performed using the HCl salt of papaverine and 6 mol eq. of the Diversinate^TM^ reagent in CH_2_Cl_2_/H_2_O (2.5:1) for the fluorinated Diversinates^TM^, while DMSO/H_2_O (2.5:1) was chosen for use with the non-fluorinated Diversinates^TM^.

**Scheme 1:**
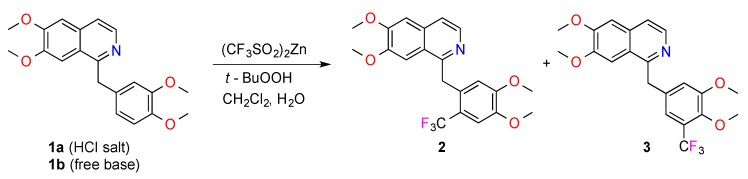
Reaction of papaverine HCl (**1a**) and free base (**1b**) with zinc trifluoromethanesulfinate [(CF_3_SO_2_)_2_Zn] and *tert*-butyl hydroperoxide (TBHP) in CH_2_Cl_2_/H_2_O.

For example, scaffold **1a** (0.1 mmol of the HCl salt) was treated with (CF_3_SO_2_)_2_Zn (0.6 mmol) in a mixture of CH_2_Cl_2_ (100 μL) and H_2_O (40 μL) at 0 °C. The mixture was stirred at 0 °C and TBHP (0.6 mmol) was slowly added, followed by stirring for 20 min. The mixture was then allowed to warm to room temperature over 16 h [2], before evaporation under a stream of nitrogen and chromatography (HPLC on NH_2_-bonded silica) was undertaken in order to give the major products, substituted papaverines **2** (24%) and **3** (4%), and the recovered starting material (7%) (Scheme 1). HPLC showed a multitude of UV-active peaks. This, and the recovery of only 7% of the starting material, suggested that the reaction of trifluoromethyl radicals with papaverine was very unselective and/or the products were unstable under the reaction conditions. The preferred attack on the electron-rich 3,4-dimethoxybenzyl ring was not surprising as the trifluoromethyl radical is electrophilic.

**Figure 1:**
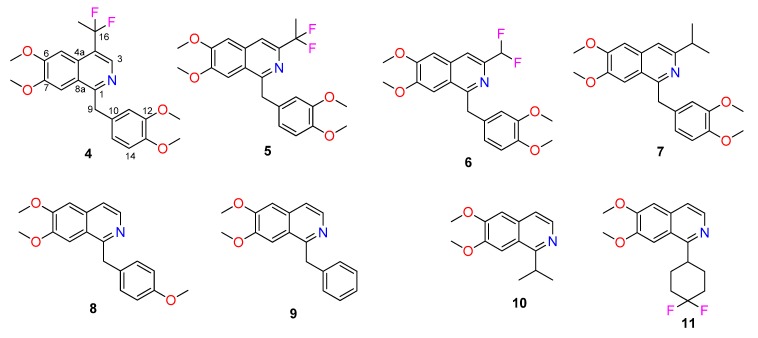
Chemical structures of the other synthesized papaverine analogues **4**–**11**.

Interestingly, when the reaction was repeated with the less electrophilic reagent sodium 1,1-difluoroethanesulfinate, the products obtained were those involving substitution on the isoquinoline ring, rather than substitution on the dimethoxybenzyl ring. The products obtained were **4** (7%) and **5** (3%), as well as the recovered starting material (8%) (Figure 1). In all of these reactions, a significant amount (generally between 7 and 14%) of starting material was recovered even though a six-fold excess of reagents was employed.

The closely related zinc difluoromethanesulfinate gave a low yield of **6** (3%) analogous to **5**, but only when the reaction was carried out in the presence of TFA (0.1 mmol). No products were detected in the absence of TFA. Although the reaction employed the HCl salt of papaverine under the reaction conditions of excess (CF_2_HSO_2_)_2_Zn and *t*-BuOOH, the HCl would be neutralized by the zinc hydroxide formed. Presumably, the addition of TFA ensured at least partial protonation of the isoquinoline nitrogen, rendering the papaverine more electrophilic and therefore more reactive toward radicals that had some nucleophilic character. Baran et al. found that the difluoromethyl radical had nucleophilic properties, preferring to attack *N*-heterocyclic compounds at electron-deficient centers [12].

**Scheme 2:**
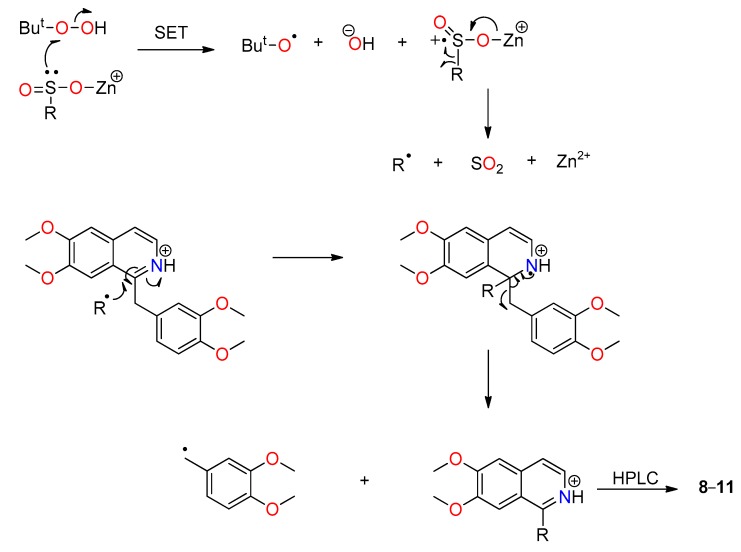
Proposed single-electron transfer (SET) mechanism for the formation of the side-products **8**–**11**.

Use of the nucleophilic radical precursor zinc isopropylsulfinate gave the papaverine derivative **7** (4%), analogous to **5** and **6**. Once again, TFA was required for the formation of **7**. Surprisingly, the major product was **10** (19%), formed by an apparent radical substitution reaction. Analogous reactions were observed with the nucleophilic radical precursors zinc 4-methoxybenzylsulfinate, zinc benzylsulfinate, and 4,4-difluorocyclohexylsulfinate to give **8** (4%), **9** (14%), and **11** (4%), respectively. A suggested mechanism for these radical substitution reactions is outlined in Scheme 2.

The isolated yields in these reactions were generally low. This was partly due to the large number of minor products formed with this particular scaffold (as evidenced by the HPLC data) and partly due to losses during HPLC purification. Some of these minor products may be due to the attack by trifluoromethyl radicals on the dimethoxyisoquinoline ring and/or bis-trifluoromethylation (which could yield 21 different compounds). In addition, the papaverine scaffold contains four methoxyl groups and a benzylic methylene group, all of which could undergo hydrogen atom abstraction by *tert*-butoxyl radicals leading to a plethora of minor products. Interestingly, Kuttruff et al. [32] have recently reported rather low yields (3–30%) and sometimes extensive decomposition with these reactions. They employed a range of scaffolds incorporating benzene, pyridine, pyrimidine, imidazole, pyrazole and thiazole rings. They also explored different reaction conditions and were able to achieve modest increases in yields in some cases via the addition of Fe(acac)_3_. However, in other cases, the presence of Fe(acac)_3_ led to rapid decomposition. Baran et al. [9] have also noted that one of the limitations of the method is that some substrates deliver only moderate amounts of product.

We suggest that the main reason for the poor reactivity of **1a** toward difluoromethyl radicals is the presence of the dimethoxybenzyl group at position 1. Difluoromethyl radicals react readily with isoquinoline and with 3-methylisoquinoline at position 1 (the most electrophilic position), but they do not react with 1-methylisoquinoline [33]. It is interesting to note that the more nucleophilic isopropyl radicals attack at position 1 of the papaverine despite the significant steric hindrance to attack at this position. This is followed by loss of the (more stable) 3,4-dimethoxybenzyl radical to give **10** in moderate (19%) yield.

Compounds **2**, **8**, and **9** have previously been synthesized via multi-step syntheses; **2** was generated using the copper-mediated trifluoromethylation-allylation of arynes [34]; **8** was produced from a reaction of Raney nickel with N-benzylsulfonamide [35], and via the desulfonylation of N-sulfonyl tetrahydroisoquinolines with KF/Al_2_O_3_ [36]; and **9** was synthesized using a ruthenium-mediated dual catalytic reaction, and oxidative cross-dehydrogenative coupling with methyl arenes [37,38]. However, this is the first report of the synthesis of **2**, **8**, and **9** using sulfinate chemistry. Furthermore, these three compounds were only partially characterized and none of them had their ^1^H and ^13^C chemical shifts assigned to specific positions within the alkaloidal skeleton. We report here the first synthesis of several other papaverine analogues as their free base; full characterization using 1D/2D NMR, UV, IR, and MS data was performed during these studies.

**Figure 2:**
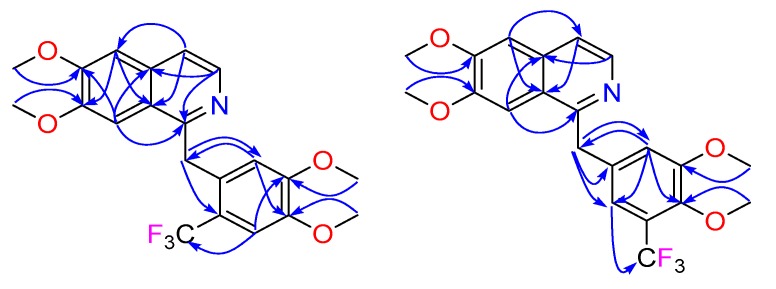
Key HMBC (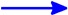) correlations for **2** and **3**.

The first products to be fully characterized were compounds **2** and **3**. The ^1^H NMR spectra (Table 1) of these two mono-CF_3_ derivatives enabled the definitive positioning of the CF_3._ Careful comparison of the natural product scaffold (**1b**) NMR data in CD_3_OD with the fluorinated analogue **2** indicated that this molecule had a CF_3_ group attached to C-15, since the ^1^H NMR chemical shifts and multiplicities associated with the pendant benzyl moiety of the starting material had changed from a classic 1,3,4-trisubstituted benzene system (δ_H_ 7.03, d, *J* = 2.1 Hz, H-11, 6.92, d, *J* = 8.4 Hz, H-14 and 6.82, dd, *J* = 8.4, 2.1 Hz, H-15) to a 1,3,4,6-tetrasubstituted benzene system (δ_H_ 6.41, s, H-11 and 7.26, s, H-14) (Table 1). Furthermore, a heteronuclear multiple bond correlation (HMBC) (Figure 2) from H-14 (*δ*_H_ 7.26) to CF_3_ (*δ*_C_ 126.4) provided further proof of the structural assignment. In a similar fashion to **2**, NMR data analysis of **3** also showed that the CF_3_ was attached to the pendant aromatic ring of papaverine; however, in this case, the fluorinated moiety was attached to C-14, as indicated once again by the ^1^H NMR chemical shifts and multiplicities associated with the pendant benzyl moiety of papaverine that had changed from the 1,3,4-trisubstituted benzene system to a 1,3,4,5-tetrasubstituted benzene system (δ_H_ 7.22, d, *J* = 2.2 Hz, H-11 and 7.11, d, *J* = 2.2 Hz, H-15) (Table 1). The HMBC spectrum analysis of **3** was also critical in confirming the CF_3_ positioning; key HMBC correlations for **3** are shown in Figure 2.

Detailed 2D NMR data analyses were also performed on all other analogues generated during these studies (see Supplementary Materials).

Surprisingly, the reactions that used other commercially available Diversinates^TM^, such as zinc chloromethanesulfinate, zinc chloroethanesulfinate, sodium (2,4-dichlorophenyl)methanesulfinate, sodium 1-(trifluoromethyl)cyclopropanesulfinate, and sodium *tert*-butylsulfinate were not successful using our methodology (with or without TFA), as determined by LCMS analysis of the crude reaction mixtures after 16 h. The lack of reaction with sodium *tert*-butylsulfinate and sodium 1-(trifluoromethyl)cyclopropanesulfinate was possibly due to steric hindrance, but it is unclear why the other Diversinate^TM^ reactions were unsuccessful.

In Scheme 2, we propose that the zinc bis(alkanesulfinate) underwent oxidation by TBHP via a single-electron transfer (SET) process to give a *tert*-butoxyl radical, hydroxide ion, and the radical cation of the zinc sulfinate, which then underwent fragmentation to give the alkyl radical and sulfur dioxide. The nucleophilic alkyl radical then underwent addition at position 1 of papaverine followed by elimination of the dimethoxybenzyl radical. This addition–elimination mechanism is analogous to that proposed for the reaction of carbon-centered radicals with β-bromostyrene [39]. The *tert*-butoxyl radical generated in the first step could oxidize a second molecule of sulfinate to give the *tert*-butoxide anion and the radical cation of the zinc sulfinate. A similar mechanism has been proposed by Baran et al. [9] where the presence of a trace metal initiates *tert*-butoxyl radical formation, compared to our proposed mechanism that involves SET. The dimethoxybenzyl radical could also be oxidized by TBHP to give the corresponding dimethoxybenzyl alcohol and a *tert*-butoxyl radical.

Again, it is interesting that the more nucleophilic radicals attack position 1 of the isoquinoline ring, while the 1,1-difluoroethyl and difluoromethyl radicals, which appear to have both nucleophilic and electrophilic properties, prefer to attack at the 3- or 4-position.

**Scheme 3:**
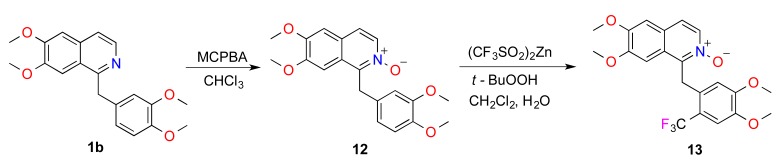
Conversion of papaverine free base (**1b**) to papaverine *N*-oxide (**12**) using *meta*-chloroperbenzoic acid (MCPBA) in CHCl_3_ and the subsequent reaction of **12** with zinc trifluoromethanesulfinate [(CF_3_SO_2_)_2_Zn] and *tert*-butyl hydroperoxide (TBHP) in CH_2_Cl_2_/H_2_O to form compound **13**.

In a final attempt to improve the yields and/or the selectivity of these reactions, we decided to modify the reactivity of the papaverine scaffold. It was envisaged that converting papaverine to its *N*-oxide might increase reactivity, particularly toward electrophilic trifluoromethyl radicals. The free base of papaverine was readily converted to the corresponding *N*-oxide derivative **12** in moderate yield (58%) using *meta*-chloroperbenzoic acid (MCPBA) in CHCl_3_ without the need for chromatography, using a method previously described by Bremner et al. [40] (Scheme 3). Unfortunately, treatment of **12** with (CF_3_SO_2_)_2_Zn and TBHP under the same conditions as that used for **1a** did not result in an improvement in the yield of the desired Diversinate^TM^ product. After work-up and C_18_ HPLC purification of the reaction mixture, ^1^H NMR and LCMS analysis indicated that the major product formed was compound **13,** albeit with a low yield (<12%) and purity (<80%). Significant amounts of starting material (**12**, 26%) were isolated, indicating that papaverine *N*-oxide was less reactive than papaverine toward zinc trifluoromethanesulfinate. As the yield of the desired product had not improved, the reaction of papaverine *N*-oxide with other Diversinate^TM^ reagents was not investigated.

## 3. Materials and Methods

### 3.1. General Experimental Procedures

NMR spectra were recorded at 25 °C on a Bruker AVANCE HDX 800 MHz spectrometer (Fällanden, Zürich, Switzerland) equipped with a TCI cryoprobe. The ^1^H and ^13^C NMR chemical shifts were referenced to the solvent peak for CD_3_OD (δ_H_ 3.31 and δ_C_ 49.00) and CDCl_3_ (δ_H_ 7.26 and δ_C_ 77.16). UV spectra were recorded using a JASCO V-650 UV/vis spectrophotometer (Tokyo, Japan). IR data were acquired using an attached Universal Attenuated Total Reflectance (UATR) Two module on a PerkinElmer spectrophotometer (Waltham, MA, USA). LRESIMS data were recorded on a Thermo Fisher MSQ Plus single quadrupole mass spectrometer (Waltham, MA, USA). HRESIMS data were recorded on a Bruker maXis II ETD ESI-qTOF (Bremen, Germany). A Thermo Scientific Dionex Ultimate 3000 HPLC (Waltham, MA, USA) was used for semi-preparative HPLC separations. An Alltech stainless steel guard cartridge (10 mm × 30 mm) (Sydney, NSW, Australia) was used for loading pre-adsorbed synthetic reaction products onto the semi-preparative HPLC columns. Alltech Davisil NH_2_-bonded silica (35–75 µm, 150 Å) (Sydney, NSW, Australia) and C_18_-bonded silica (35–75 µm, 150 Å) (Sydney, NSW, Australia) were used for pre-adsorption work before HPLC separations. TLC was carried out on Merck gel F_254s_ pre-coated NH_2_ glass plates (Darmstadt, Germany) and was observed using UV light. A YMC NH_2_-bonded silica column (5 µm, 120 Å, 20 mm × 150 mm) (Kyoto, Japan) and a ThermoElectron Betasil C_18_-bonded silica column (5 µm, 143 Å, 21.2 × 150 mm) (Waltham, MA, USA) were used for semi-preparative HPLC separations. All solvents used for chromatography, UV and MS were Honeywell Burdick & Jackson HPLC grade (Muskegon, MI, USA), and the H_2_O was Sartorius arium proVF (Göttingen, Germany) filtered. All synthetic reagents (including papaverine HCl) were purchased from Sigma-Aldrich (St. Louis, MO, USA) and used without further purification. NMR spectra were processed using MestReNova version 11.0 (Santiago de Compostela, Spain).

### 3.2. Generation of the Papaverine Library (TFA-Free Method): Compounds **2**–**5**, **9**, and **11**

Papaverine hydrochloride (**1a**, 37.5 mg, 0.1 mmol) was dissolved in CH_2_Cl_2_/H_2_O or DMSO/H_2_O (2.5:1, 100 μL:40 μL) before the addition of the sulfinate reagent (85.6–198.9 mg, 0.6 mmol) at room temperature. The mixture was cooled to 0 °C before TBHP (40 μL, 0.6 mmol) was slowly added, after which time the stirred mixture slowly warmed to room temperature over 16 h. Crude reaction products were dried under N_2_ and pre-adsorbed to NH_2_-bonded silica (≈1 g) overnight with the dry material packed into a stainless steel guard cartridge, which was subsequently attached to a NH_2_ semi-preparative HPLC column. Isocratic conditions of 100% *n*-hexane for 10 min, followed by a linear gradient to 20% *i*-PrOH/*n*-hexane over 50 min, then isocratic conditions of 20% *i*-PrOH/*n*-hexane for 10 min all at a flow rate of 9 mL/min was used for each HPLC separation. Sixty fractions (60 × 1 min) were collected from the start of the HPLC run. Fractions containing UV-active material from each HPLC run were analyzed using ^1^H NMR spectroscopy and LCMS, and relevant fractions with a purity >90% were combined to produce the desired products. Compounds **2**–**5** and **11** were synthesized using the CH_2_Cl_2_/H_2_O solvent system, while compound **9** was generated using the DMSO/H_2_O mixture.

#### 3.2.1. Compound **2**

Colorless gum (6.0 mg, 15%); UV (MeOH) λ_max_ (log ε) 241 (4.20), 279 (3.30), 327 (3.19) nm; IR (UATR) ν_max_ 3440, 2981, 1747, 1709, 1279, 1199, 1167, 1078, 1026, 976, 712 cm^−1^; For NMR data see Table 1; (+)-LRESIMS *m*/*z* (rel. int.) 408 (100) [M + H]^+^; (+)-HRESIMS *m*/*z* 408.1425 [M + H]^+^ (calcd for C_21_H_21_F_3_NO_4_, 408.1417).

#### 3.2.2. Compound **3**

Colorless gum (1.9 mg, 4%); UV (MeOH) λ_max_ (log ε) 240 (4.24), 283 (3.47), 324 (3.20) nm; IR (UATR) ν_max_ 3440, 2982, 1746, 1709, 1278, 1199, 1168, 1078, 1026, 975, 712 cm^−1^; For NMR data see Table 1; (+)-LRESIMS *m*/*z* (rel. int.) 408 (100) [M + H]^+^; (+)-HRESIMS *m*/*z* 408.1422 [M + H]^+^ (calcd for C_21_H_21_F_3_NO_4_, 408.1417).

#### 3.2.3. Compound **4**

Colorless gum (2.7 mg, 7%); UV (MeOH) λ_max_ (log ε) 240 (4.08), 276 (3.35), 327 (3.17) nm; IR (UATR) ν_max_ 3400, 2981, 1747, 1705, 1278, 1200, 1164, 1078, 975, 712 cm^−1^; ^1^H NMR (800 MHz, CD_3_OD) δ_H_ 2.17 (3H, t, *J* = 18.8 Hz, H-17), 3.74 (3H, s, 12-OMe), 3.75 (3H, s, 13-OMe), 3.87 (3H, s, 7-OMe), 3.97 (3H, s, 6-OMe), 4.56 (2H, s, H-9), 6.77 (1H, dd, *J* = 8.2, 2.0 Hz, H-15), 6.83 (1H, d, *J* = 8.2 Hz, H-14), 6.92 (1H, d, *J* = 2.0 Hz, H-11), 7.51 (1H, s, H-5), 7.53 (1H, s, H-8), 8.42 (1H, s, H-3); ^13^C NMR (200 MHz, CD_3_OD) δ_C_ 25.5 (t, ^2^*J*_CF_ = 28.6 Hz, C-17), 42.4 (C-9), 56.36 (12-OMe), 56.38 (13-OMe), 56.41 (7-OMe), 56.5 (6-OMe), 104.4 (C-5), 106.4 (C-8), 113.2 (C-14), 113.6 (C-11), 122.0 (C-15), 124.0 (t, ^1^*J*_CF_ = 238.3 Hz, C-16), 124.6 (C-8a), 126.8 (t, ^2^*J*_CF_ = 25.3 Hz, C-4), 131.2 (C-4a), 133.1 (C-10), 138.1 (t, ^3^*J*_CF_ = 9.3 Hz, C-3), 149.3 (C-13), 150.6 (C-12), 151.6 (C-7), 154.8 (C-6), 162.1 (C-1); (+)-LRESIMS *m*/*z* (rel. int.) 404 (100) [M + H]^+^; (+)-HRESIMS *m*/*z* 426.1481 [M + Na]^+^ (calcd for C_22_H_23_F_2_NO_4_Na, 426.1487).

#### 3.2.4. Compound **5**

Colorless gum (1.3 mg, 3%); UV (MeOH) λ_max_ (log ε) 243 (3.95), 283 (3.19), 328 (3.08) nm; IR (UATR) ν_max_ 3400, 2972, 1747, 1705, 1279, 1199, 1165, 1078, 976, 712 cm^−1^; ^1^H NMR (800 MHz, CD_3_OD) δ_H_ 2.08 (3H, t, *J* = 18.4 Hz, H-17), 3.73 (3H, s, 12-OMe), 3.75 (3H, s, 13-OMe), 3.88 (3H, s, 7-OMe), 3.97 (3H, s, 6-OMe), 4.56 (2H, s, H-9), 6.80 (1H, dd, *J* = 8.3, 2.0 Hz, H-15), 6.83 (1H, d, *J* = 8.3 Hz, H-14), 6.95 (1H, d, *J* = 2.0 Hz, H-11), 7.34 (1H, s, H-5), 7.46 (1H, s, H-8), 7.86 (1H, s, H-4); ^13^C NMR (200 MHz, CD_3_OD) δ_C_ 24.4 (t, ^2^*J*_CF_ = 28.7 Hz, C-17), 41.9 (C-9), 56.3 (12-OMe), 56.39 (13-OMe), 56.41 (7-OMe), 56.5 (6-OMe), 107.4 (C-5), 105.5 (C-8), 113.0 (C-14), 113.4 (C-11), 115.7 (t, ^3^*J*_CF_ = 5.3 Hz, C-4), 121.8 (C-15), 122.5 (t, ^1^*J*_CF_ = 237.4 Hz, C-16), 124.3 (C-8a), 133.5 (C-10), 135.0 (C-4a), 147.6 (t, ^2^*J*_CF_ = 28.3 Hz, C-3), 149.0 (C-13), 150.4 (C-12), 152.3 (C-7), 154.7 (C-6), 159.8 (C-1); (+)-LRESIMS *m*/*z* (rel. int.) 404 (100) [M + H]^+^; (+)-HRESIMS *m*/*z* 426.1478 [M + Na]^+^ (calcd for C_22_H_23_F_2_NO_4_Na, 426.1487).

#### 3.2.5. Compound **9**

Colorless gum (4.0 mg, 14%); UV (MeOH) λ_max_ (log ε) 241 (3.90), 326 (2.94) nm; IR (UATR) ν_max_ 3458, 2981, 1747, 1705, 1278, 1199, 1165, 1078, 1026, 975, 712 cm^−1^; ^1^H NMR (800 MHz, CD_3_OD) δ_H_ 3.83 (3H, s, 7-OMe), 3.97 (3H, s, 6-OMe), 4.60 (2H, s, H-9), 7.16 (1H, m, H-13), 7.24 (2H, m, H-11, H-15), 7.27 (1H, s, H-5), 7.38 (1H, s, H-8), 7.40 (2H, m, H-12, H-14), 7.61 (1H, d, *J* = 5.7 Hz, H-4), 8.22 (1H, d, *J* = 5.7 Hz, H-3); ^13^C NMR (200 MHz, CD_3_OD) δ_C_ 42.5 (C-9), 56.3 (7-OMe), 56.5 (6-OMe), 105.5 (C-8), 106.5 (C-5), 120.6 (C-4), 124.3 (C-8a), 127.4 (C-13), 129.6 (2C, C-11, C-15), 129.5 (2C, C-12, C-14), 135.5 (C-4a), 140.5 (C-10), 140.8 (C-3), 151.7 (C-7), 154.6 (C-6), 158.9 (C-1); (+)-LRESIMS *m*/*z* (rel. int.) 280 (100) [M + H]^+^; (+)-HRESIMS *m*/*z* 280.1327 [M + H]^+^ (calcd for C_18_H_18_NO_2_, 280.1332).

#### 3.2.6. Compound **11**

Colorless gum (1.2 mg, 4%); UV (MeOH) λ_max_ (log ε) 237 (3.94), 327 (2.96) nm; IR (UATR) ν_max_ 3436, 2986, 1747, 1705, 1280, 1199, 1166, 1078, 1026, 975, 712 cm^−^^1^; ^1^H NMR (800 MHz, CD_3_OD) δ_H_ 2.00–2.11 (4H, m, H-10, H-14), 2.12–2.21 (4H, m, H-11, H-13), 3.71 (1H, m, H-9), 3.99 (3H, s, 6-OMe), 4.03 (3H, s, 7-OMe), 7.28 (1H, s, H-5), 7.52 (1H, d, *J* = 5.7 Hz, H-4), 7.54 (1H, s, H-8), 8.19 (1H, d, *J* = 5.7 Hz, H-3); ^13^C NMR (200 MHz, CD_3_OD) δ_C_ 29.6 (2C, d, ^3^*J*_CF_ = 10.3 Hz, C-10, C-14), 34.8 (2C, dd, ^2^*J*_CF_ = 25.5, 22.6 Hz, C-11, C-13), 40.2 (C-9), 56.46 (6-OMe), 56.54 (7-OMe), 104.2 (C-8), 106.9 (C-5), 119.9 (C-4), 123.3 (C-8a), 124.5 (dd, ^1^*J*_CF_ = 242.0, 239.2 Hz, C-12), 135.2 (C-4a), 140.9 (C-3), 152.0 (C-7), 154.5 (C-6), 162.3 (C-1); (+)-LRESIMS *m*/*z* (rel. int.) 308 (100) [M + H]^+^; (+)-HRESIMS *m*/*z* 308.1454 [M + H]^+^ (calcd for C_17_H_20_F_2_NO_2_, 308.1457).

### 3.3. Generation of the Papaverine Library (TFA-Addition Method): Compounds **6**–**8** and **10**

Papaverine hydrochloride (**1a**, 37.5 mg, 0.1 mmol) was dissolved in CH_2_Cl_2_/H_2_O or DMSO/H_2_O (2.5:1, 100 μL:40 μL) before the addition of the sulfinate reagent (85.6–198.9 mg, 0.6 mmol) and TFA (7.7 μL, 0.1 mmol) at room temperature. The mixture was cooled to 0 °C before TBHP (40 μL, 0.6 mmol) was slowly added, after which time, the stirred mixture slowly warmed to room temperature over 16 h. Crude reaction products was dried under N_2_ and pre-adsorbed to NH_2_-bonded silica (≈1 g) overnight before being subjected to identical NH_2_ semi-preparative HPLC conditions, which are described above. This method generated compound **6** using the CH_2_Cl_2_/H_2_O solvent system, while compounds **7**, **8**, and **10** were generated using the DMSO/H_2_O mixture.

#### 3.3.1. Compound **6**

Colorless gum (1.0 mg, 3%); UV (MeOH) λ_max_ (log ε) 240 (4.15), 273 (3.36), 327 (3.08) nm; IR (UATR) ν_max_ 3458, 2981, 1747, 1705, 1278, 1199, 1165, 1078, 1026, 975, 712 cm^−1^; ^1^H NMR (800 MHz, CD_3_OD) *δ*_H_ 3.72 (3H, s, 12-OMe), 3.76 (3H, s, 13-OMe), 3.87 (3H, s, 7-OMe), 3.98 (3H, s, 6-OMe), 4.57 (2H, s, H-9), 6.78 (1H, dd, *J* = 8.3, 2.0 Hz, H-15), 6.83 (1H, d, *J* = 8.3 Hz, H-14), 6.84 (1H, t, *J* = 55.7 Hz, H-16), 6.93 (1H, d, *J* = 2.0 Hz, H-11), 7.37 (1H, s, H-5), 7.48 (1H, s, H-8), 7.88 (1H, s, H-4); ^13^C NMR (200 MHz, CD_3_OD) *δ*_C_ 42.0 (C-9), 56.4 (12-OMe), 56.45 (13-OMe), 56.49 (7-OMe), 56.6 (6-OMe), 107.4 (C-5), 105.8 (C-8), 113.2 (C-14), 113.6 (C-11), 115.6 (t, ^1^*J*_CF_ = 238.0 Hz, C-16), 117.4 (t, ^3^*J*_CF_ = 4.8 Hz, C-4), 121.9 (C-15), 125.0 (C-8a), 133.4 (C-10), 135.0 (C-4a), 144.9 (t, ^2^*J*_CF_ = 24.3 Hz, C-3), 149.2 (C-13), 150.6 (C-12), 152.6 (C-7), 154.9 (C-6), 160.1 (C-1); (+)-LRESIMS *m*/*z* (rel. int.) 390 (100) [M + H]^+^; (+)-HRESIMS *m*/*z* 390.1506 [M + H]^+^ (calcd for C_21_H_22_F_2_NO_4_, 390.1511).

#### 3.3.2. Compound **7**

Colorless gum (1.5 mg, 4%); UV (MeOH) λ_max_ (log ε) 239 (3.72), 276 (3.00), 328 (2.83) nm; IR (UATR) ν_max_ 3413, 2986, 1756, 1718, 1279, 1198, 1170, 1078, 1025, 973, 712 cm^−1^; δ_H_ 1.40 (6H, d, *J* = 6.9 Hz, H-17), 3.22 (1H, sept, *J* = 6.9 Hz, H-16), 3.72 (3H, s, 12-OMe), 3.76 (3H, s, 13-OMe), 3.84 (1H, s, 7-OMe), 3.96 (3H, s, 6-OMe), 4.53 (1H, s, H-9), 6.78 (1H, dd, *J* = 8.5, 2.2 Hz, H-15), 6.83 (1H, d, *J* = 8.5 Hz, H-14), 6.91 (1H, d, *J* = 2.2 Hz, H-11), 7.21 (1H, s, H-5), 7.44 (1H, s, H-4), 7.37 (1H, s, H-8); ^13^C NMR (200 MHz, CD_3_OD) δ_C_ 23.2 (2C, C-17), 36.6 (C-16), 41.7 (C-9), 56.31 (6-OMe), 56.33 (7-OMe), 56.4 (12-OMe), 56.5 (13-OMe), 105.4 (C-8), 106.5 (C-5), 113.1 (C-14), 113.4 (C-11), 115.3 (C-4), 121.8 (C-15), 123.0 (C-8a), 133.7 (C-10), 136.2 (C-4a), 149.0 (C-13), 159.3 (C-3), 151.0 (C-12), 151.8 (C-7), 154.4 (C-6), 158.7 (C-1); (+)-LRESIMS *m*/*z* (rel. int.) 382 (100) [M + H]^+^; (+)-HRESIMS *m*/*z* 382.2024 [M + H]^+^ (calcd for C_23_H_28_NO_4_, 382.2013).

#### 3.3.3. Compound **8**

Colorless gum (1.3 mg, 4%); UV (MeOH) λ_max_ (log ε) 241 (3.90), 326 (2.94) nm; IR (UATR) ν_max_ 3456, 2981, 1745, 1705, 1279, 1199, 1167, 1079, 1026, 978, 712 cm^−1^; ^1^H NMR (800 MHz, CD_3_OD) δ_H_ 3.72 (3H, s, 13-OMe), 3.84 (3H, s, 7-OMe), 3.96 (3H, s, 6-OMe), 4.52 (2H, s, H-9), 7.15 (2H, m, H-11, H-15), 7.26 (1H, s, H-5), 7.39 (1H, s, H-8), 6.81 (2H, m, H-12, H-14), 7.58 (1H, d, *J* = 5.7 Hz, H-4), 8.20 (1H, d, *J* = 5.7 Hz, H-3); ^13^C NMR (200 MHz, CD_3_OD) δ_C_ 41.6 (C-9), 55.6 (13-OMe), 56.3 (7-OMe), 56.5 (6-OMe), 105.6 (C-8), 106.6 (C-5), 120.5 (C-4), 124.2 (C-8a), 130.4 (2C, C-11, C-15), 115.0 (2C, C-12, C-14), 135.5 (C-4a), 132.7 (C-10), 140.5 (C-3), 151.7 (C-7), 154.6 (C-6), 159.7 (C-1), 159.3 (C-13); (+)-LRESIMS *m*/*z* (rel. int.) 310 (100) [M + H]^+^; (+)-HRESIMS *m*/*z* 310.1436 [M + H]^+^ (calcd for C_19_H_20_NO_3_, 310.1438).

#### 3.3.4. Compound **10**

Colorless gum (4.5 mg, 19%); UV (MeOH) λ_max_ (log ε) 237 (3.91), 324 (2.83) nm; IR (UATR) ν_max_ 3413, 2986, 1747, 1709, 1281, 1198, 1169, 1078, 1026, 973, 712 cm^−1^; ^1^H NMR (800 MHz, CD_3_OD) δ_H_ 1.40 (6H, d, *J* = 6.8 Hz, H-10), 3.91 (1H, sept, *J* = 6.8 Hz, H-9), 3.97 (1H, s, 6-OMe), 3.98 (3H, s, 7-OMe), 7.23 (1H, s, H-5), 7.47 (1H, d, *J* = 5.6 Hz, H-4), 7.51 (1H, s, H-8), 8.17 (1H, d, *J* = 5.6 Hz, H-3); ^13^C NMR (200 MHz, CD_3_OD) δ_C_ 22.4 (2C, C-10, C-11), 31.9 (C-9), 56.4 (2C, 6-OMe, 7-OMe), 104.4 (C-8), 106.8 (C-5), 119.6 (C-4), 123.3 (C-8a), 135.1 (C-4a), 140.8 (C-3), 151.8 (C-7), 154.4 (C-6), 165.1 (C-1); (+)-LRESIMS *m*/*z* (rel. int.) 232 (100) [M + H]^+^; (+)-HRESIMS *m*/*z* 232.1333 (calcd for C_14_H_18_NO_2_, 232.1332).

### 3.4. Synthesis of Papaverine N-oxide (**12**)

Using a method previously reported by Bremner et al. [40], the free base of papaverine (**1b**, 0.50 g, 1.5 mmol) was dissolved in CHCl_3_ (10 mL) and treated portion wise with MCPBA (0.35 g, 2.4 mmol) over 5 min. After completion of the addition, the solution was stirred at room temperature for 19 h. The colorless precipitate that formed was filtered and the filtrate was extracted with 5% NaOH (3 × 25 mL). The CHCl_3_-soluble material was dried down then recrystallized from acetone to yield pure papaverine *N*-oxide (**12**, 310 mg, 58%).

Comparison of the ^1^H NMR and LRMS data of product **12** with literature values confirmed the compound [40], although the ^1^H NMR chemical shifts for two methoxyl signals had been incorrectly assigned to δ_H_ 6.14 and 6.15; this being a typographical error. The ^1^H NMR data for papaverine *N*-oxide was initially reported in CDCl_3_ at 60 MHz; no ^13^C NMR data was reported in the original article [40], hence we recorded 1D and 2D NMR data for **12** in both CDCl_3_ and CD_3_OD, and report the full NMR assignments here for completeness.

#### Compound **12**

White amorphous solid (310 mg, 58%); ^1^H NMR (800 MHz, CDCl_3_) δ_H_ 3.800*^a^* (3H, s, 12-OMe), 3.803*^a^* (3H, s, 13-OMe), 3.94 (3H, s, 7-OMe), 3.99 (3H, s, 6-OMe), 4.73 (2H, s, H-9), 6.73 (1H, d, *J* = 8.3 Hz, H-14), 6.80 (1H, dd, *J* = 8.3, 2.1 Hz, H-15), 7.01 (1H, d, *J* = 2.1 Hz, H-11), 7.03 (1H, s, H-5), 7.21 (1H, s, H-8), 7.42 (1H, d, *J* = 7.0 Hz, H-4), 8.16 (1H, d, *J* = 7.0 Hz, H-3); ^13^C NMR (200 MHz, CDCl_3_) δ_C_ 31.8 (C-9), 56.00*^b^* (12-OMe), 56.01*^b^* (13-OMe), 56.2 (7-OMe), 56.3 (6-OMe), 103.1 (C-8), 106.1 (C-5), 111.4 (C-14), 112.3 (C-11), 120.4 (C-15), 121.1 (C-4), 124.9 (C-8a), 125.7 (C-4a), 130.1 (C-10), 135.3 (C-3), 145.8 (C-1), 147.9 (C-13), 149.3 (C-12), 151.3 (C-6), 151.8 (C-7); ^1^H NMR (800 MHz, CD_3_OD) δ_H_ 3.747*^b^* (3H, s, 12-OMe), 3.748*^b^* (3H, s, 13-OMe), 3.91 (3H, s, 6-OMe), 3.96 (3H, s, 7-OMe), 4.79 (2H, s, H-9), 6.79 (1H, dd, *J* = 8.3, 1.8 Hz, H-15), 6.82 (1H, d, *J* = 8.3 Hz, H-14), 7.01 (1H, d, *J* = 1.8 Hz, H-11), 7.33 (1H, s, H-5), 7.38 (1H, s, H-8), 7.72 (1H, d, *J* = 7.0 Hz, H-4), 8.14 (1H, d, *J* = 7.0 Hz, H-3); ^13^C NMR (200 MHz, CD_3_OD) δ_C_ 32.2 (C-9), 56.44*^c^* (12-OMe), 56.47*^c^* (13-OMe), 56.63*^d^* (7-OMe), 56.66*^d^* (6-OMe), 104.8 (C-8), 107.3 (C-5), 113.2 (C-14), 113.8 (C-11), 121.9 (C-15), 123.2 (C-4), 125.6 (C-8a), 129.1 (C-4a), 131.3 (C-10), 134.8 (C-3), 148.4 (C-1), 149.4 (C-13), 150.7 (C-12), 153.6 (C-7), 154.1 (C-6); (+)-LRESIMS *m*/*z* (rel. int.) 356 (100) [M + H]^+^. *^a,b,c,d^* Interchangeable signals.

### 3.5. Synthesis of Papaverine *N*-oxide Diversinate^TM^ Analogue (**13**)

Papaverine *N*-oxide (**12**, 35.5 mg, 0.1 mmol) was dissolved in CH_2_Cl_2_/H_2_O (2.5:1, 100 μL:40 μL) before the addition of (CF_3_SO_2_)_2_Zn (198.9 mg, 0.6 mmol) at room temperature. The mixture was cooled to 0 °C before TBHP (40 μL, 0.6 mmol) was slowly added, after which time, the stirred mixture slowly warmed to room temperature over 16 h. The crude reaction product was dried under N_2_ and pre-adsorbed to C_18_-bonded silica (≈1 g) overnight with the dry material packed into a stainless-steel guard cartridge, which was subsequently attached to a C_18_ semi-preparative HPLC column. Isocratic conditions of H_2_O/MeOH/TFA (90:10:0.1) were run for the first 10 min, followed by a linear gradient of MeOH/TFA (100:0.1) over 40 min, then isocratic conditions of MeOH/TFA (100:0.1) for a further 10 min, all at a flow rate of 9 mL/min. Sixty fractions (60 × 1 min) were collected from the start of the HPLC run. Fractions containing UV-active material were analyzed using ^1^H NMR spectroscopy and LCMS in order to identify products of interest. The starting material (**12**, 9.3 mg, 26%) eluted in fraction 39, while a mixture (<80% pure) containing predominantly the mono-CF_3_ papaverine *N*-oxide analogue (**13**, 5.1 mg, <12%) eluted in fraction 43. While ^1^H NMR and LCMS analysis of the fraction 43 mixture indicated product **13** had been made, the paucity of material and low yield, meant no further purification or characterization was undertaken.

### 3.6. Generation of the Free Base of Papaverine (**1b**)

Papaverine hydrochloride (**1a**, 200 mg) was dissolved in a mixture of ammoniated H_2_O (9:1, H_2_O:28% aq. NH_3_, 20 mL) and then extracted with CH_2_Cl_2_ (3 × 20 mL). The free base of papaverine (**1b**) was soluble in the CH_2_Cl_2_ layer, and following evaporation, yielded the desired product with a high purity and yield (174 mg, 93%).

### 3.7. Generation of Compounds **2** and **3** Using the Free Base of Papaverine (**1b**)

The free base of papaverine (**1b**, 33.9 mg, 0.1 mmol) was dissolved in CH_2_Cl_2_/H_2_O or DMSO/H_2_O (2.5:1, 100 μL:40 μL) before the addition of the (CF_3_SO_2_)_2_Zn (198.9 mg, 0.6 mmol) at room temperature. Where TFA was used, 7.7 μL (0.1 mmol) was added to the reaction while stirring. The mixture was cooled to 0 °C before TBHP (40 μL, 0.6 mmol) was added, after which time, the stirred mixture was slowly warmed to room temperature over 16 h. The crude reaction product was dried under N_2_ and pre-adsorbed to NH_2_-bonded silica (≈1 g) overnight before being subjected to identical NH_2_ semi-preparative HPLC conditions, which are described above. Reaction conditions and yields for these free base reactions can be found in Table S71.

## 4. Conclusions

The papaverine scaffold reacted with (CF_3_SO_2_)_2_Zn and TBHP to give dimethoxybenzyl ring-substituted products. With other sulfinates, however, the products featured substitution on the isoquinoline ring. The use of nucleophilic radical precursors, such as zinc isopropylsulfinate, resulted in the replacement of the 3,4-dimethoxybenzyl substituent as the major reaction. A mechanism was suggested for this unusual radical replacement reaction. Despite the fact that the papaverine scaffold was a poor substrate for Diversinate^TM^ chemistry resulting in low yields, we were able to prepare a small library of ten papaverine derivatives (including seven new compounds), some of which would be difficult to prepare by other means. Oxidizing the scaffold to papaverine *N*-oxide had no significant impact on the Diversinate^TM^ yield or selectivity. To the best of our knowledge, these are the first examples of the derivatization of a benzylisoquinoline using Diversinate^TM^ C–H functionalization chemistry. This unique library has been added to the Davis open-access natural product-based library for future drug discovery and chemical biology evaluations [41].

## Figures and Tables

**Table 1:** ^1^H (800 MHz) and ^13^C (200 MHz) NMR data for the mono-CF_3_ papaverine analogues **2** and **3** in CD_3_OD at 25 °C.

Position	Mono-CF_3_ Analogue 2		Mono-CF_3_ Analogue 3	
	δ_C_, type, (*J* in Hz)	δ_H_, mult. (*J* in Hz)	δ_C_, type, (*J* in Hz)	δ_H_, mult. (*J* in Hz)
1	157.6, C		158.2, C	
3	140.7, CH	8.25, d (5.7)	140.7, CH	8.23, d (5.7)
4	120.9, CH	7.65, d (5.7)	120.8, CH	7.62, d (5.7)
4a	135.4, C		135.6, C	
5	106.7, CH	7.30, s	106.7, CH	7.30, s
6	154.7, C		154.7, C	
7	152.1, C		152.0, C	
8	104.5, CH	7.19, s	105.1, CH	7.46, s
8a	124.3, C		124.2, C	
9	38.4, CH_2_	4.67, s	41.7, CH_2_	4.60, s
6-OMe	56.8, CH_3_	3.98, s	56.51, CH_3_	3.98, s
7-OMe	56.5, CH_3_	3.78, s	56.4, CH_3_	3.91, s
12-OMe	56.1, CH_3_	3.49, s	56.54, CH_3_	3.81, s
13-OMe	56.2, CH_3_	3.85, s	61.7, CH_3_	3.80, s
10	132.3, C, q, (2.1)		137.1, C	
11	114.5, CH	6.41, s	117.9, CH	7.22, d (2.2)
12	153.1, C		154.9, C	
13	148.8, C		147.3, C, q, (2.3)	
14	110.7, CH, q, (5.9)	7.26, s	120.8, C, q, (39.2)	
15	121.2, C, q, (30.3)		118.6, CH, q, (5.2)	7.11, d (2.2)
CF_3_	126.4, C, q, (272.1)		124.8, C, q, (269.4)	

## Supplementary Materials

The following are available online: 1D/2D NMR spectra for compounds **1**–**12** and Diversinate^TM^ optimization conditions on papaverine HCl salt and papaverine free base.
