# Supplementary material for: Reaction of Papaverine with Baran DiversinatesTM
Source: Molecules. 2019 Oct 31;24(21):3938. doi: 10.3390/molecules24213938 (PMC6864744; doi:10.3390/molecules24213938)

Supplementary data contents page

Reaction of Papaverine with Baran Diversinates™

Folake A. Egbewande, Mark J. Coster, Ian D. Jenkins and Rohan A. Davis*

Griffith Institute for Drug Discovery, Griffith University, Brisbane, QLD 4111, Australia.

Corresponding Author Contact Details:
Tel.: +61 7 3735 6043. Fax: +61 7 3735 6001. E-mail: r.davis@griffith.edu.au

Contents:

S1: ^{1}H NMR Spectrum for Compound **1a** in CD_3OD……………………………………4

S2: ^{13}C NMR Spectrum for Compound **1a** in CD_3OD..4

S3: COSY NMR Spectrum for Compound **1a** in CD_3OD 5

S4: HSQC NMR Spectrum for Compound **1a** in CD_3OD 5

S5: HMBC NMR Spectrum for Compound **1a** in CD_3OD 6

S6: ^{1}H NMR Spectrum for Compound **1b** in CD_3OD 7

S7: ^{13}C NMR Spectrum for Compound **1b** in CD_3OD 7

S8: COSY NMR Spectrum for Compound **1b** in CD_3OD 8

S9: HSQC NMR Spectrum for Compound **1b** in CD_3OD 8

S10: HMBC NMR Spectrum for Compound **1b** in CD_3OD 9

S11: ^{1}H NMR Spectrum for Compound **2** in CD_3OD...10

S12: ^{13}C NMR Spectrum for Compound **2** in CD_3OD...10

S13: COSY NMR Spectrum for Compound **2** in CD_3OD 11

S14: HSQC NMR Spectrum for Compound **2** in CD_3OD 11

S15: HMBC NMR Spectrum for Compound **2** in CD_3OD 12

S16: ^{1}H NMR Spectrum for Compound **3** in CD_3OD...13

S17: ^{13}C NMR Spectrum for Compound **3** in CD_3OD...13

S18: COSY NMR Spectrum for Compound **3** in CD_3OD 14

S19: HSQC NMR Spectrum for Compound **3** in CD_3OD 14

S20: HMBC NMR Spectrum for Compound **3** in CD_3OD 15

S21: ^{1}H NMR Spectrum for Compound **4** in CD_3OD...16

S22: ^{13}C NMR Spectrum for Compound **4** in CD_3OD...16

S23: COSY NMR Spectrum for Compound **4** in CD_3OD 17
S24: HSQC NMR Spectrum for Compound **4** in CD_3OD 17
S25: HMBC NMR Spectrum for Compound **4** in CD_3OD 18
S26: ^{1}H NMR Spectrum for Compound **5** in CD_3OD 19
S27: ^{13}C NMR Spectrum for Compound **5** in CD_3OD 19
S28: COSY NMR Spectrum for Compound **5** in CD_3OD 20
S29: HSQC NMR Spectrum for Compound **5** in CD_3OD 20
S30: HMBC NMR Spectrum for Compound **5** in CD_3OD 21
S31: ^{1}H NMR Spectrum for Compound **6** in CD_3OD 22
S32: ^{13}C NMR Spectrum for Compound **6** in CD_3OD 22
S33: COSY NMR Spectrum for Compound **6** in CD_3OD 23
S34: HSQC NMR Spectrum for Compound **6** in CD_3OD 23
S35: HMBC NMR Spectrum for Compound **6** in CD_3OD 24
S36: ^{1}H NMR Spectrum for Compound **7** in CD_3OD 25
S37: ^{13}C NMR Spectrum for Compound **7** in CD_3OD 25
S38: COSY NMR Spectrum for Compound **7** in CD_3OD 26
S39: HSQC NMR Spectrum for Compound **7** in CD_3OD 26
S40: HMBC NMR Spectrum for Compound **7** in CD_3OD 27
S41: ^{1}H NMR Spectrum for Compound **8** in CD_3OD 28
S42: ^{13}C NMR Spectrum for Compound **8** in CD_3OD 28
S43: COSY NMR Spectrum for Compound **8** in CD_3OD 29
S44: HSQC NMR Spectrum for Compound **8** in CD_3OD 29
S45: HMBC NMR Spectrum for Compound **8** in CD_3OD 30
S46: ^{1}H NMR Spectrum for Compound **9** in CD_3OD 31
S47: ^{13}C NMR Spectrum for Compound **9** in CD_3OD 31
S48: COSY NMR Spectrum for Compound **9** in CD_3OD 32
S49: HSQC NMR Spectrum for Compound **9** in CD_3OD 32
S50: HMBC NMR Spectrum for Compound **9** in CD_3OD 33
S51: ^{1}H NMR Spectrum for Compound **10** in CD_3OD 34
S52: ^{13}C NMR Spectrum for Compound **10** in CD_3OD 34
S53: COSY NMR Spectrum for Compound **10** in CD_3OD 35
S54: HSQC NMR Spectrum for Compound **10** in CD_3OD 35
S55: HMBC NMR Spectrum for Compound **10** in CD_3OD 36

S56: ^{1}H NMR Spectrum for Compound **11** in CD_3OD.. 37
S57: ^{13}C NMR Spectrum for Compound **11** in CD_3OD.. 37
S58: COSY NMR Spectrum for Compound **11** in CD_3OD 38
S59: HSQC NMR Spectrum for Compound **11** in CD_3OD.. 38
S60: HMBC NMR Spectrum for Compound **11** in CD_3OD 39
S61: ^{1}H NMR Spectrum for Compound **12** in $CDCl_3$.. 40
S62: ^{13}C NMR Spectrum for Compound **12** in $CDCl_3$.. 40
S63: COSY NMR Spectrum for Compound **12** in $CDCl_3$ 41
S64: HSQC NMR Spectrum for Compound **12** in $CDCl_3$ 41
S65: HMBC NMR Spectrum for Compound **12** in $CDCl_3$ 42
S66: ^{1}H NMR Spectrum for Compound **12** in CD_3OD.. 43
S67: ^{13}C NMR Spectrum for Compound **12** in CD_3OD.. 43
S68: COSY NMR Spectrum for Compound **12** in CD_3OD 44
S69: HSQC NMR Spectrum for Compound **12** in CD_3OD.. 44
S70: HMBC NMR Spectrum for Compound **12** in CD_3OD 45
S71: Diversinate optimisation reactions with papaverine HCl (**1a**) and the free base of papaverine (**1b**) 46
S72: Stacked ^{1}H NMR (800 MHz) spectra of the HCl salt (top) and free-base (bottom) of papaverine in CD_3OD.. 46
S73: ^{1}H (800 MHz) and ^{13}C (200 MHz) NMR data for papaverine HCl (**1a**) and the free base of papaverine (**1b**) in CD_3OD at 25 °C.. 47
S74: Expansion of ^{13}C NMR spectrum of compound **2** showing the quartet of the CF_3 group.. 48
S75: Expansion of HMBC spectrum of compound **3**.. 48

S1: ^{1}H NMR Spectrum for Compound **1a** in CD_3OD

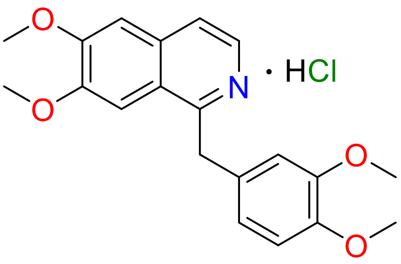

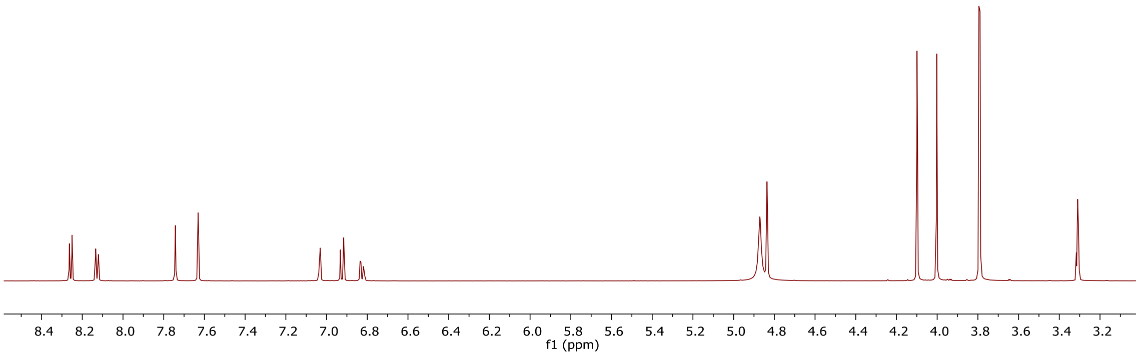

S2: ^{13}C NMR Spectrum for Compound **1a** in CD_3OD

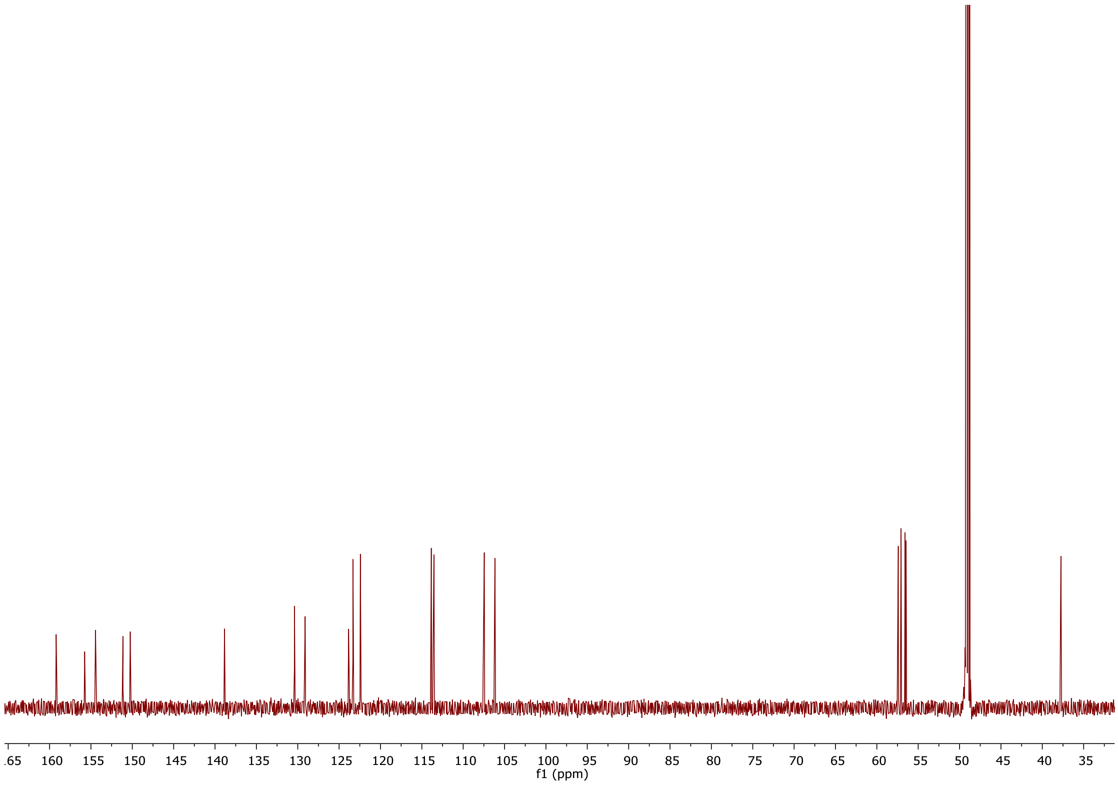

S3: COSY NMR Spectrum for Compound **1a** in CD_3OD

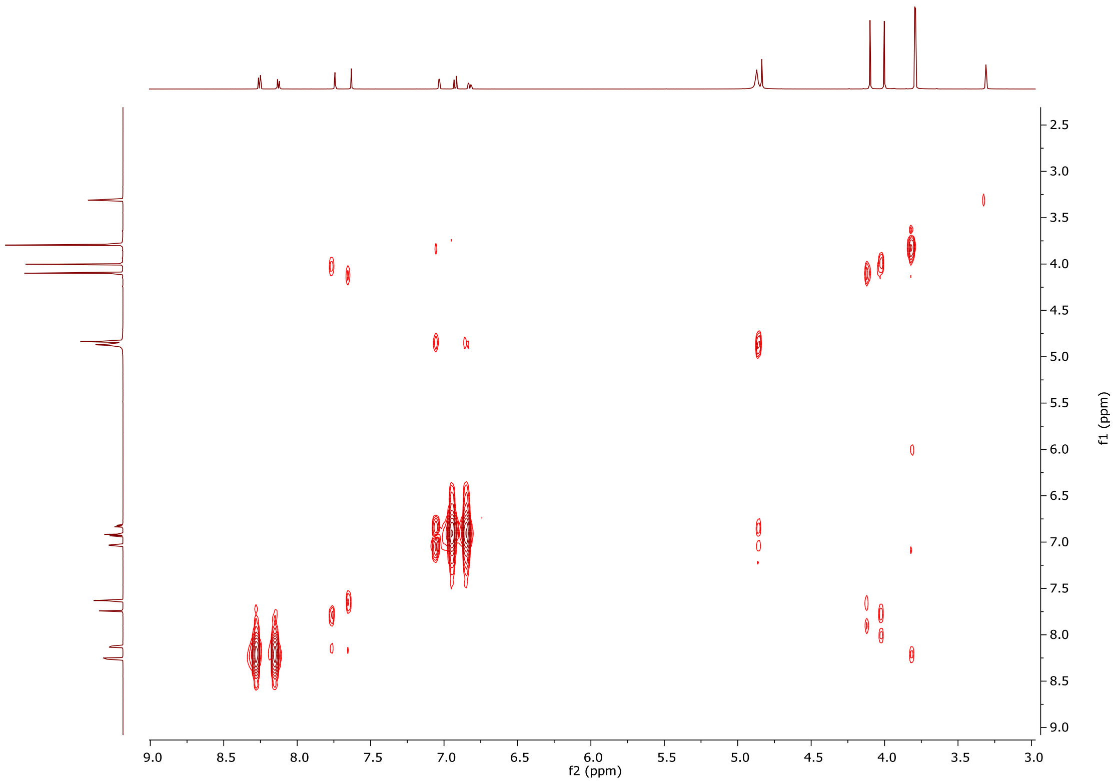

S4: HSQC NMR Spectrum for Compound **1a** in CD_3OD

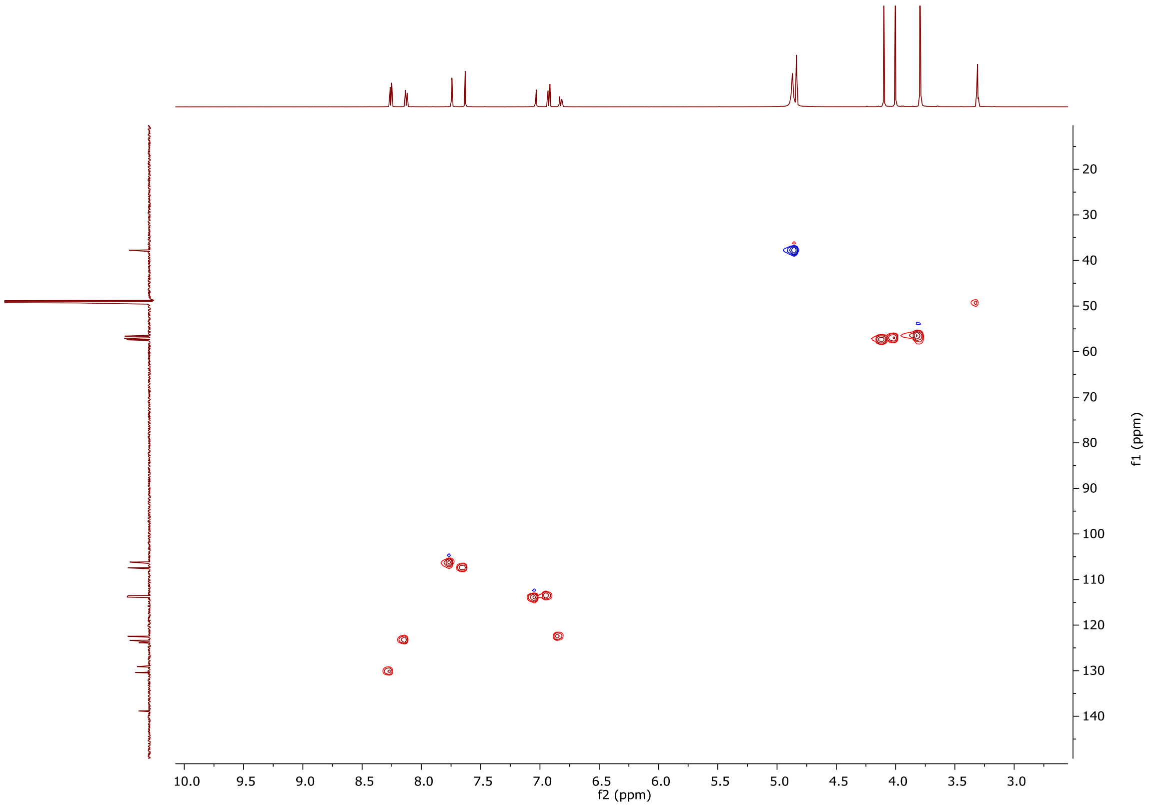

S5: HMBC NMR Spectrum for Compound **1a** in CD_3OD

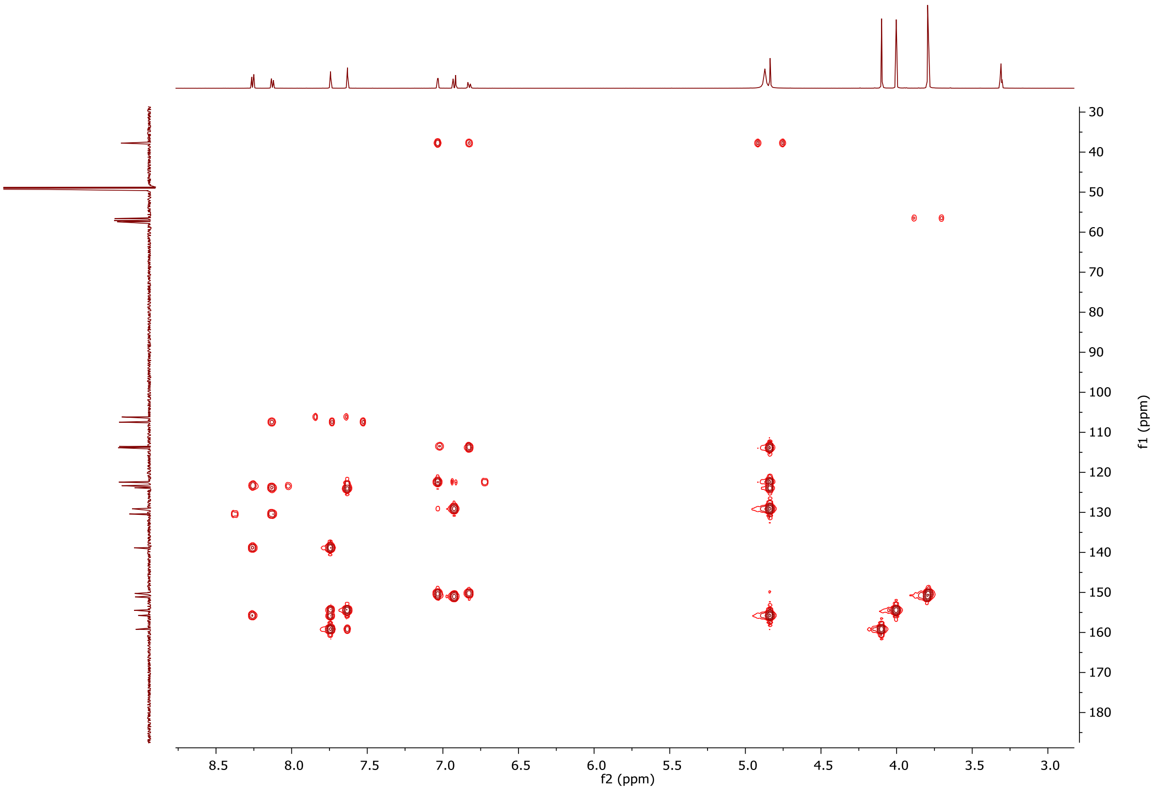

S6: ^{1}H NMR Spectrum for Compound **1b** in CD_3OD

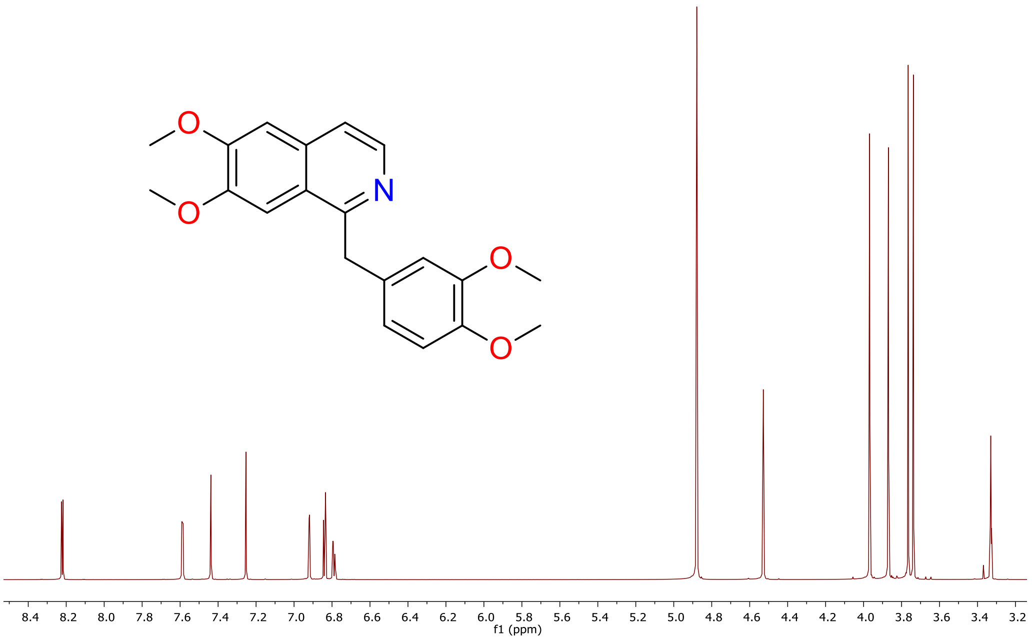

S7: ^{13}C NMR Spectrum for Compound **1b** in CD_3OD

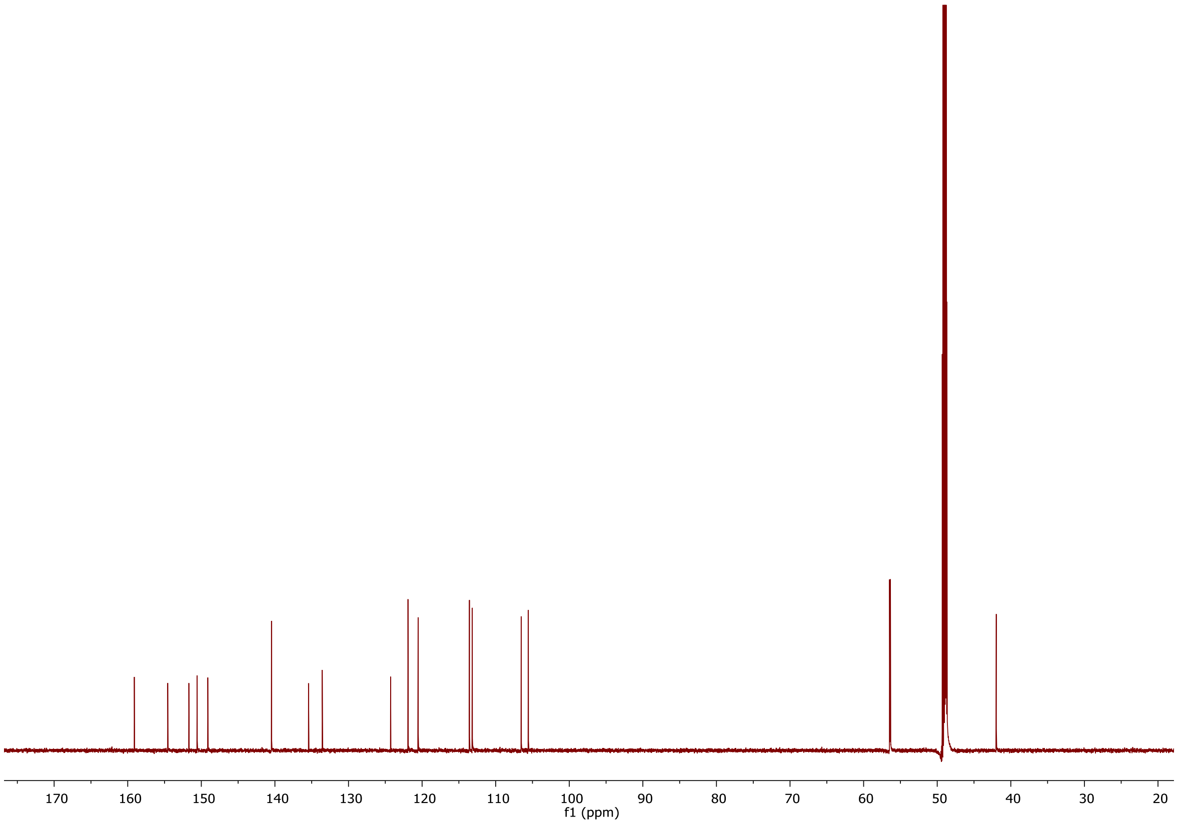

S8: COSY NMR Spectrum for Compound **1b** in CD_3OD

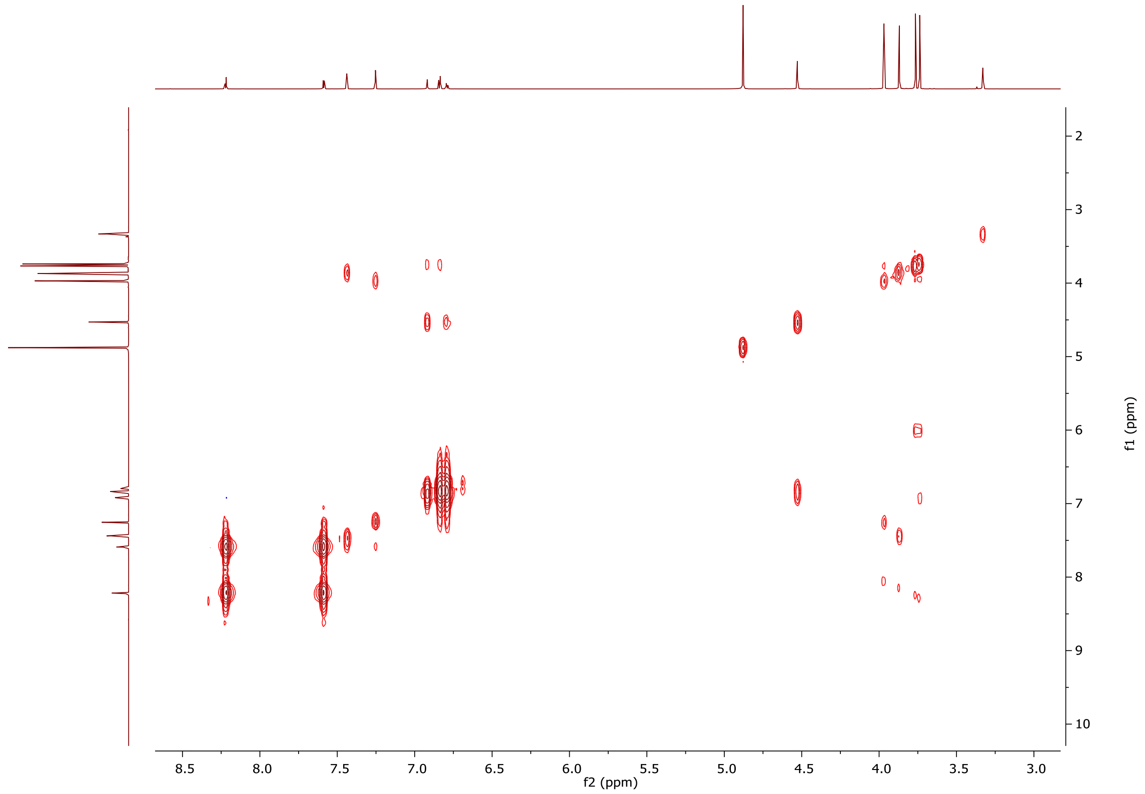

S9: HSQC NMR Spectrum for Compound **1b** in CD_3OD

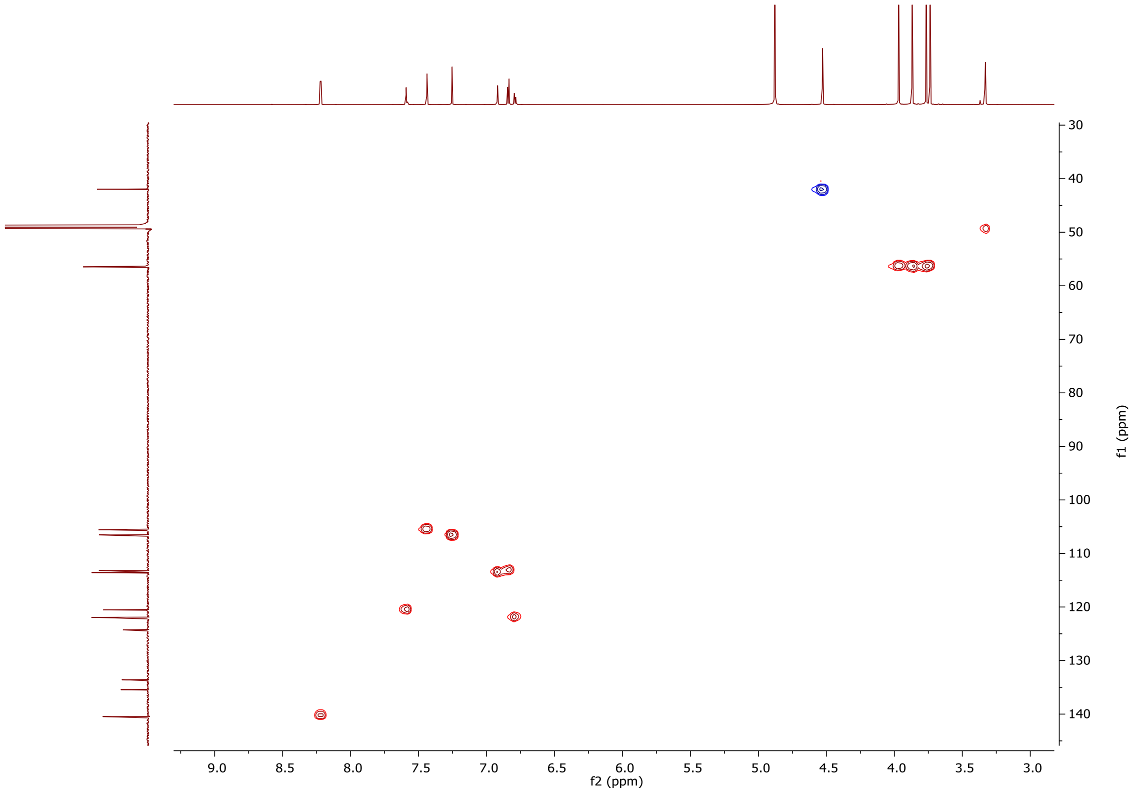

S10: HMBC NMR Spectrum for Compound **1b** in CD_3OD

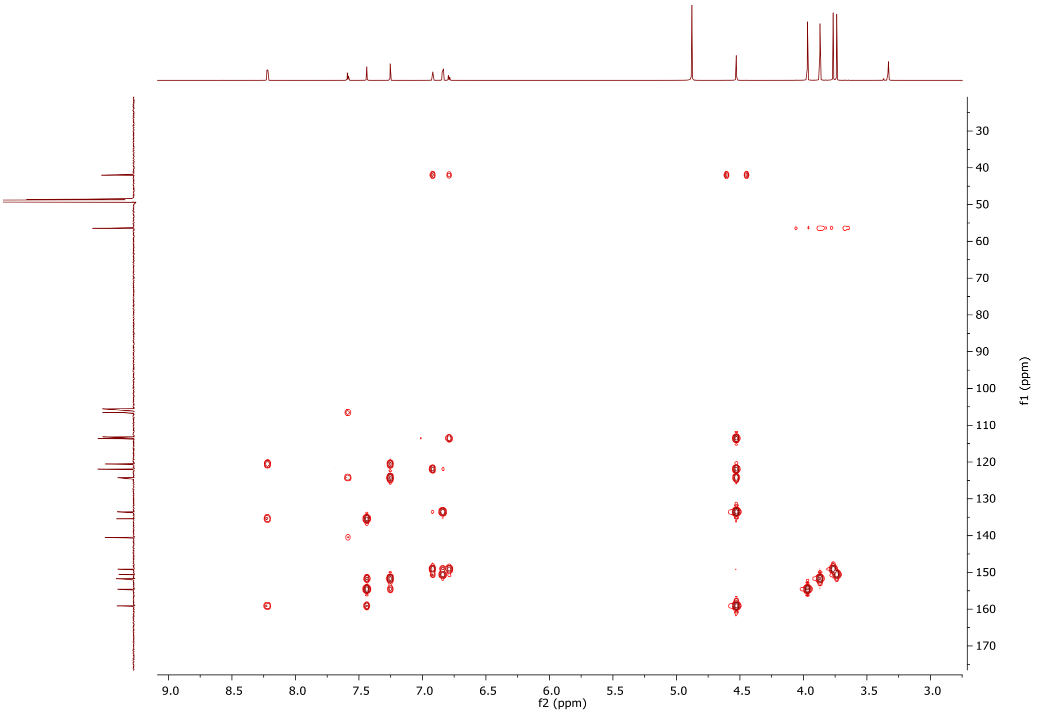

S11: ^{1}H NMR Spectrum for Compound **2** in CD_3OD

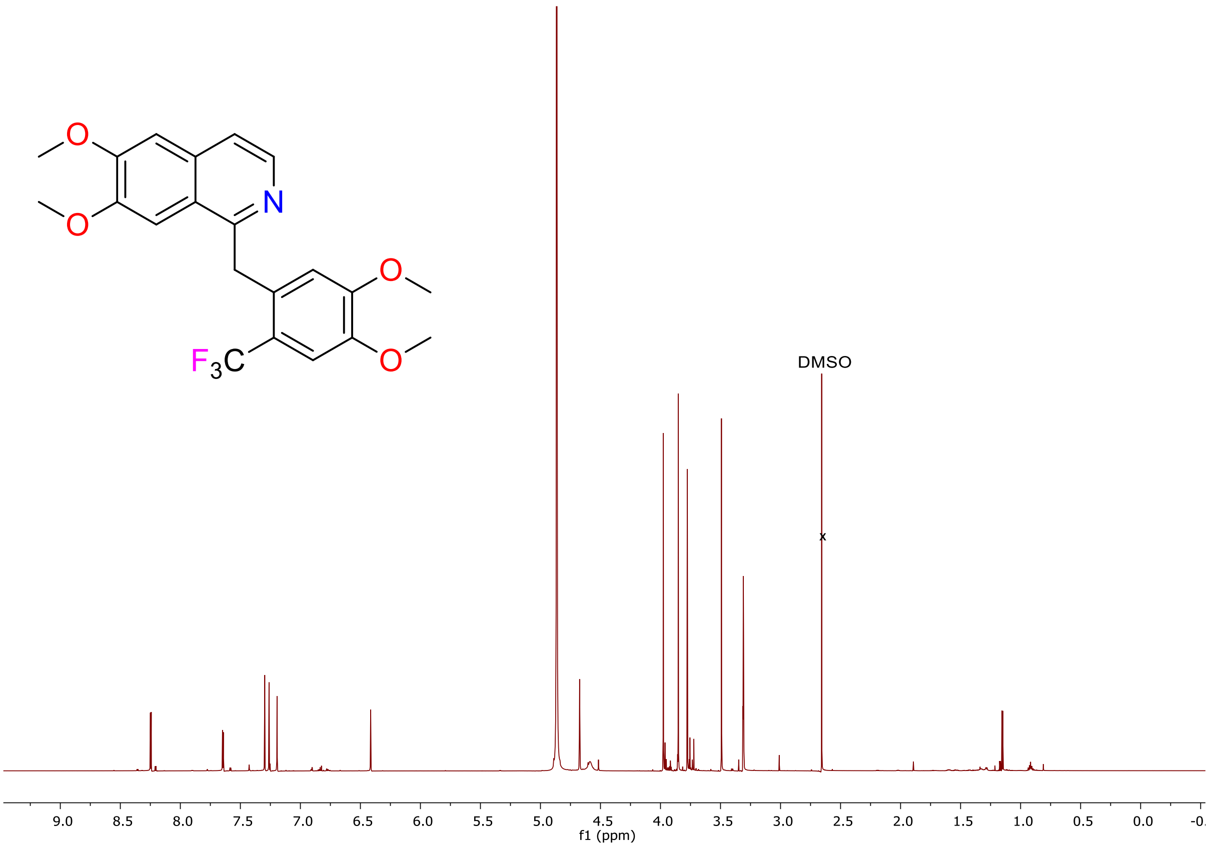

S12: ^{13}C NMR Spectrum for Compound **2** in CD_3OD

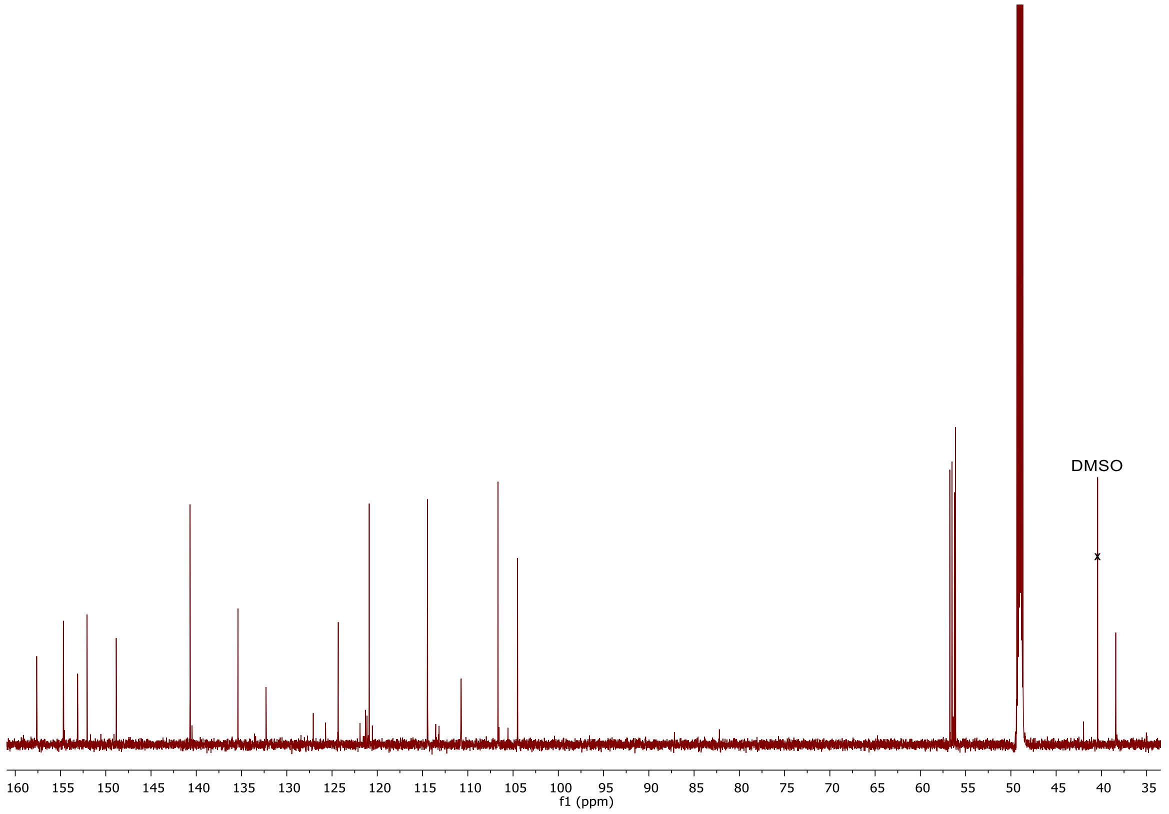

S13: COSY NMR Spectrum for Compound **2** in CD_3OD

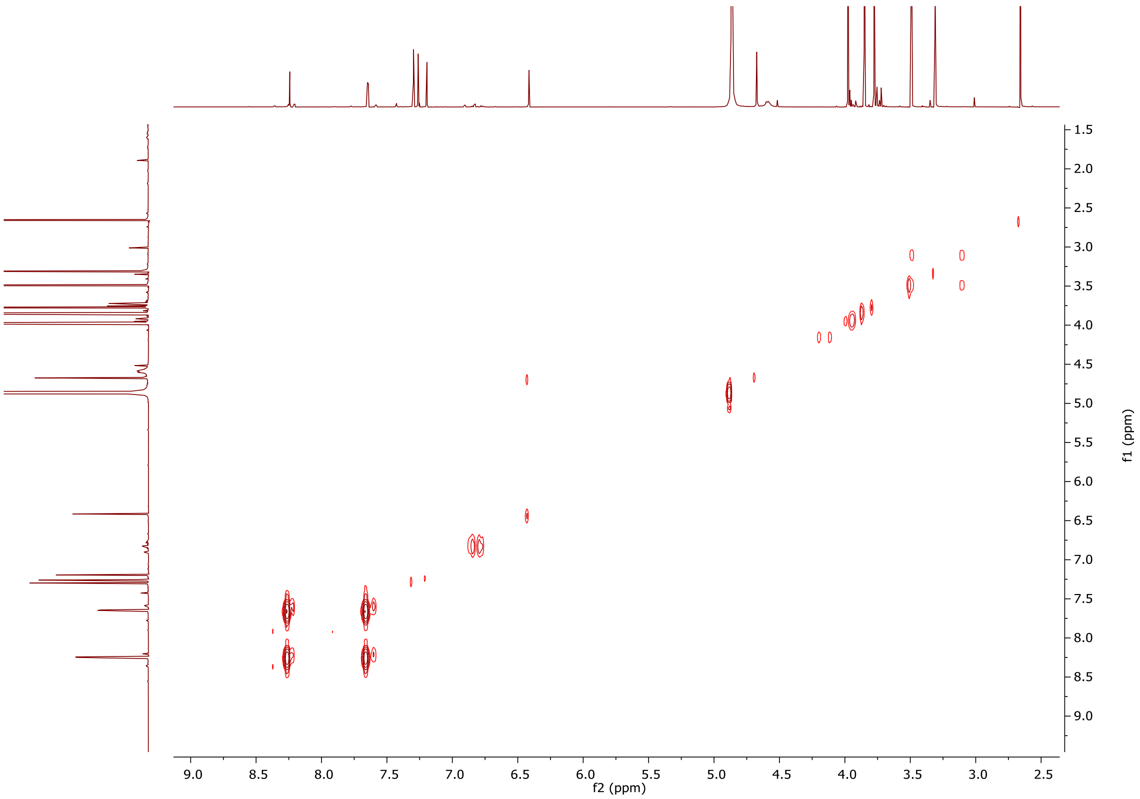

S14: HSQC NMR Spectrum for Compound **2** in CD_3OD

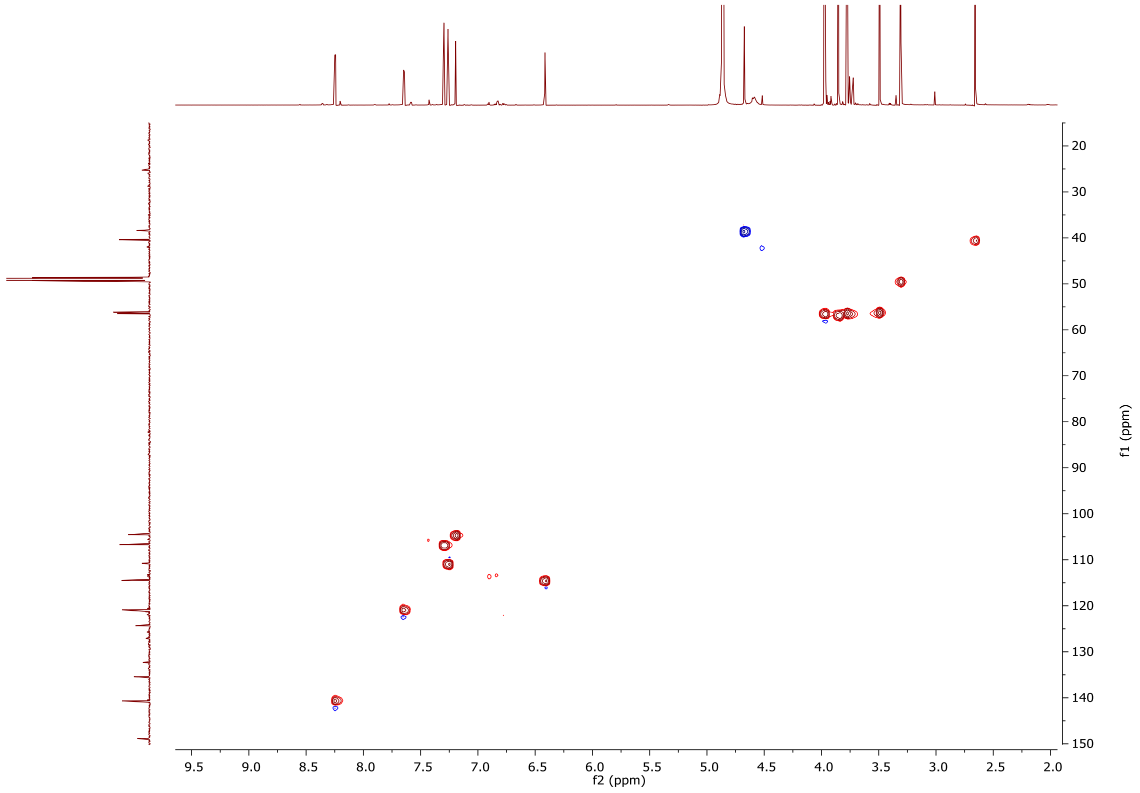

S15: HMBC NMR Spectrum for Compound **2** in CD_3OD

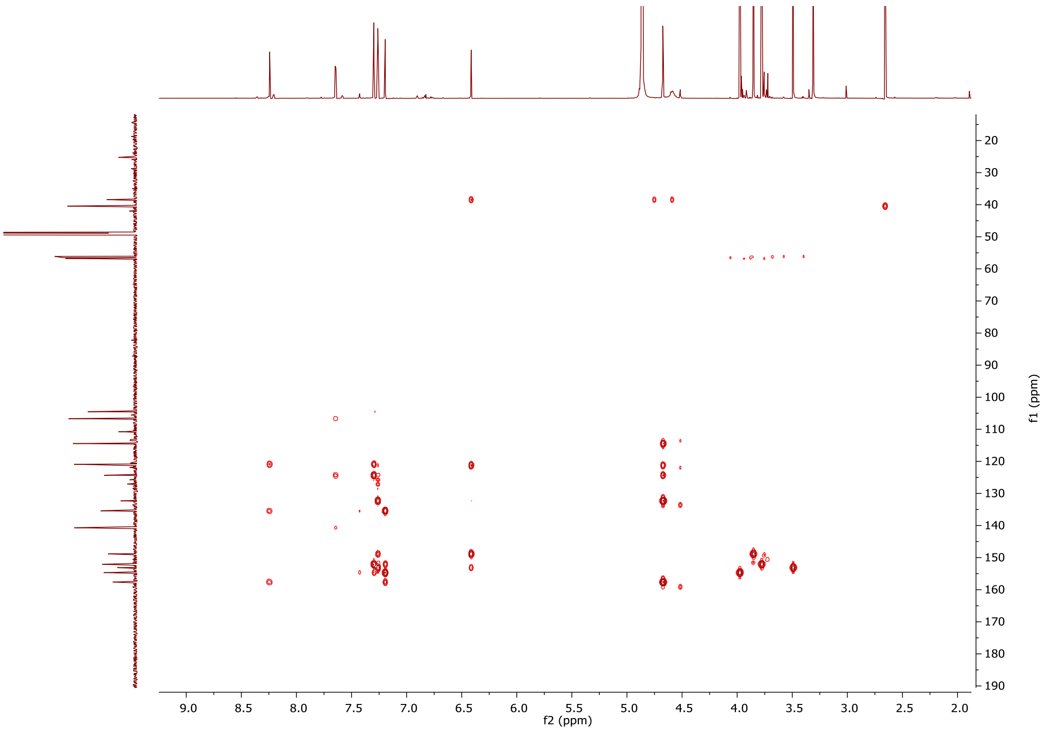

S16: ^{1}H NMR Spectrum for Compound **3** in CD_3OD

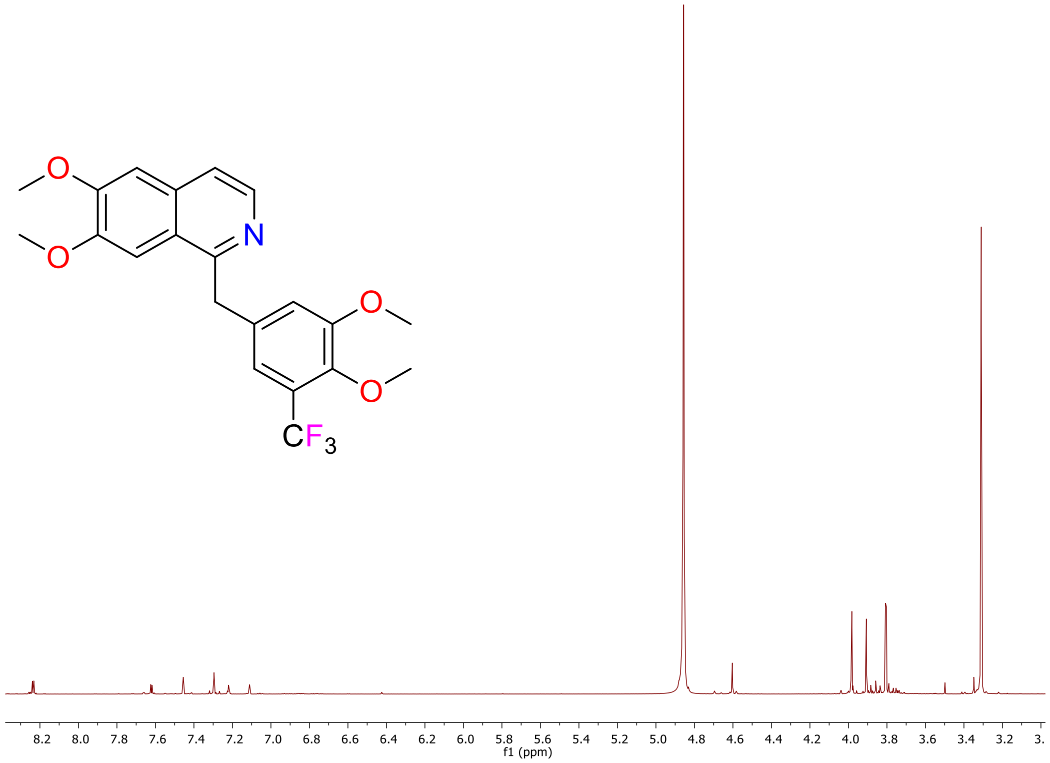

S17: ^{13}C NMR Spectrum for Compound **3** in CD_3OD

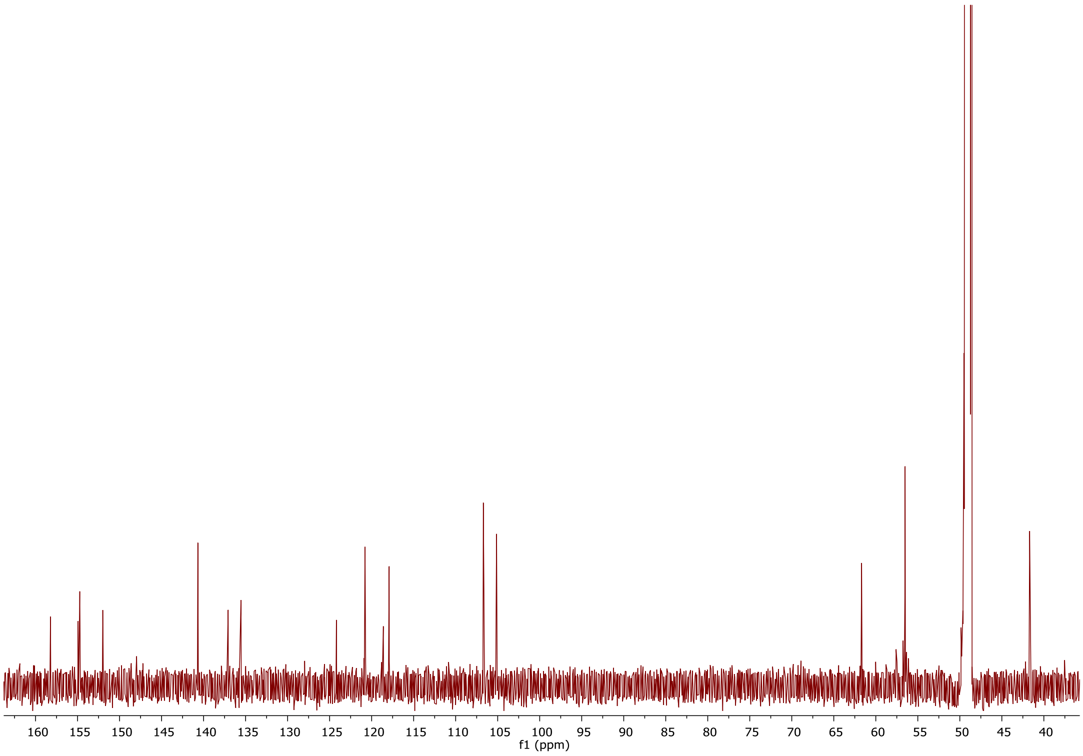

S18: COSY NMR Spectrum for Compound **3** in CD_3OD

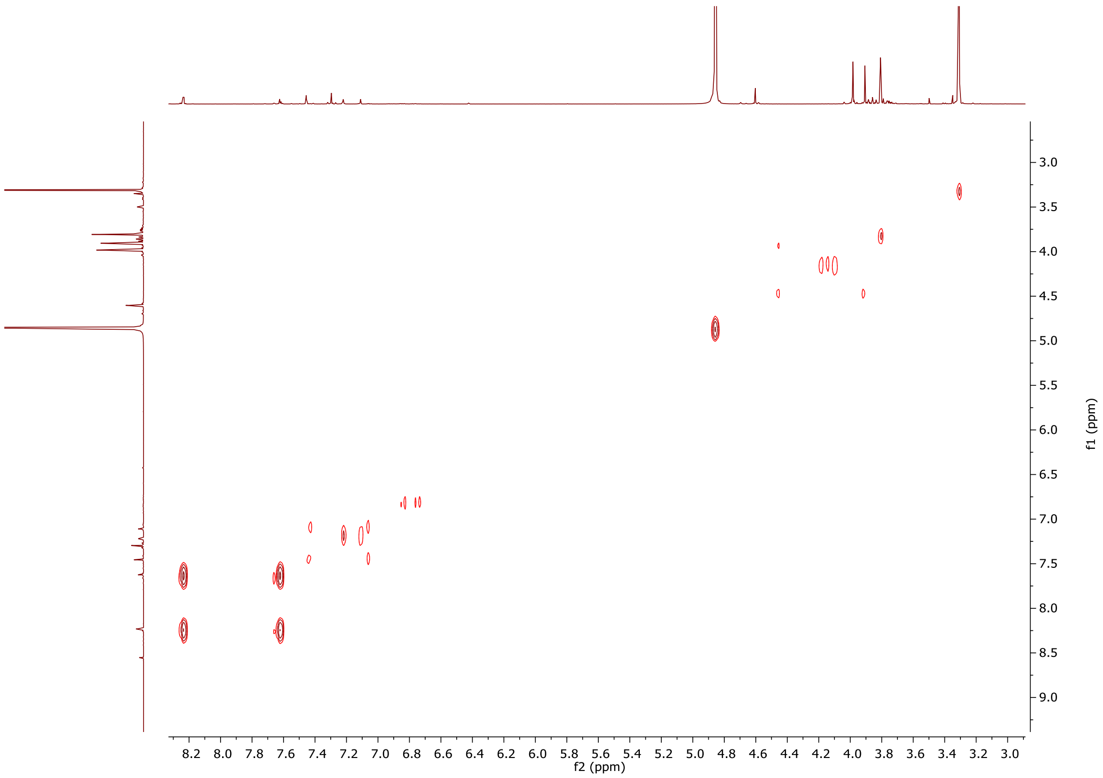

S19: HSQC NMR Spectrum for Compound **3** in CD_3OD

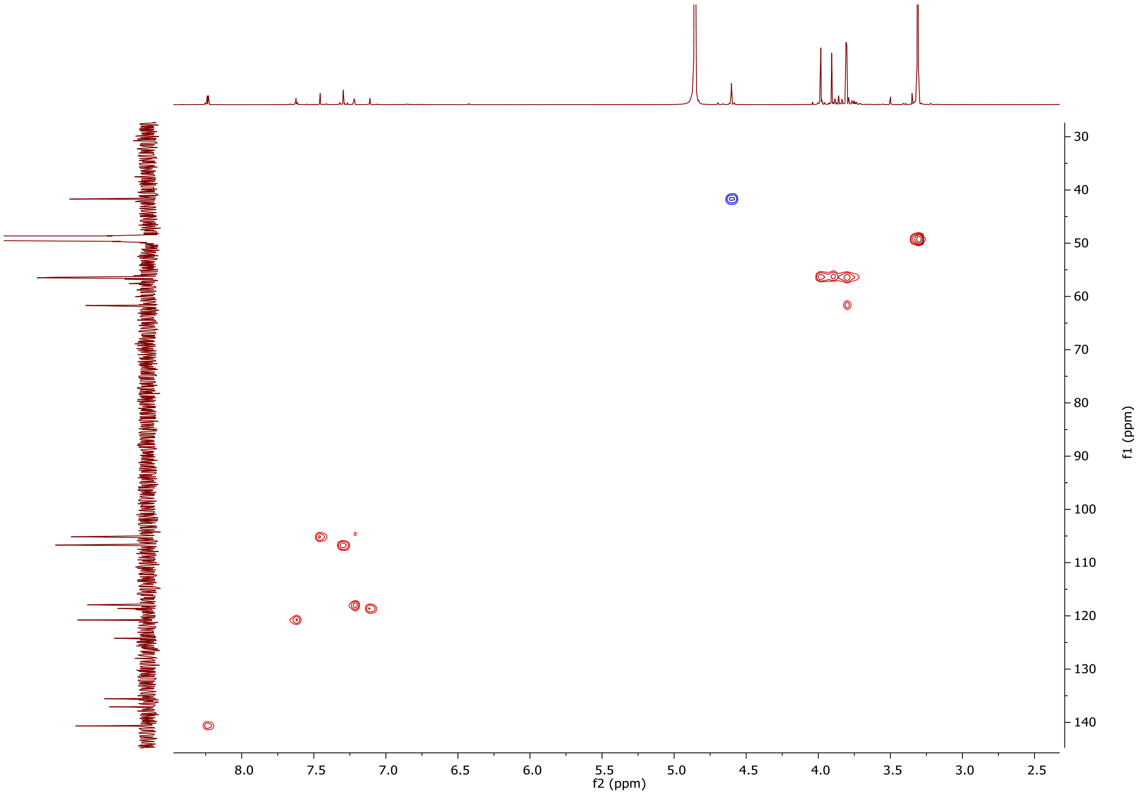

S20: HMBC NMR Spectrum for Compound **3** in CD_3OD

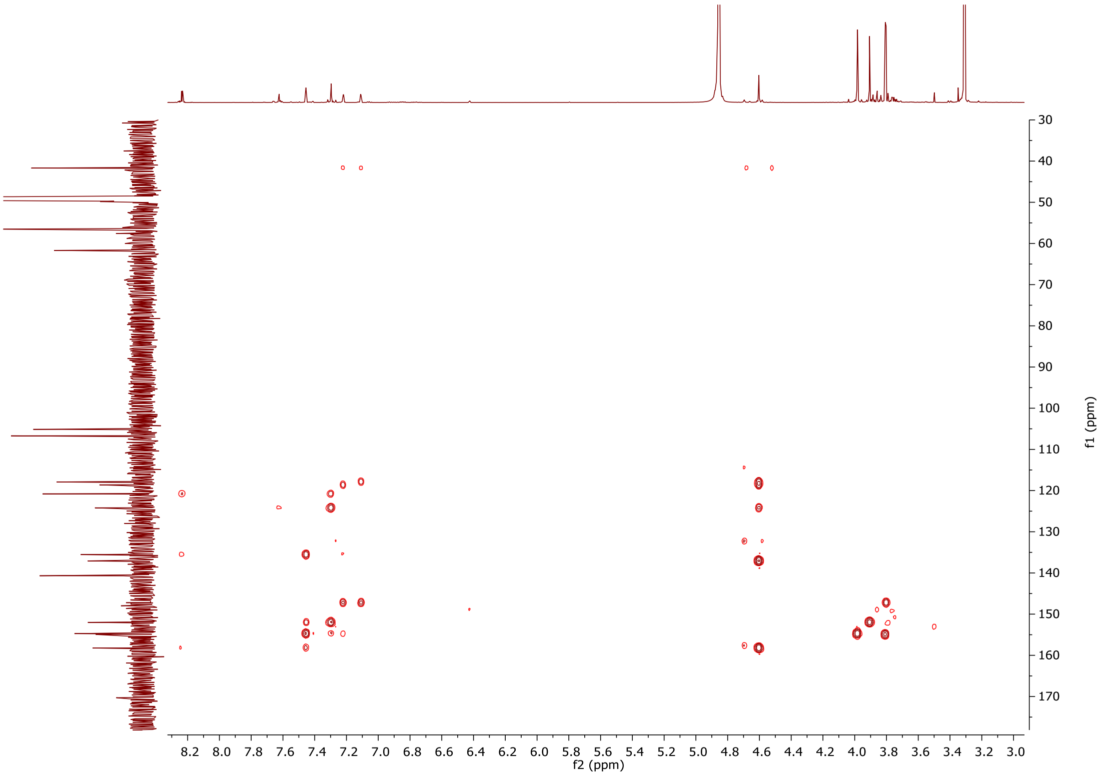

S21: ^{1}H NMR Spectrum for Compound **4** in CD_3OD

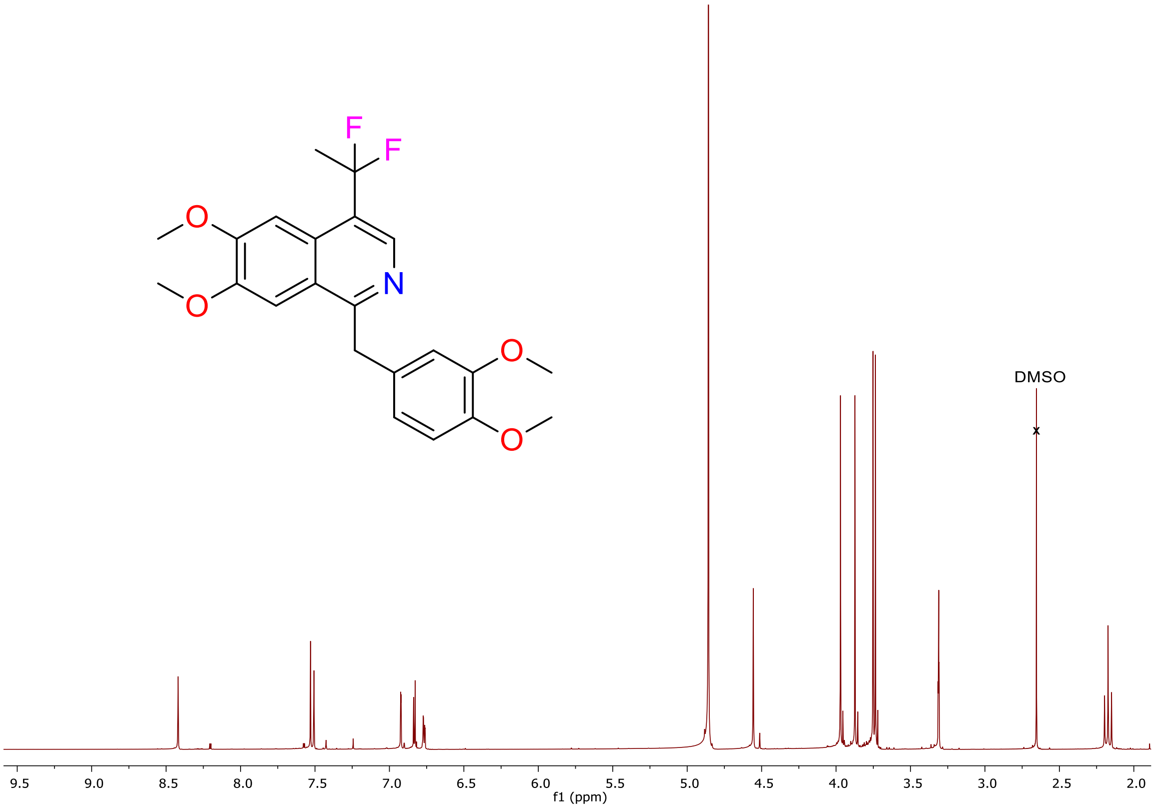

S22: ^{13}C NMR Spectrum for Compound **4** in CD_3OD

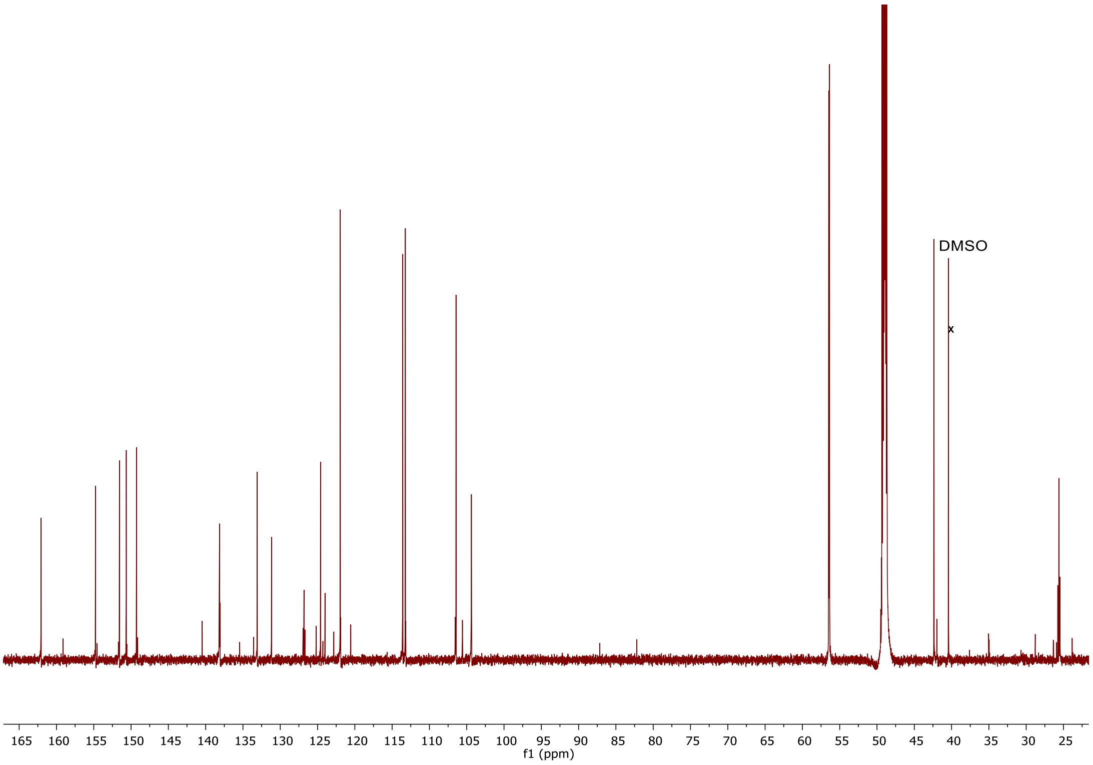

S23: COSY NMR Spectrum for Compound **4** in CD_3OD

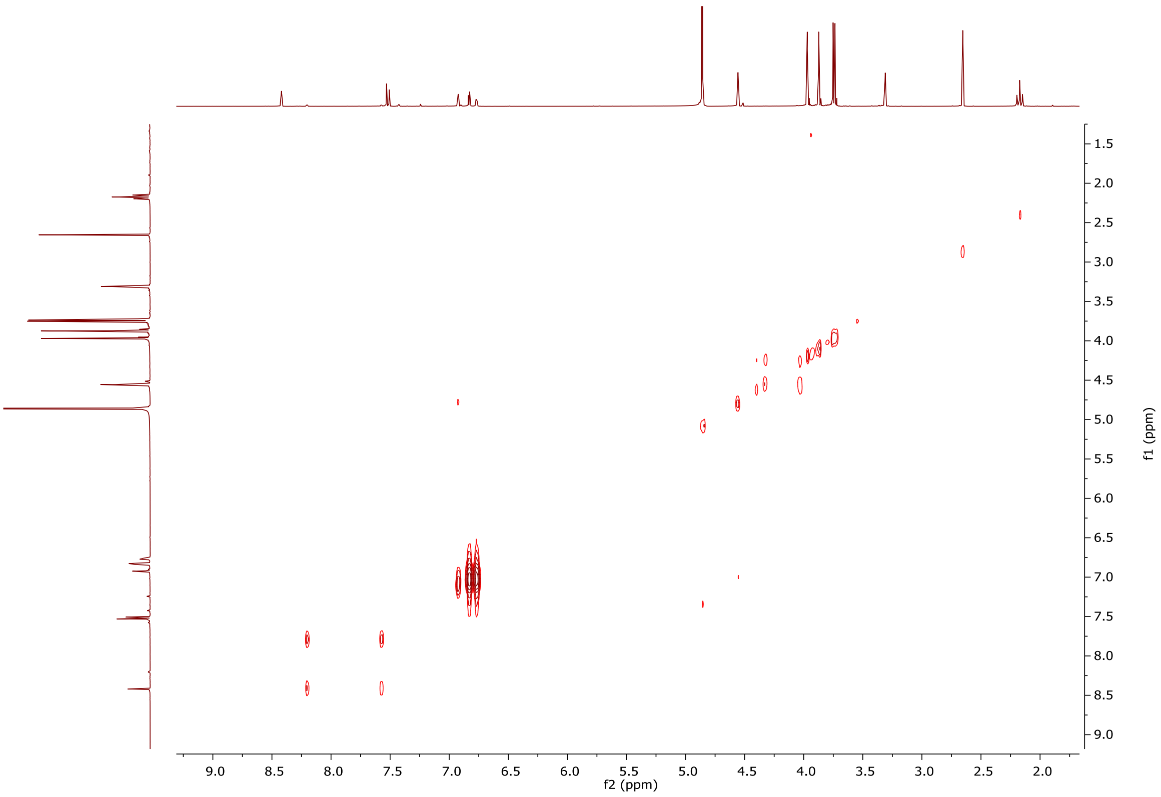

S24: HSQC NMR Spectrum for Compound **4** in CD_3OD

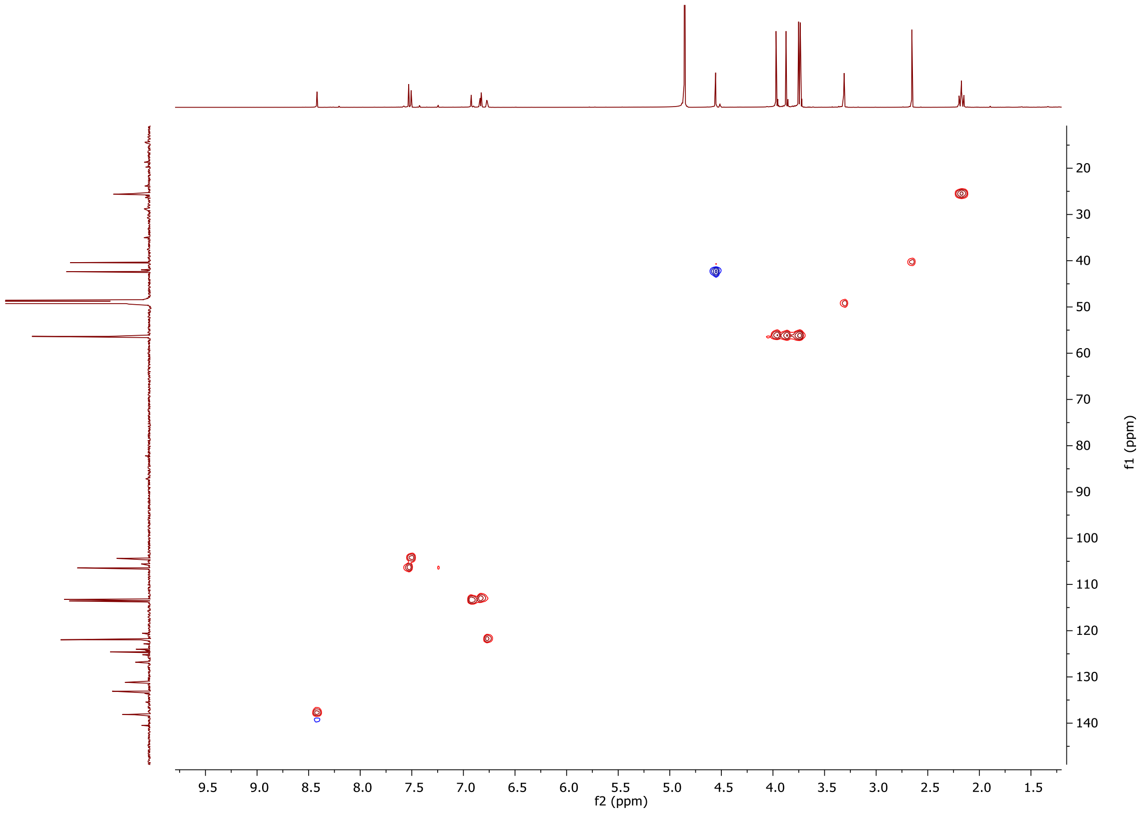

S25: HMBC NMR Spectrum for Compound **4** in CD_3OD

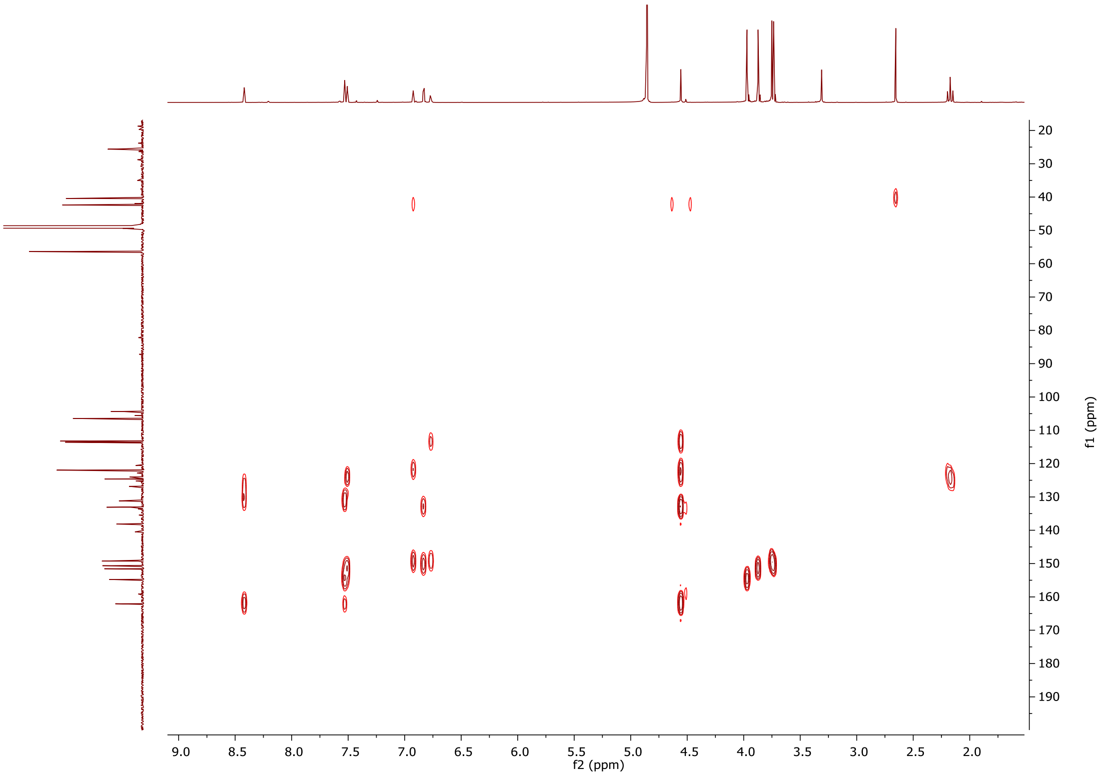

S26: ^{1}H NMR Spectrum for Compound **5** in CD_3OD

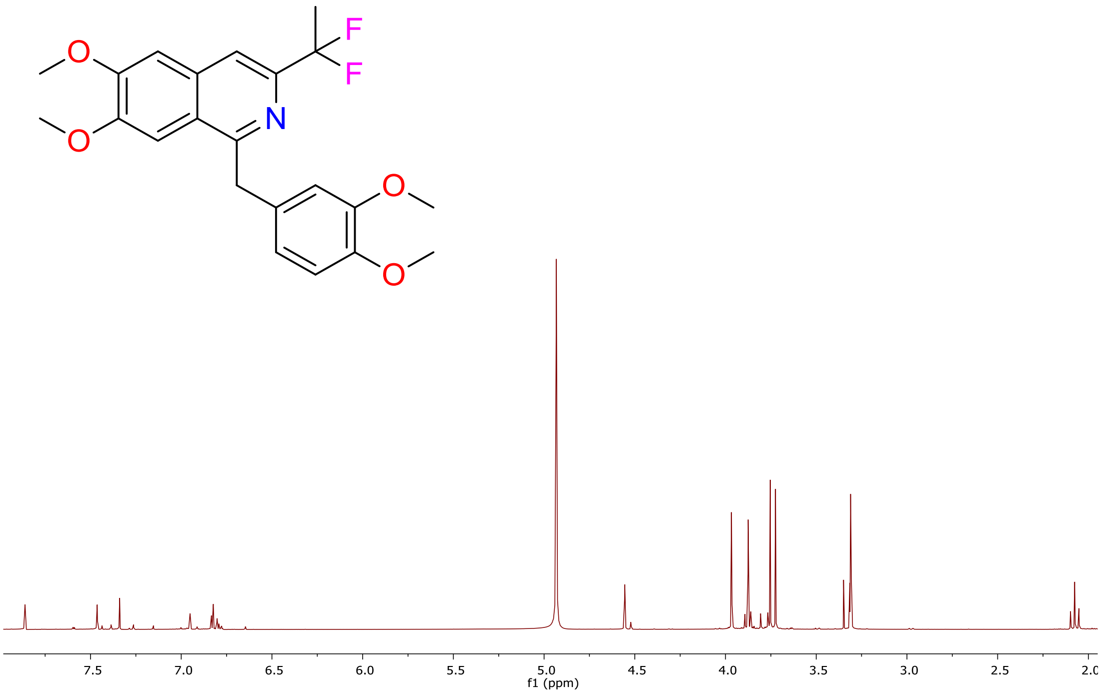

S27: ^{13}C NMR Spectrum for Compound **5** in CD_3OD

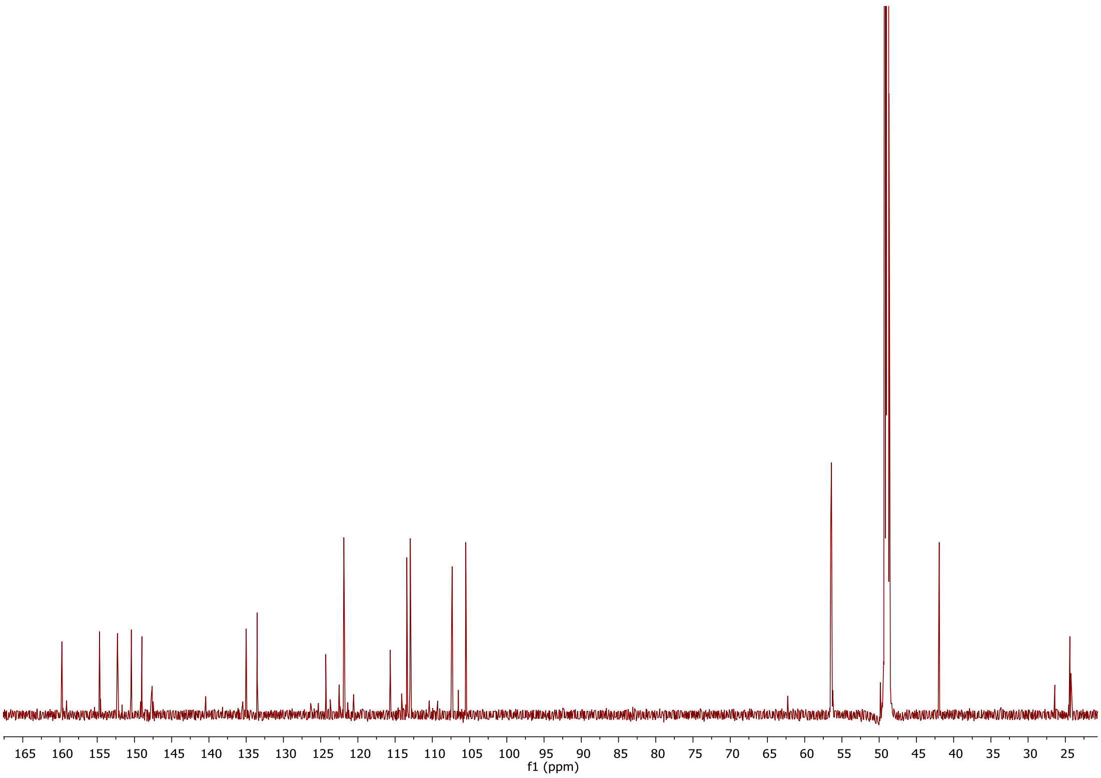

S28: COSY NMR Spectrum for Compound **5** in CD_3OD

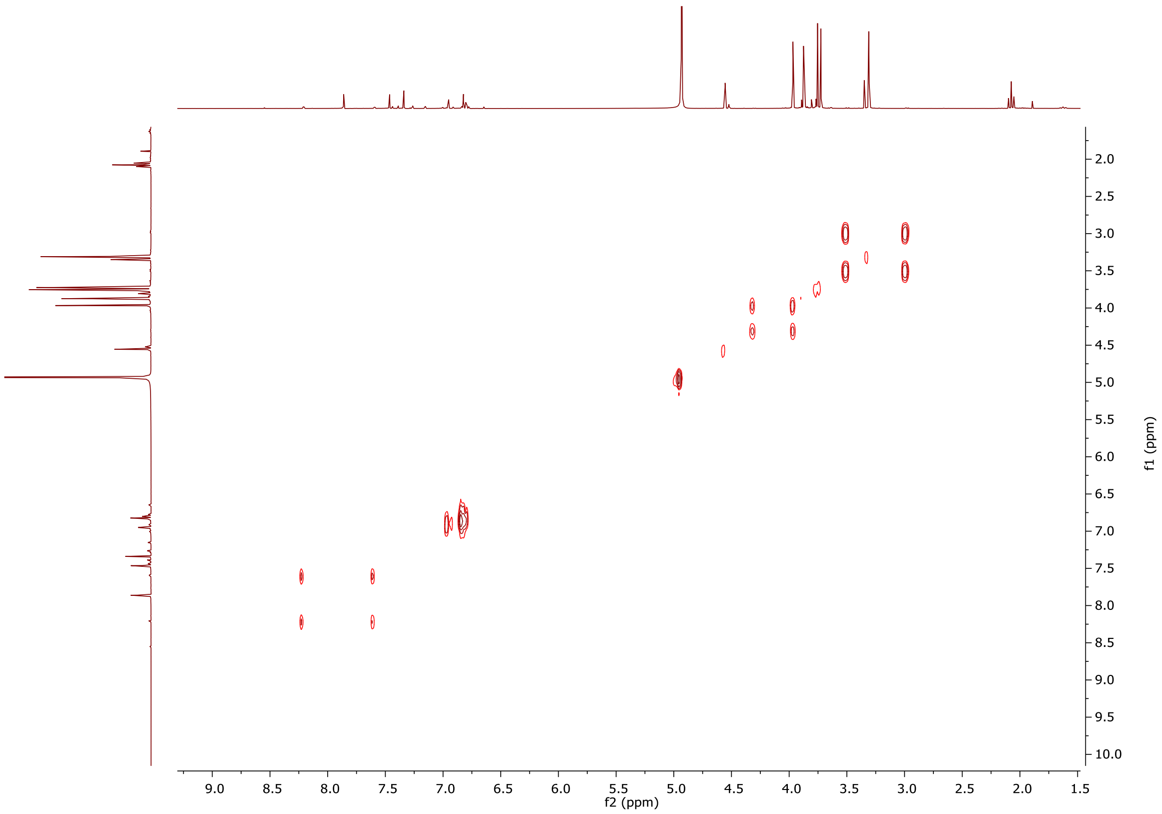

S29: HSQC NMR Spectrum for Compound **5** in CD_3OD

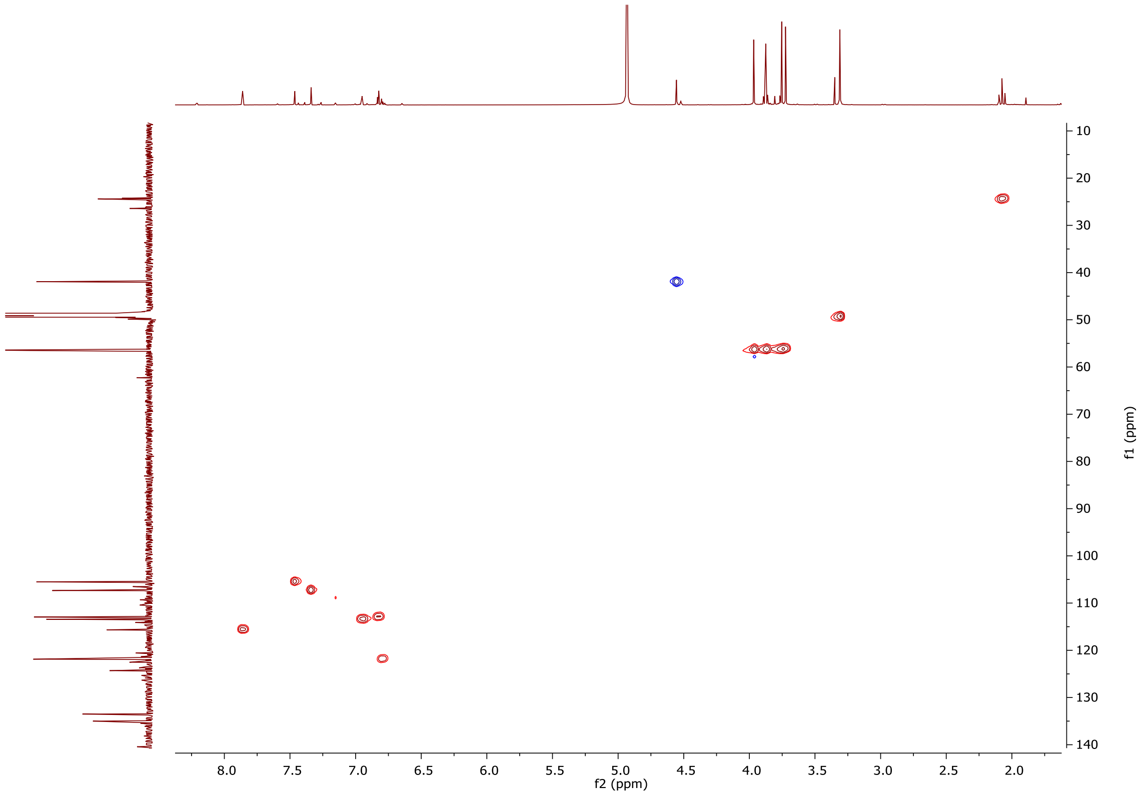

S30: HMBC NMR Spectrum for Compound **5** in CD_3OD

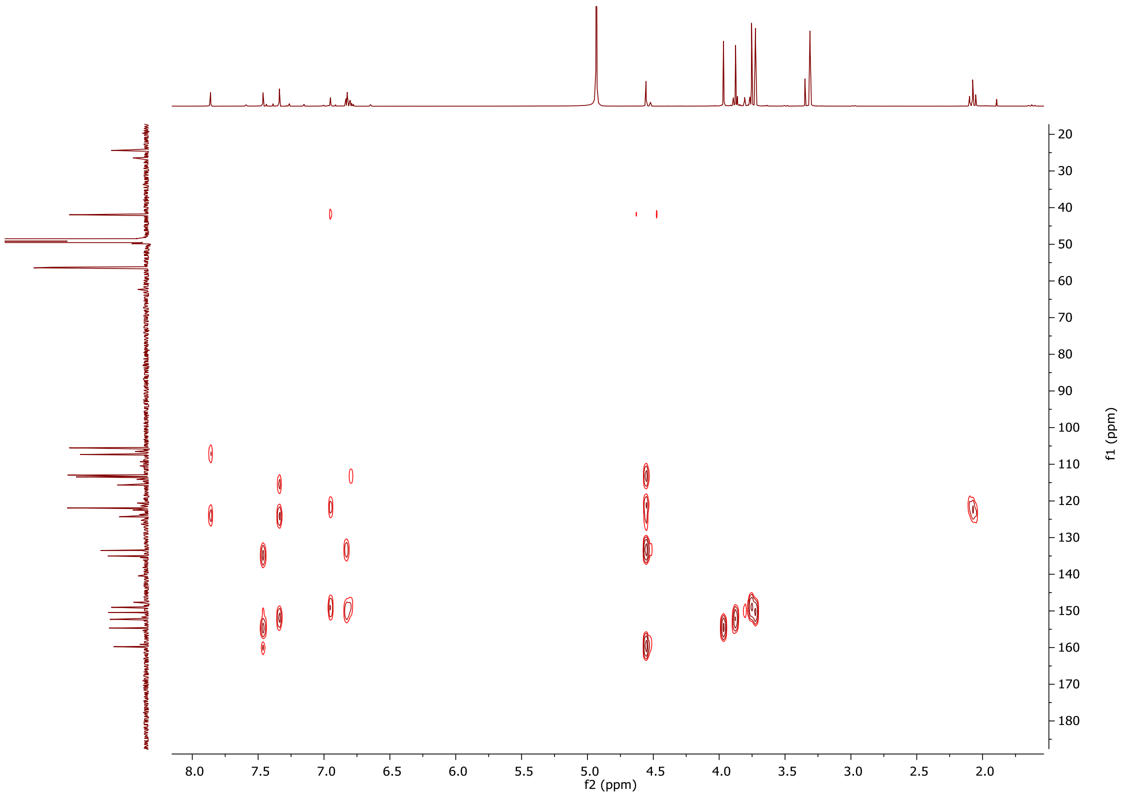

S31: ^{1}H NMR Spectrum for Compound **6** in CD_3OD

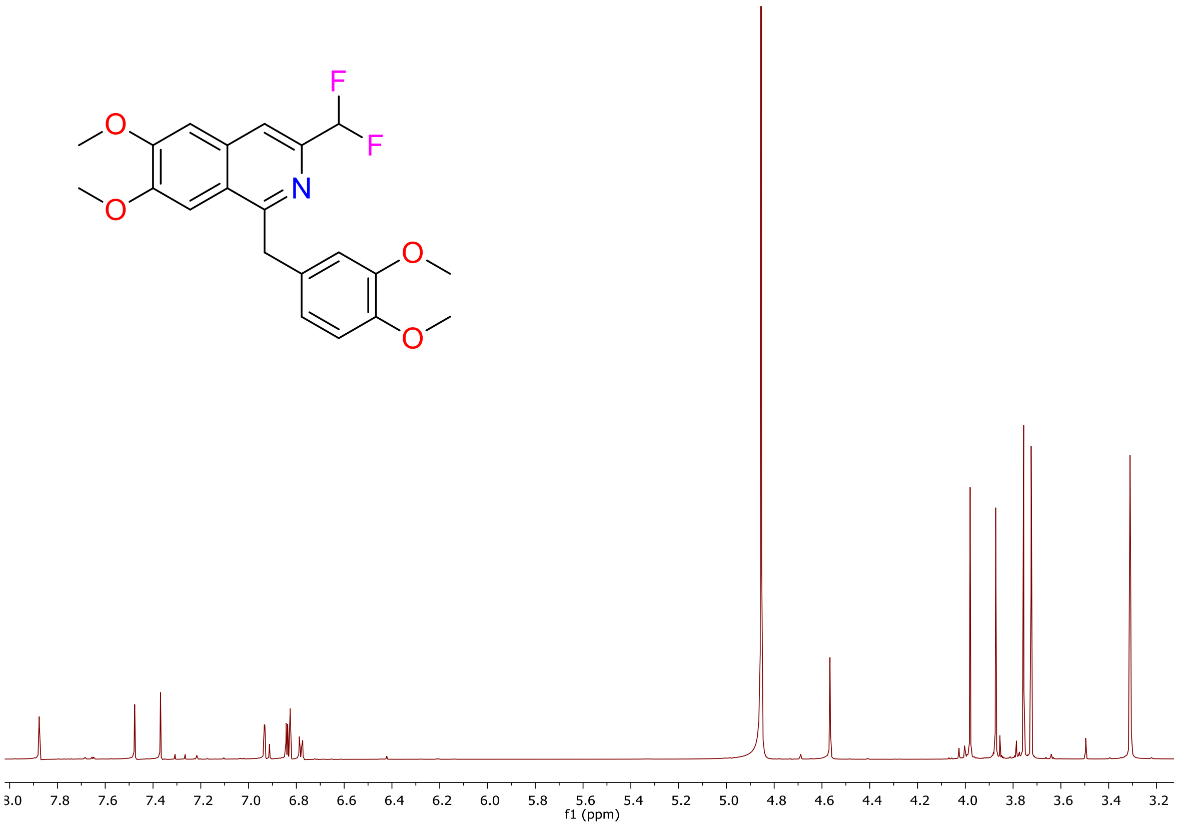

S32: ^{13}C NMR Spectrum for Compound **6** in CD_3OD

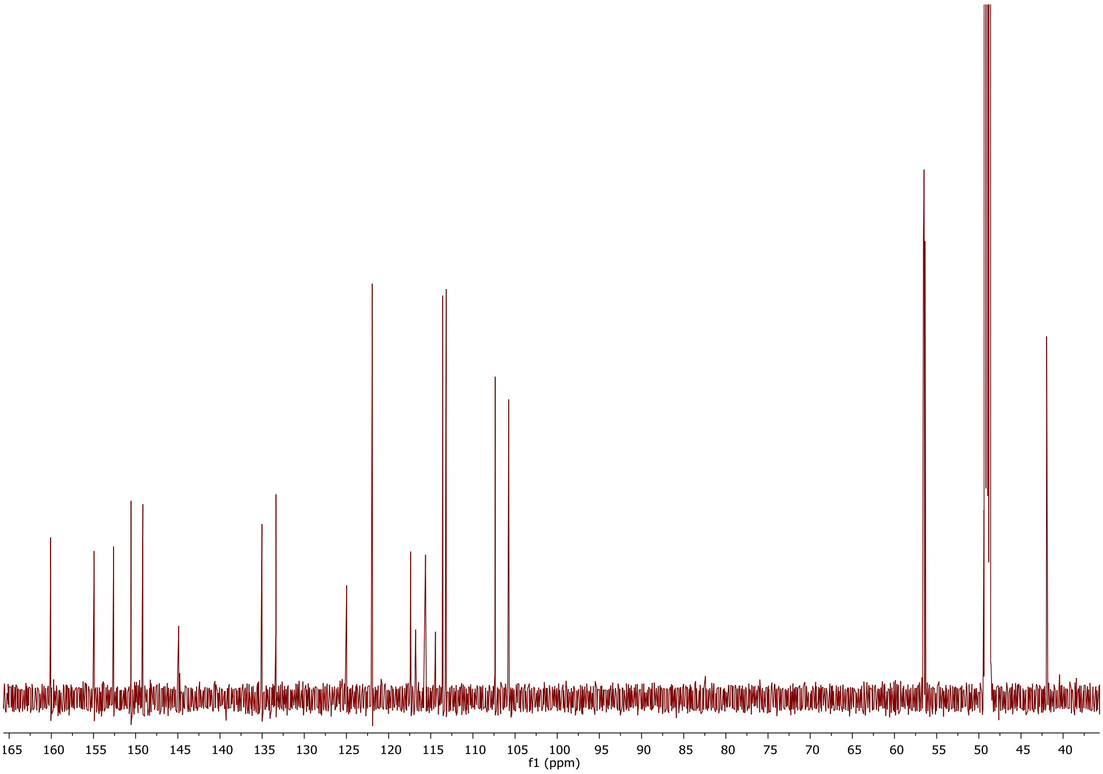

S33: COSY NMR Spectrum for Compound **6** in CD_3OD

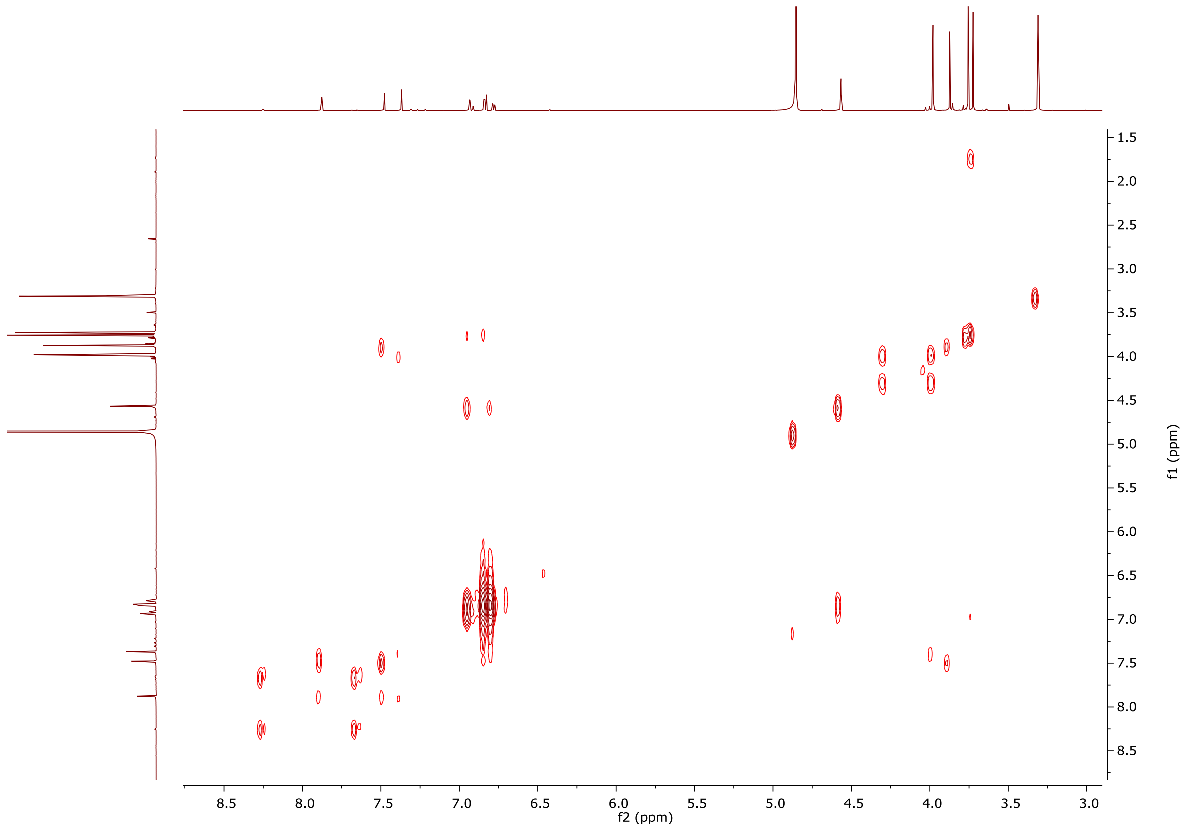

S34: HSQC NMR Spectrum for Compound **6** in CD_3OD

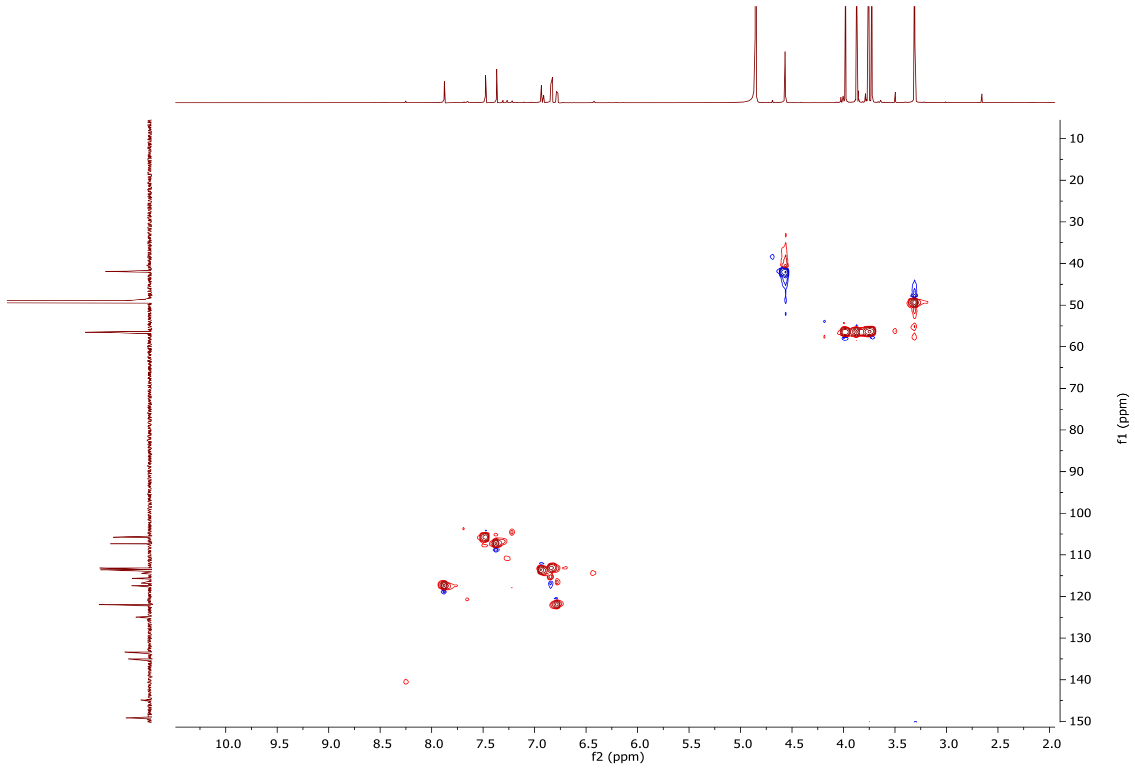

S35: HMBC NMR Spectrum for Compound **6** in CD_3OD

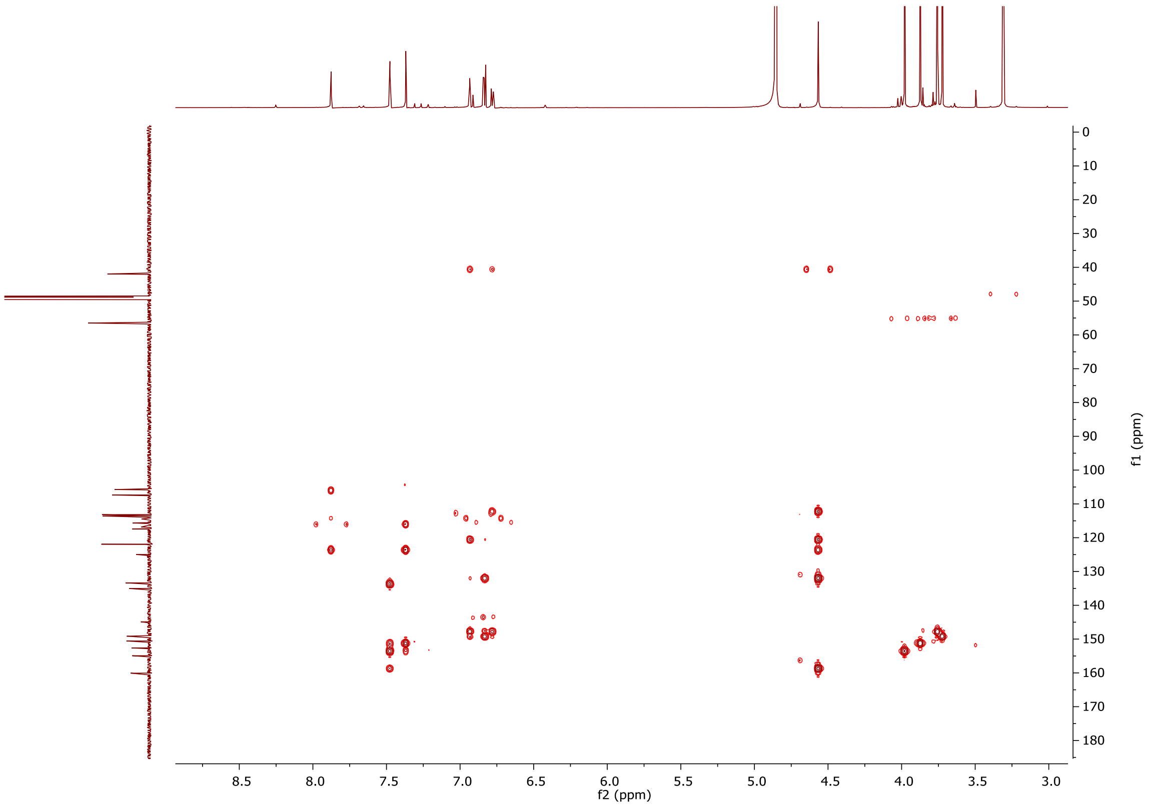

S36: ^{1}H NMR Spectrum for Compound **7** in CD_3OD

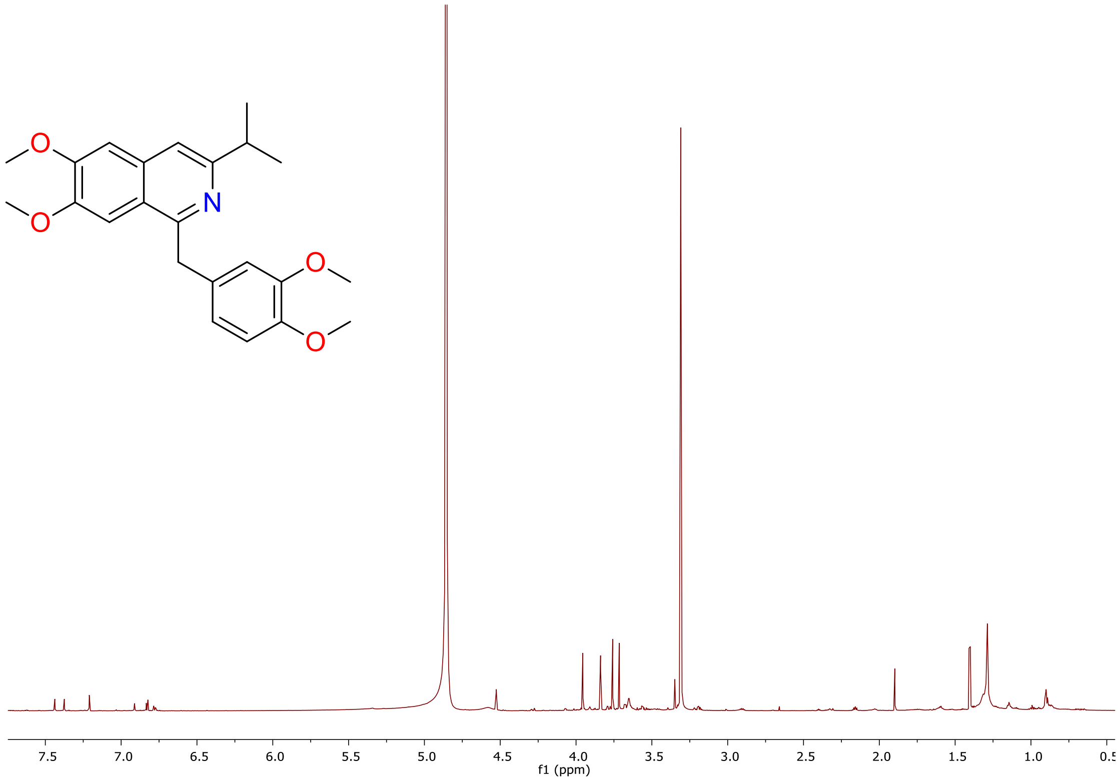

S37: ^{13}C NMR Spectrum for Compound **7** in CD_3OD

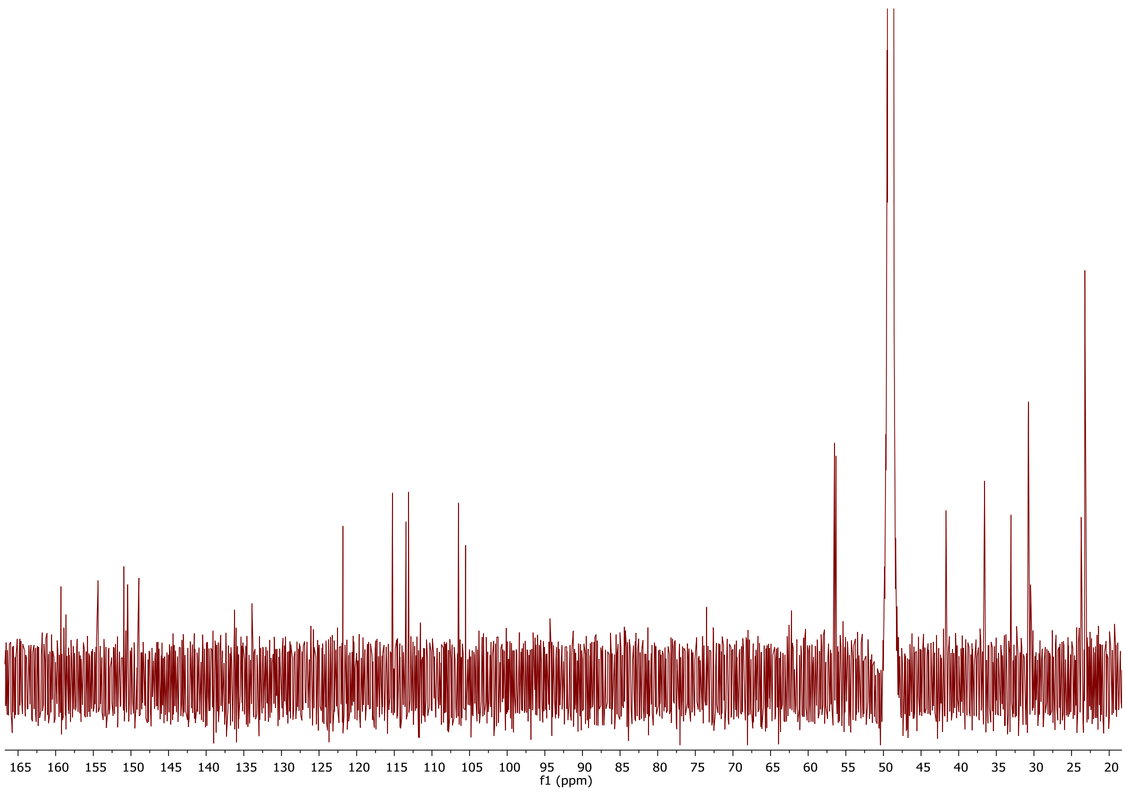

S38: COSY NMR Spectrum for Compound **7** in CD_3OD

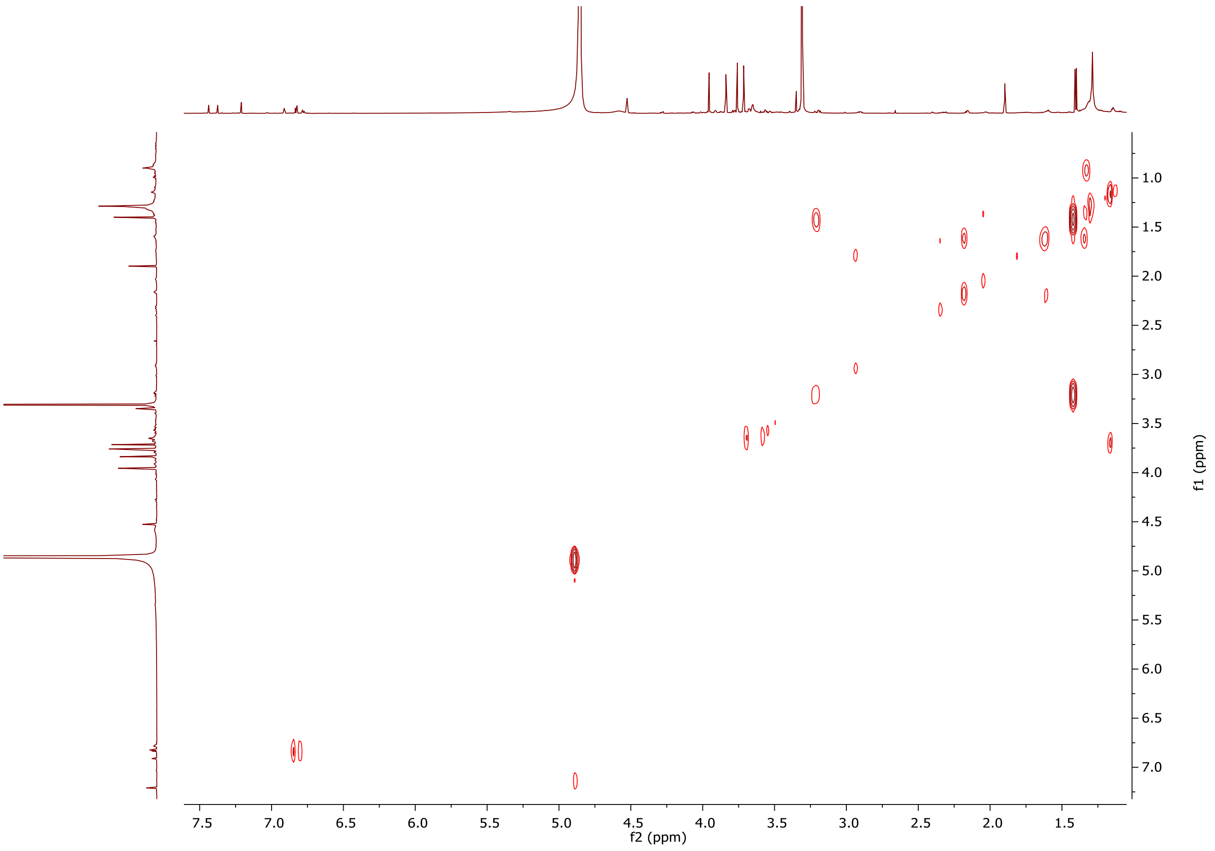

S39: HSQC NMR Spectrum for Compound **7** in CD_3OD

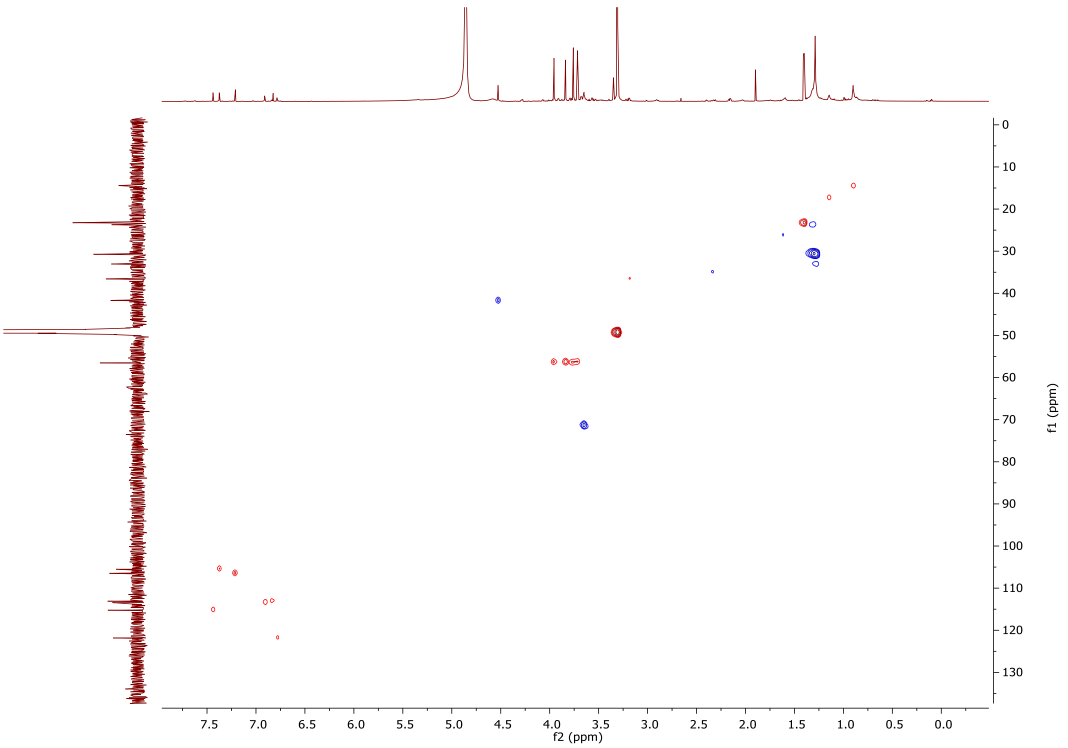

S40: HMBC NMR Spectrum for Compound **7** in CD_3OD

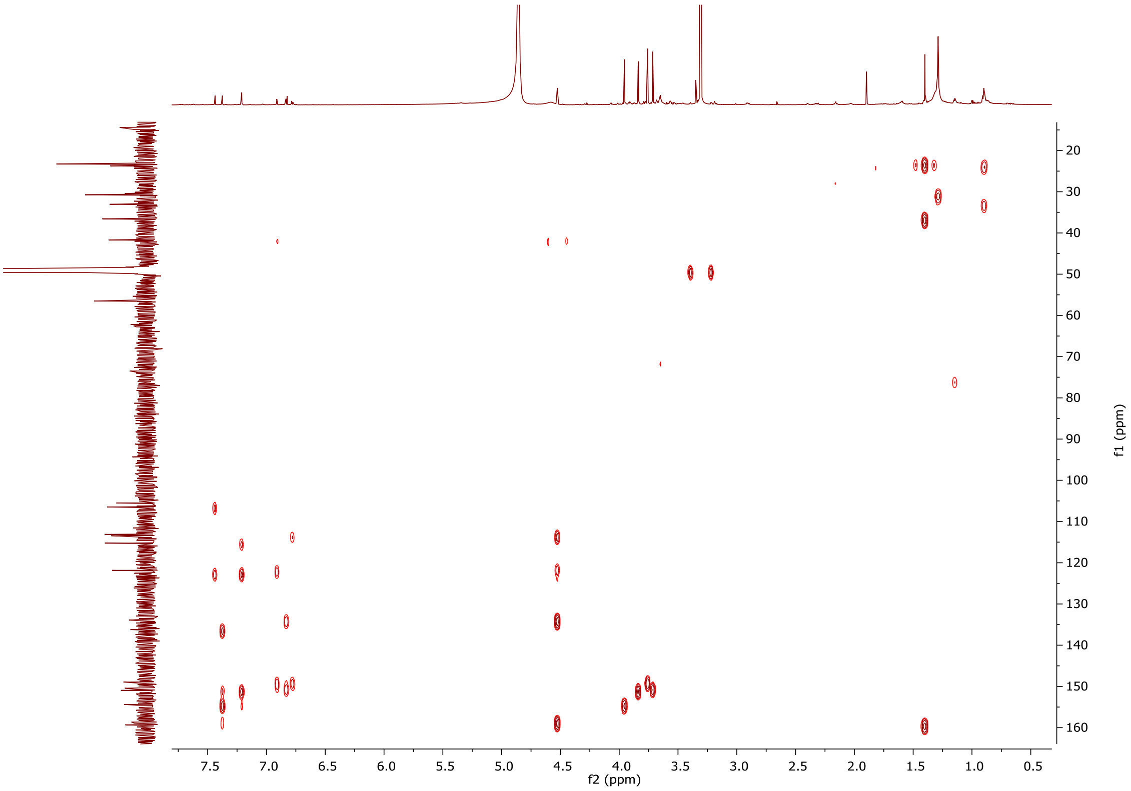

S41: ^{1}H NMR Spectrum for Compound **8** in CD_3OD

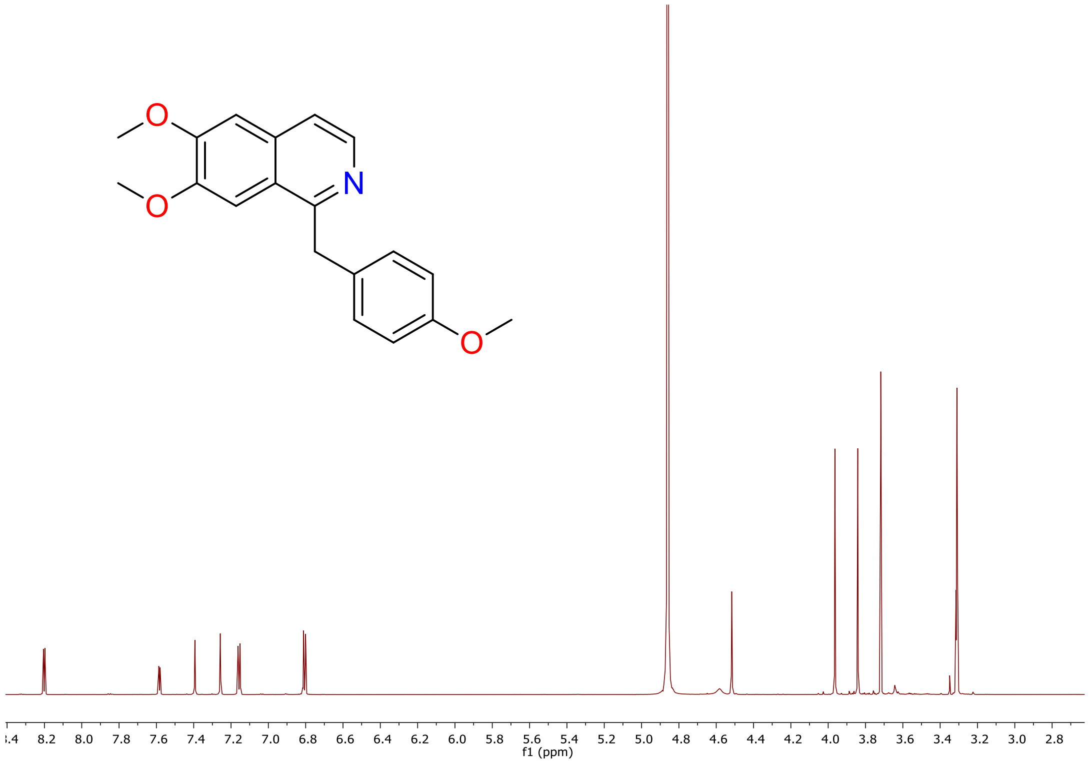

S42: ^{13}C NMR Spectrum for Compound **8** in CD_3OD

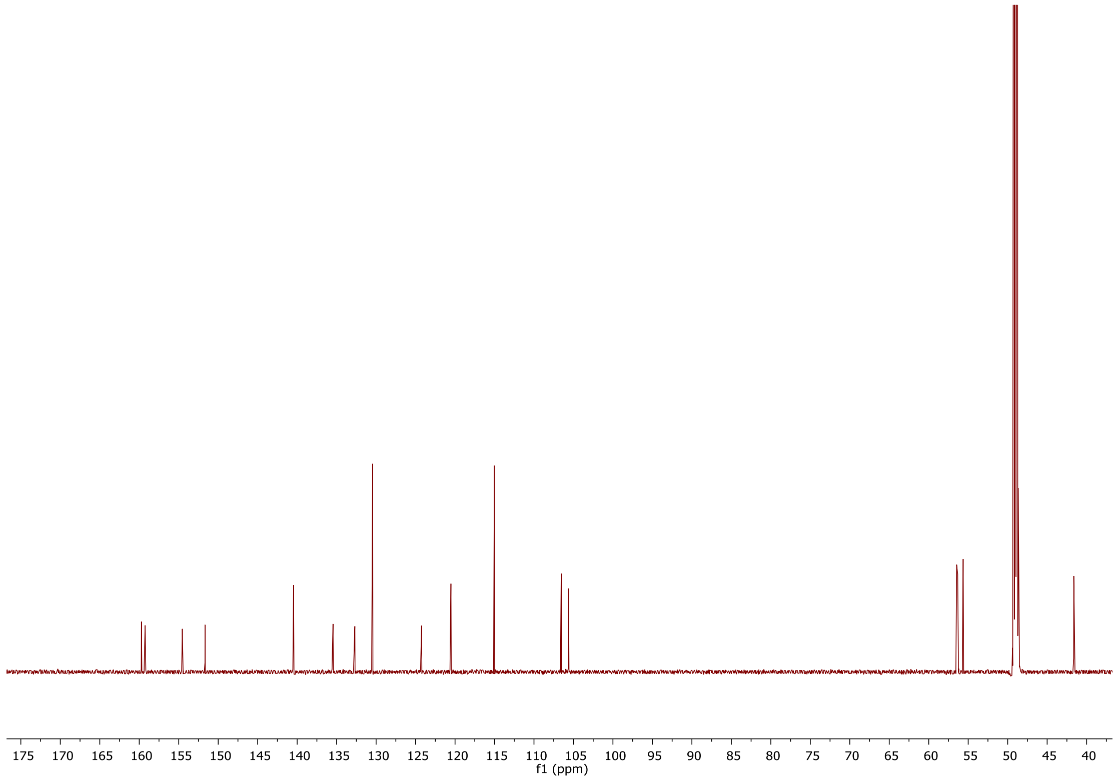

S43: COSY NMR Spectrum for Compound **8** in CD_3OD

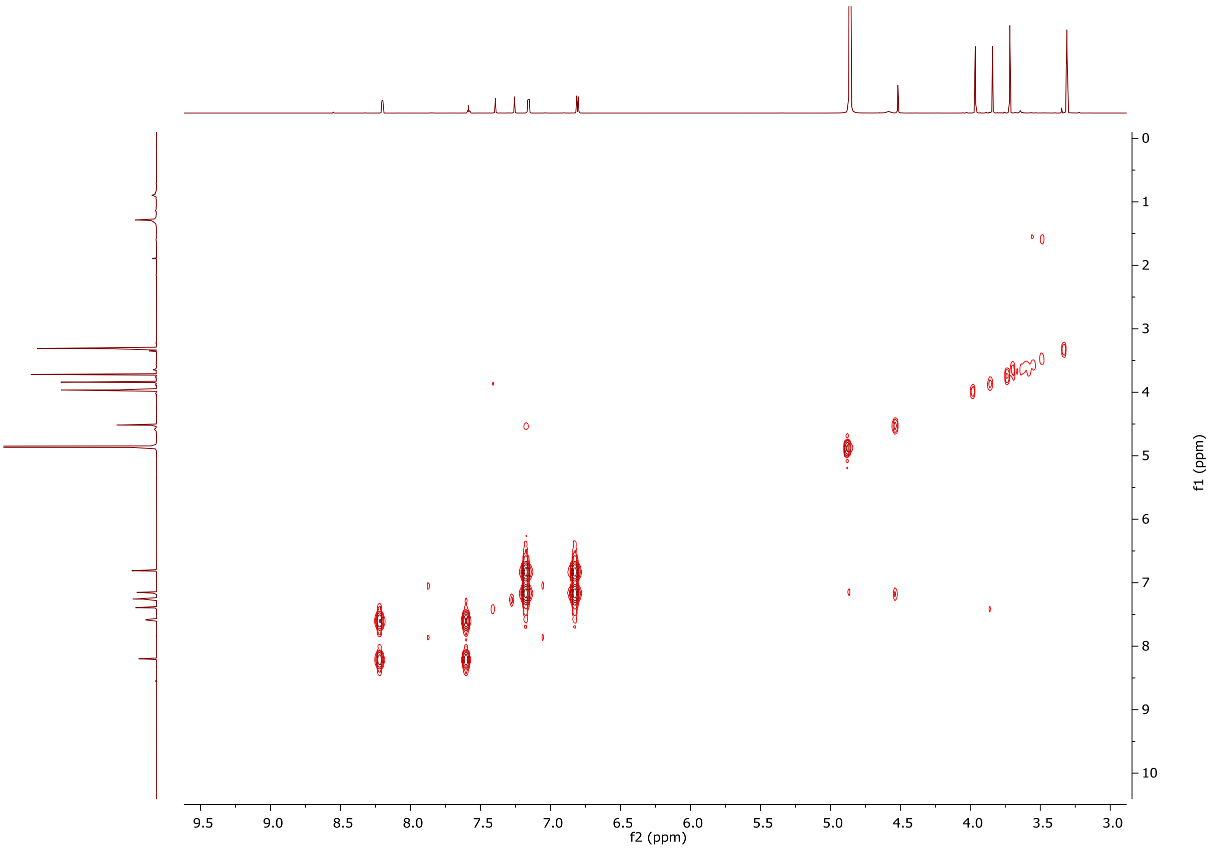

S44: HSQC NMR Spectrum for Compound **8** in CD_3OD

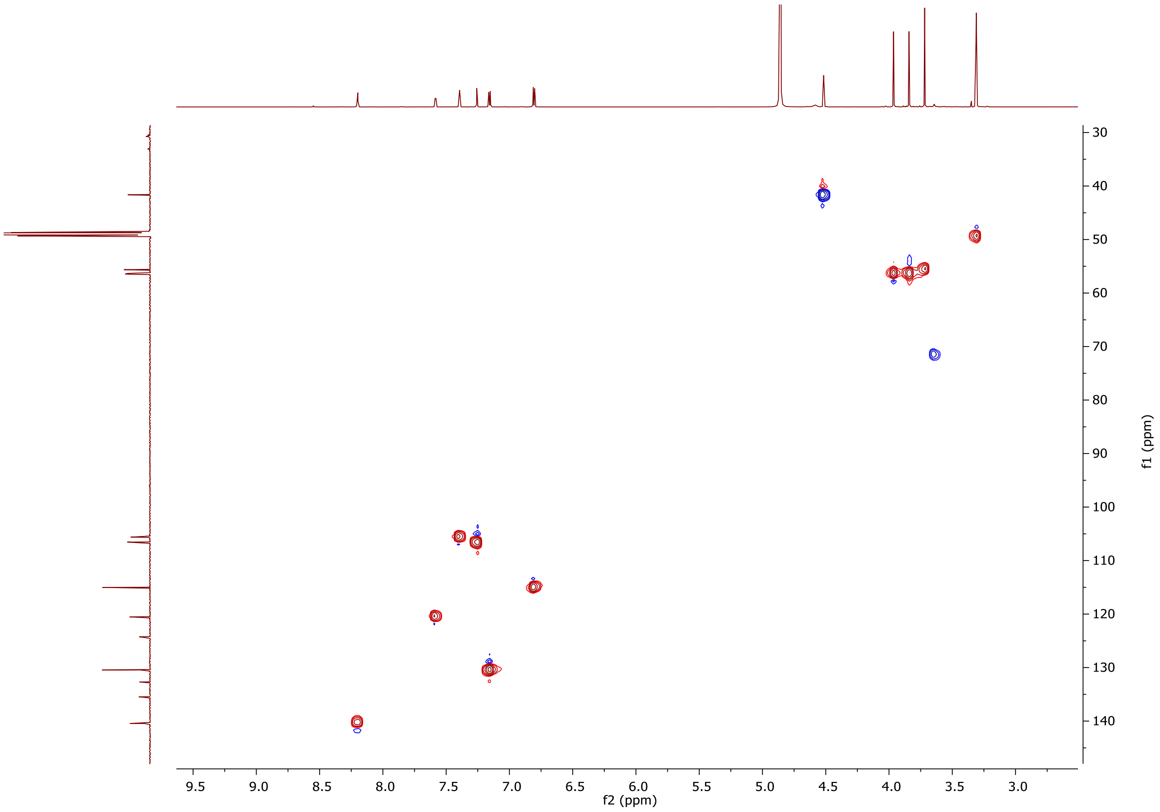

S45: HMBC NMR Spectrum for Compound **8** in CD_3OD

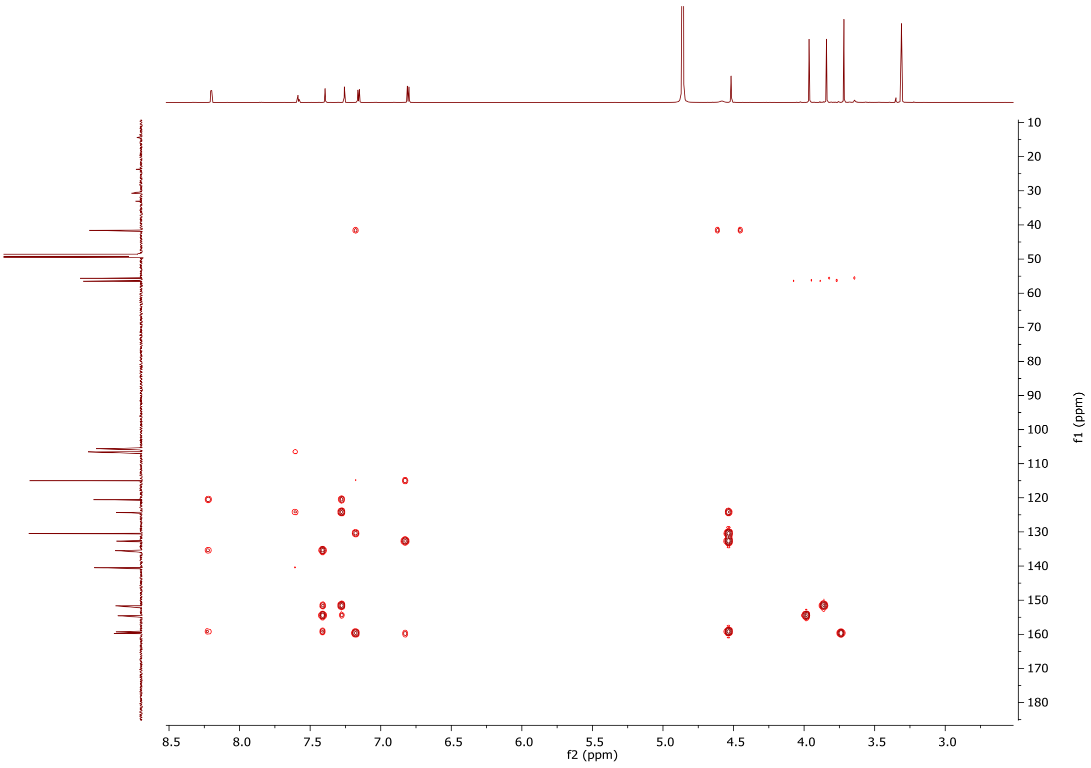

S46: ^{1}H NMR Spectrum for Compound **9** in CD_3OD

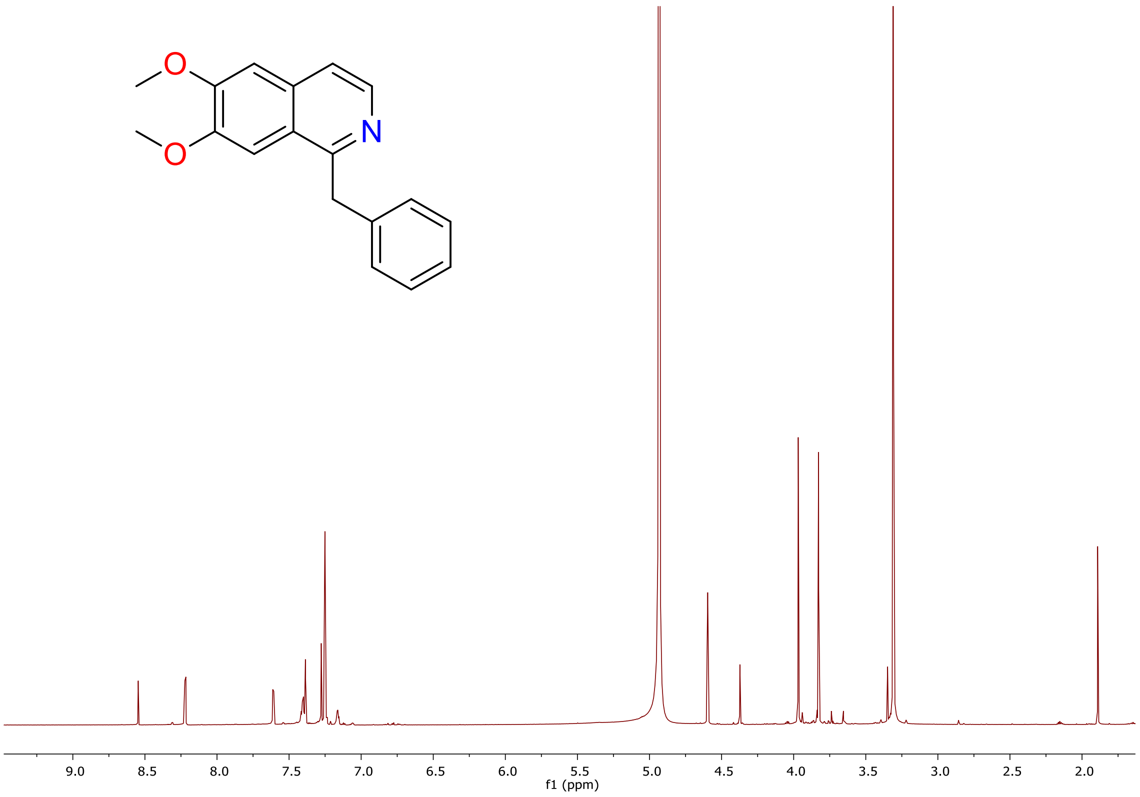

S47: ^{13}C NMR Spectrum for Compound **9** in CD_3OD

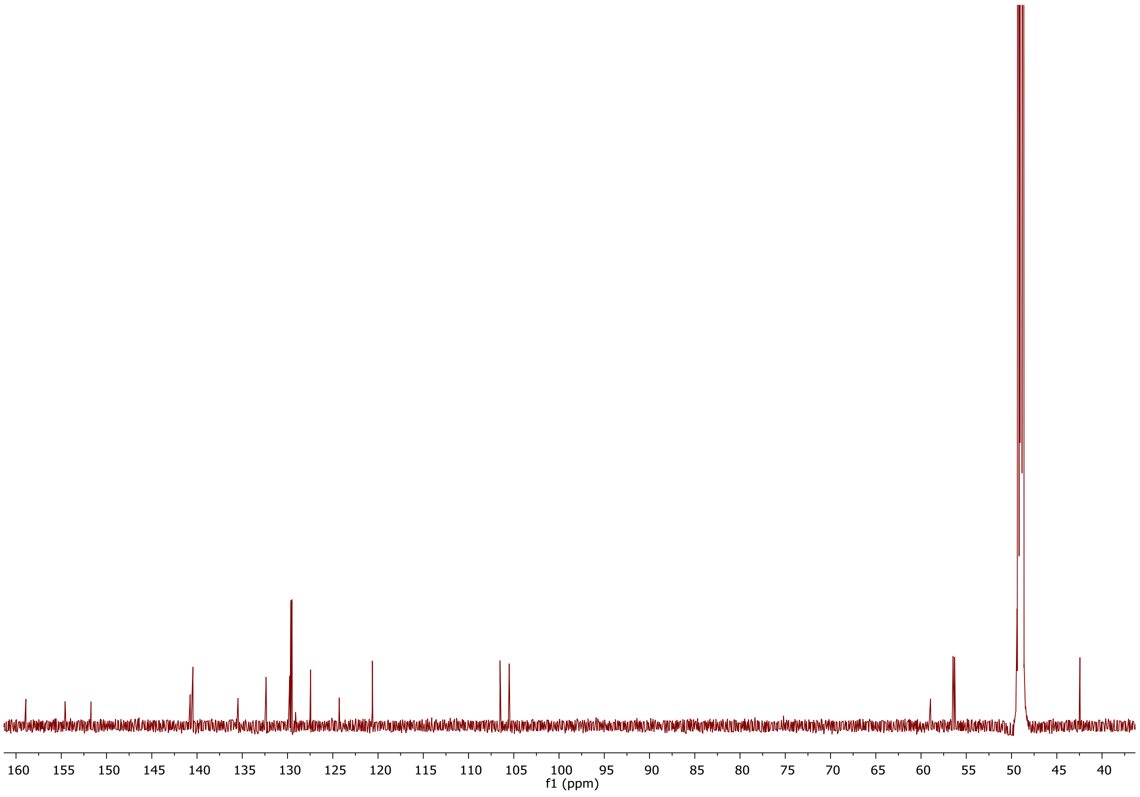

S48: COSY NMR Spectrum for Compound **9** in CD_3OD

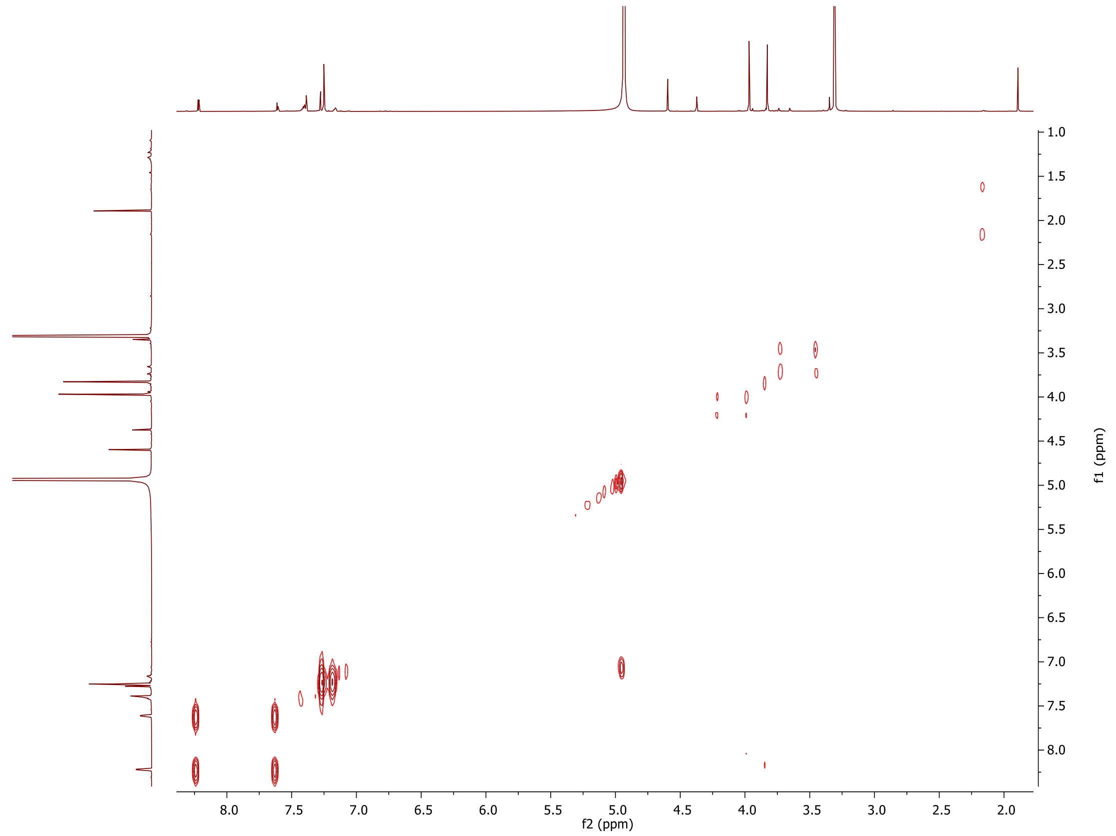

S49: HSQC NMR Spectrum for Compound **9** in CD_3OD

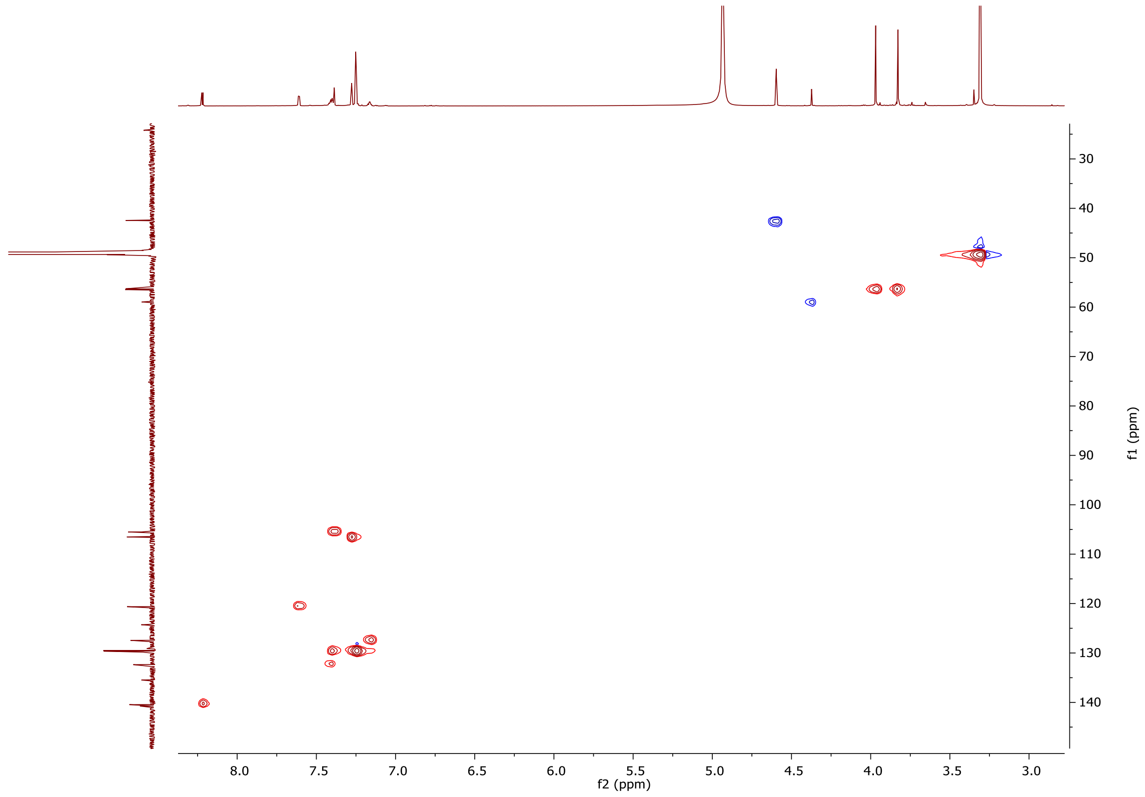

S50: HMBC NMR Spectrum for Compound **9** in CD_3OD

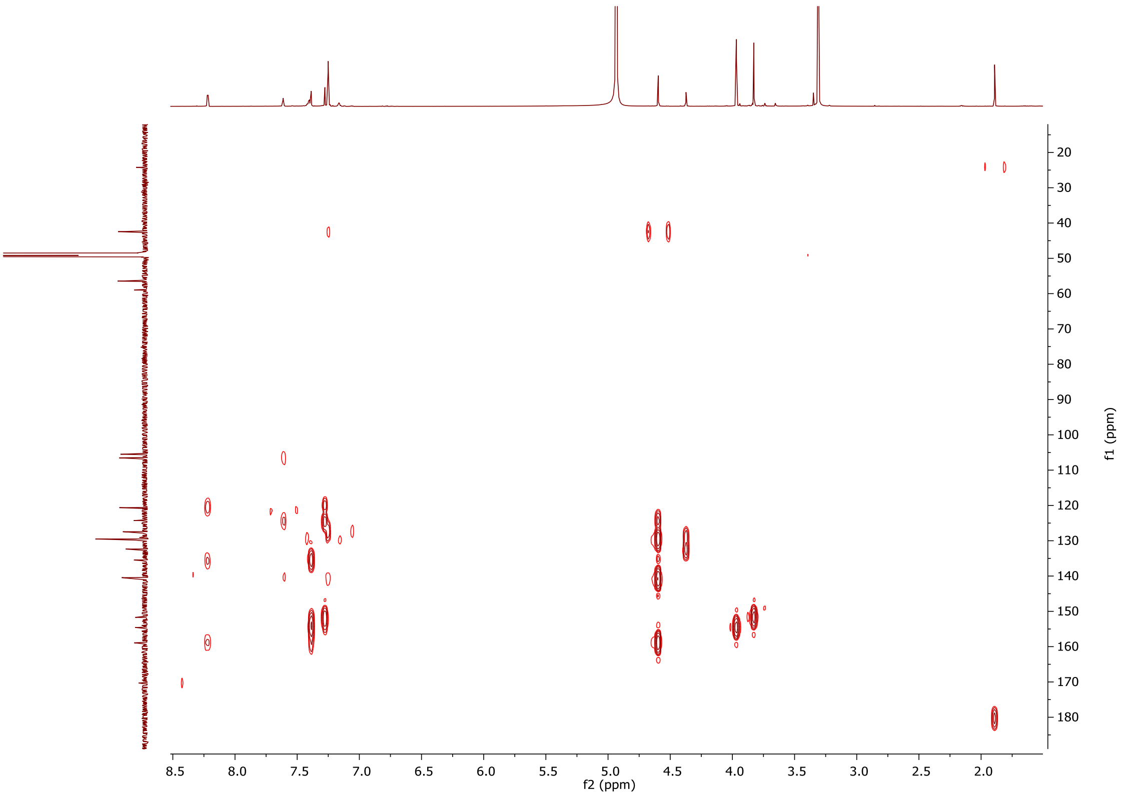

S51: ^{1}H NMR Spectrum for Compound **10** in CD_3OD

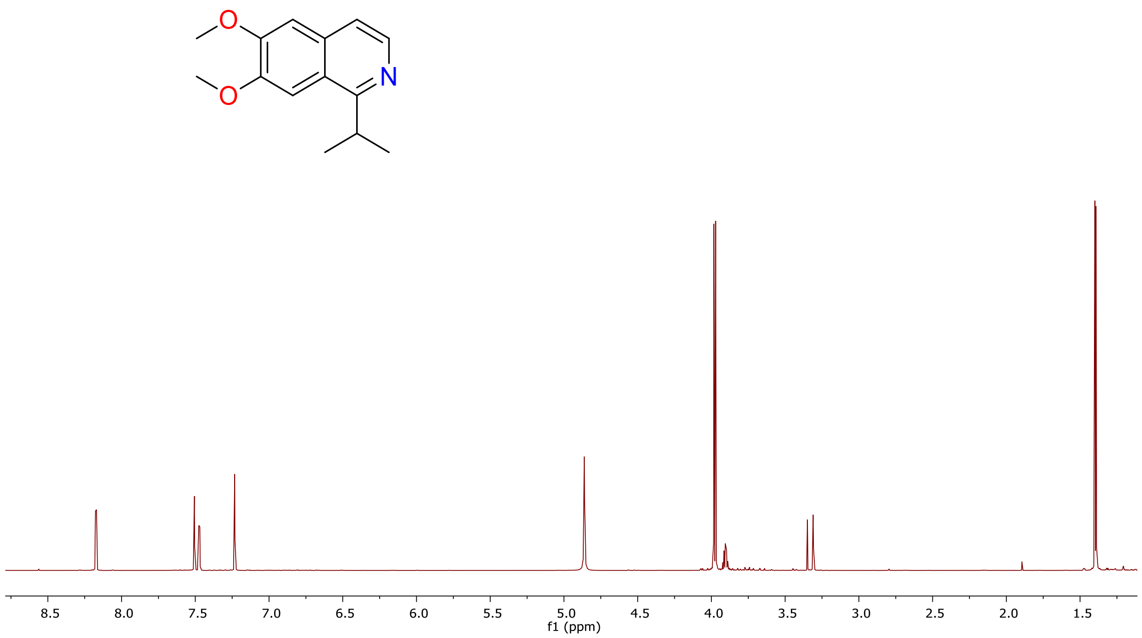

S52: ^{13}C NMR Spectrum for Compound **10** in CD_3OD

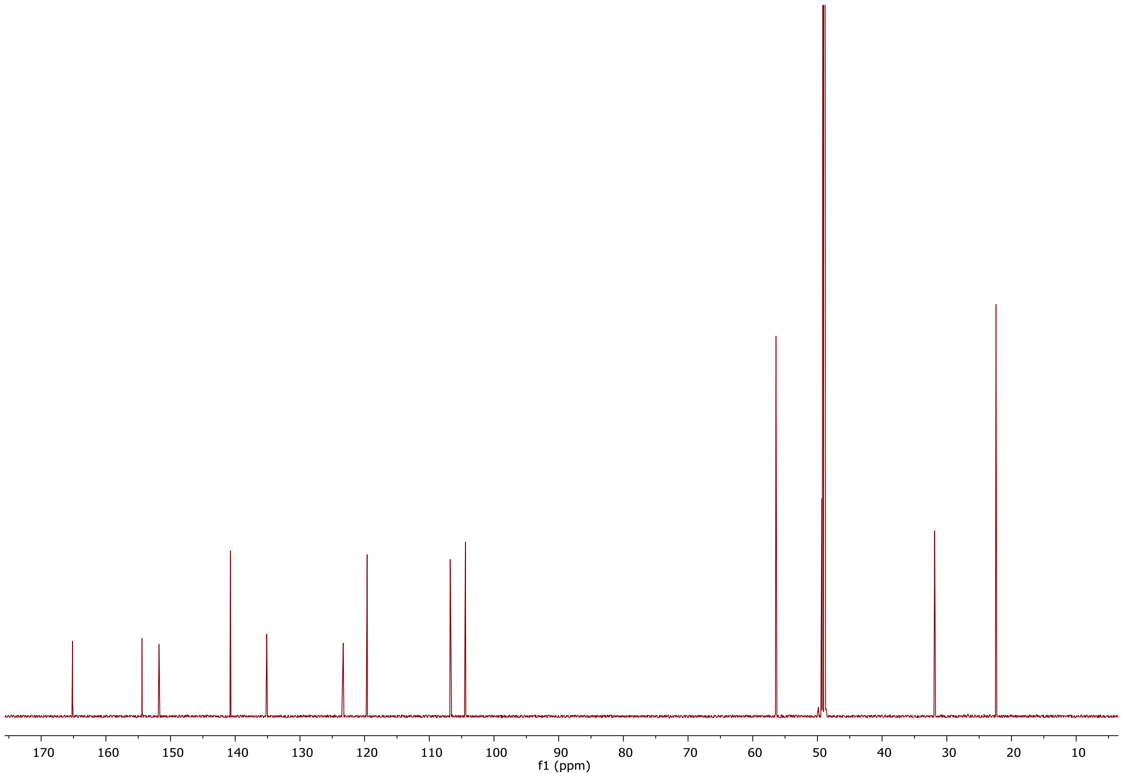

S53: COSY NMR Spectrum for Compound **10** in CD_3OD

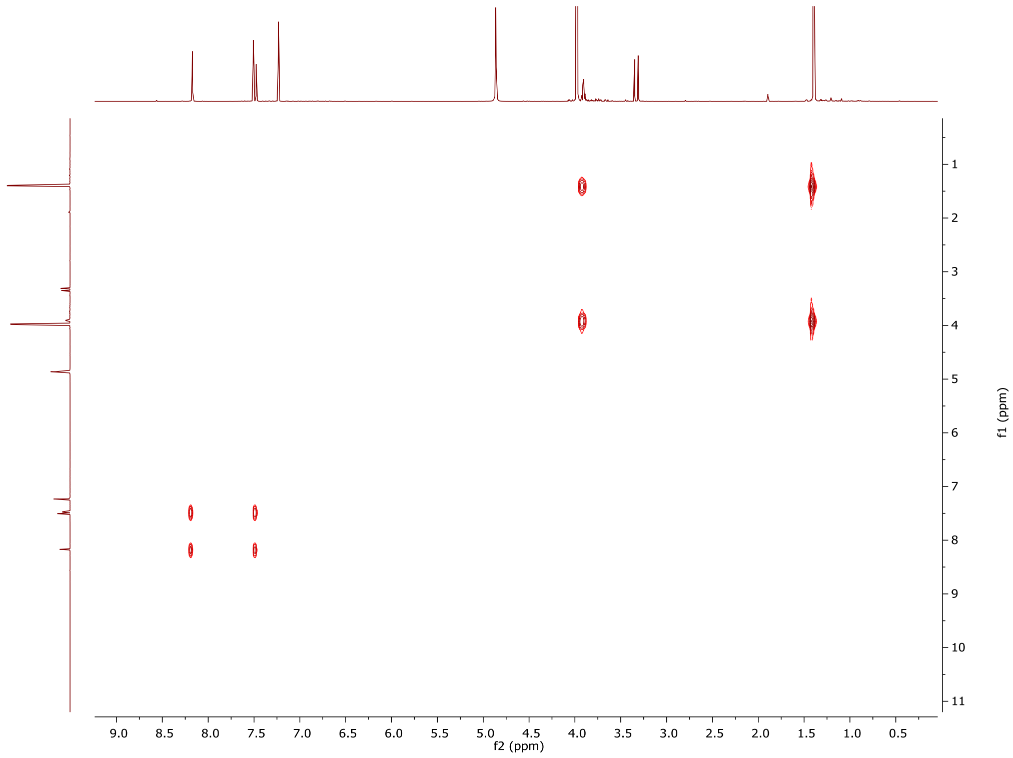

S54: HSQC NMR Spectrum for Compound **10** in CD_3OD

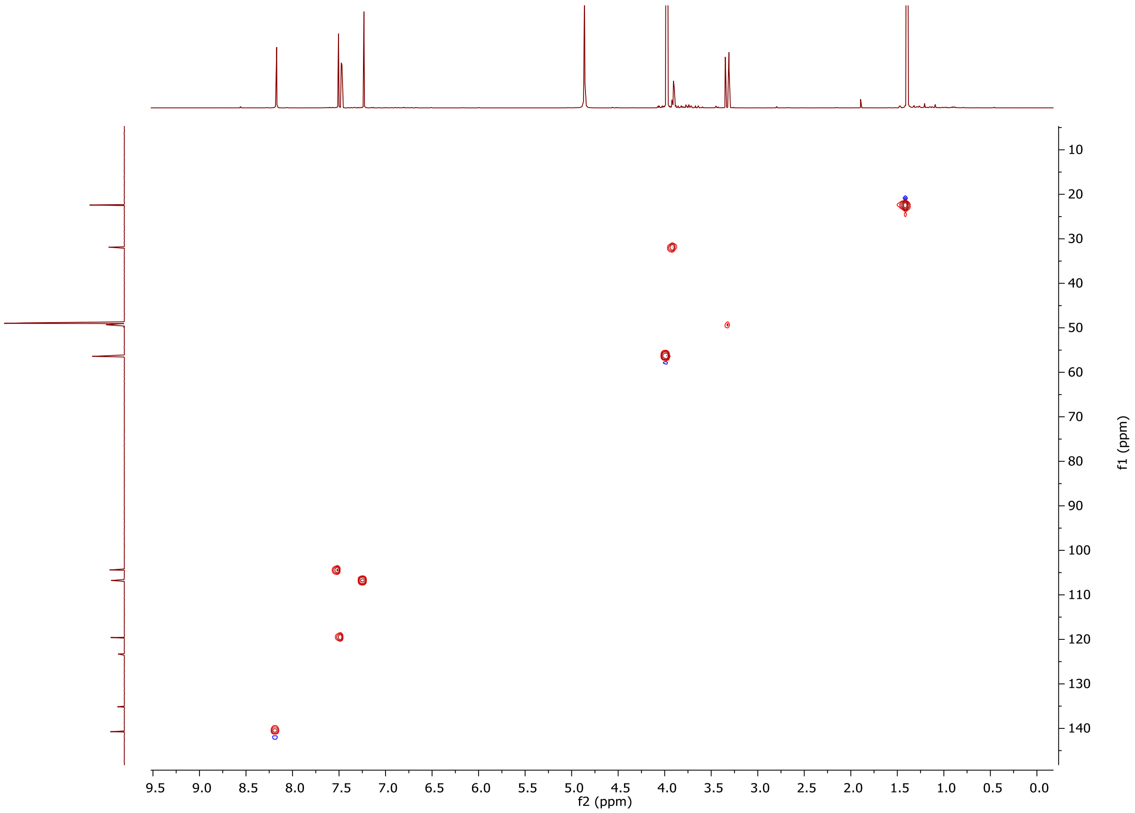

S55: HMBC NMR Spectrum for Compound **10** in CD_3OD

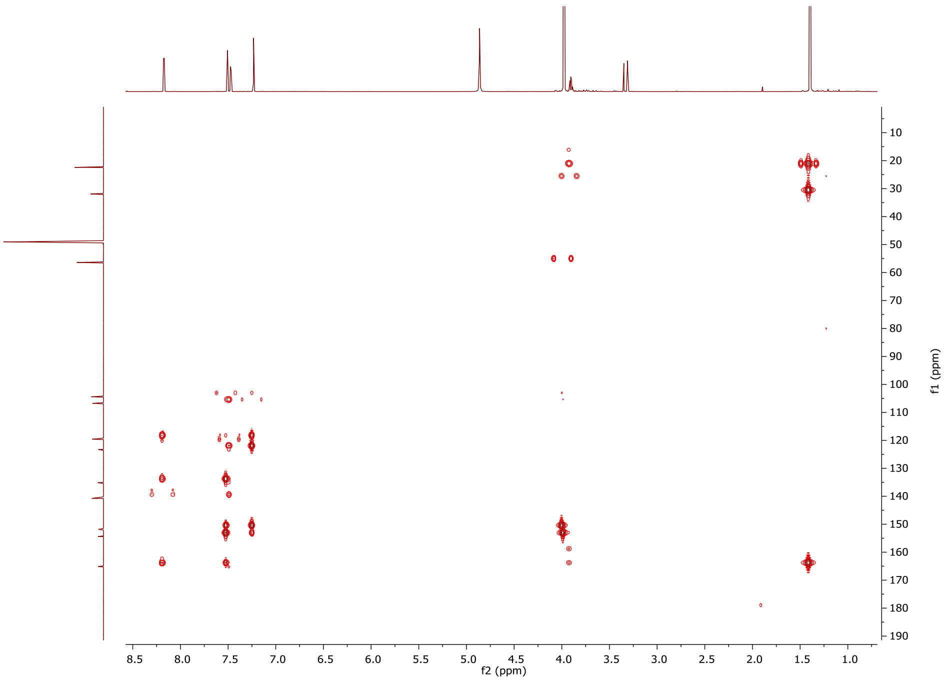

S56: ^{1}H NMR Spectrum for Compound **11** in CD_3OD

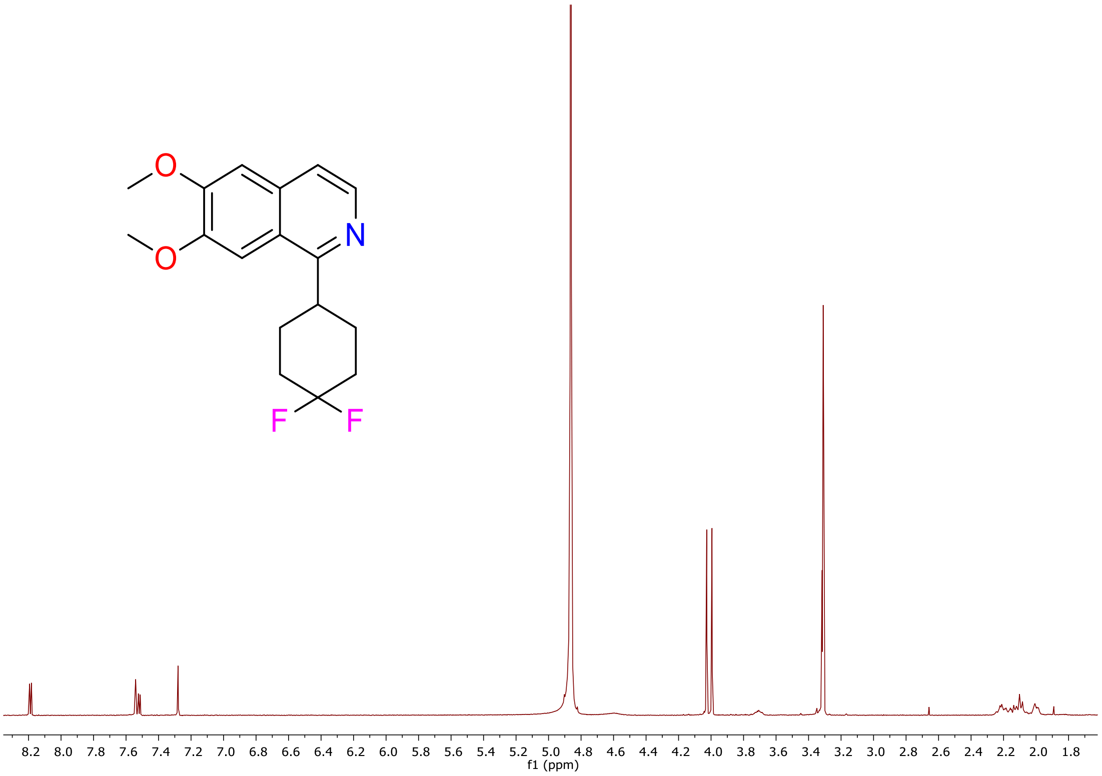

S57: ^{13}C NMR Spectrum for Compound **11** in CD_3OD

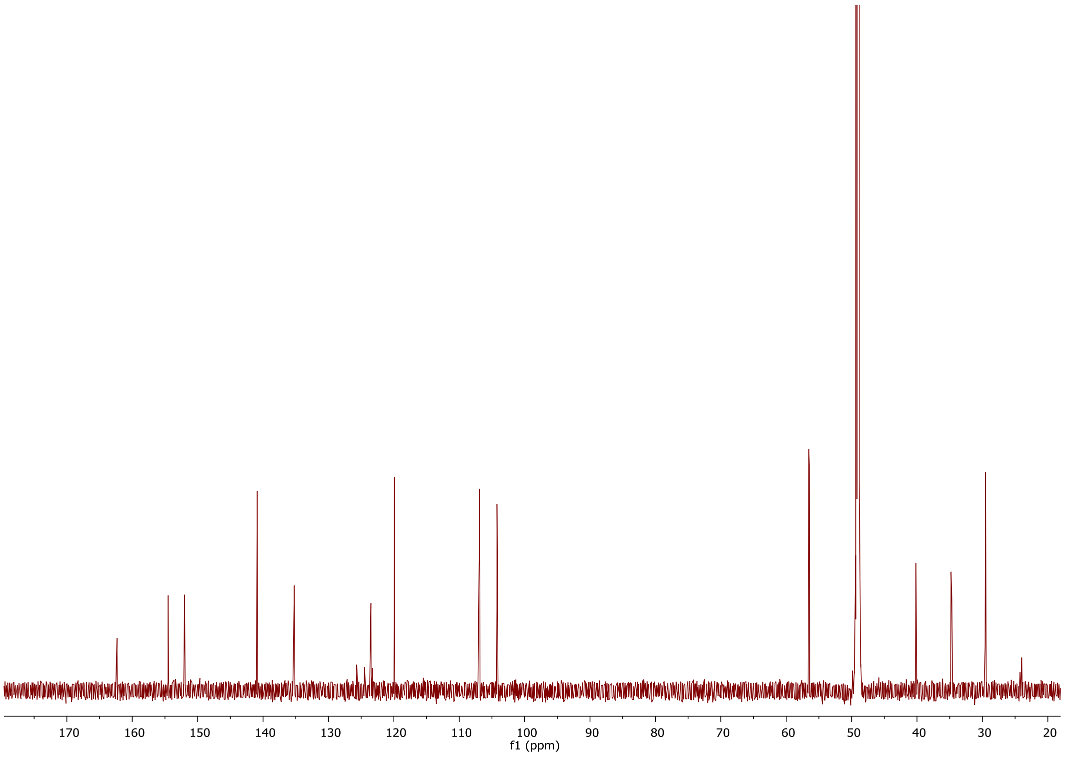

S58: COSY NMR Spectrum for Compound **11** in CD_3OD

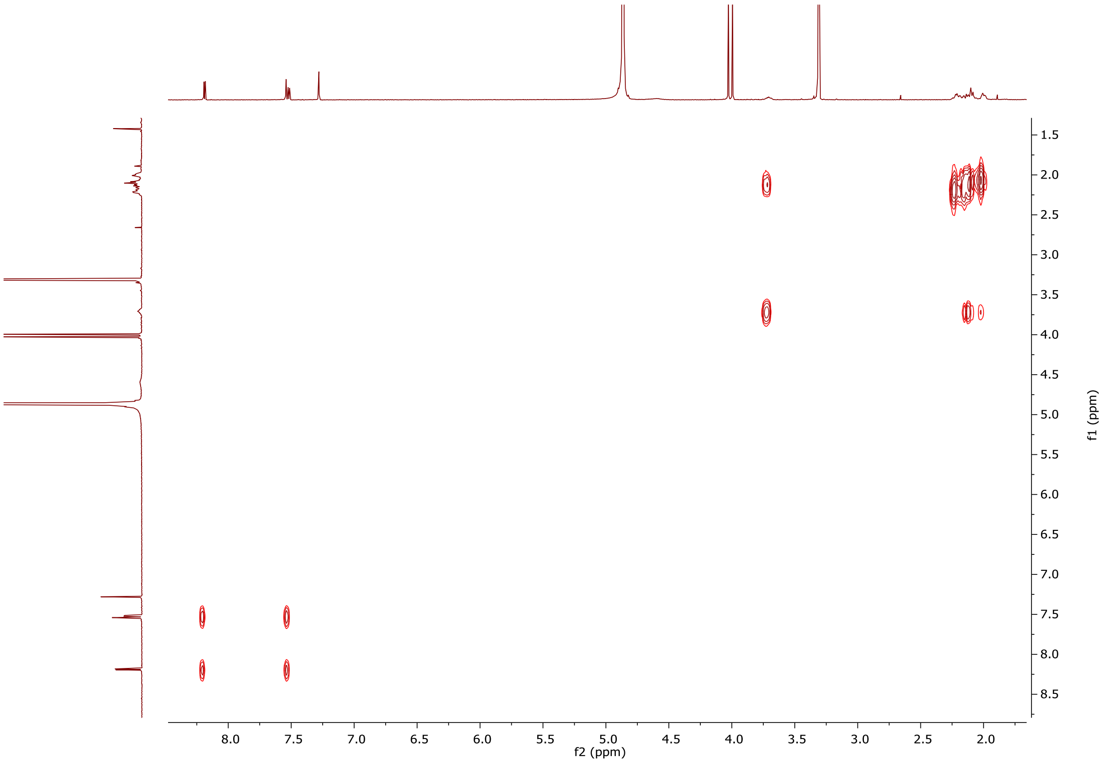

S59: HSQC NMR Spectrum for Compound **11** in CD_3OD

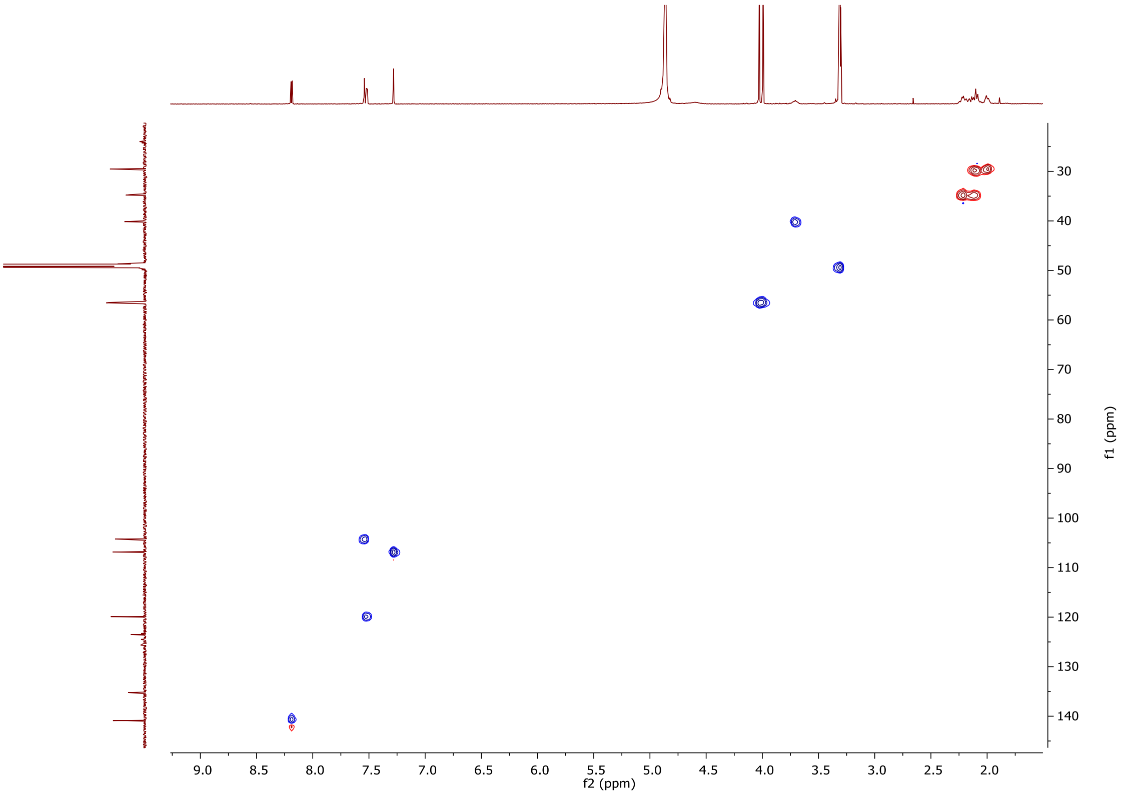

S60: HMBC NMR Spectrum for Compound **11** in CD_3OD

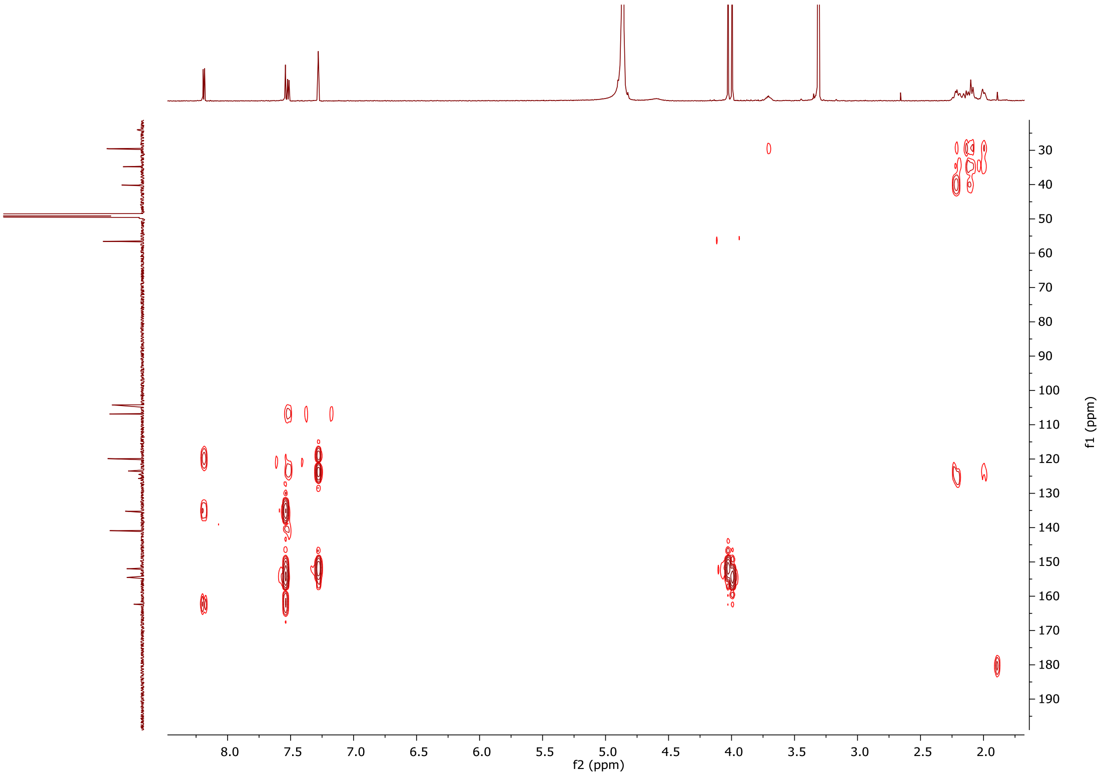

S61: ^{1}H NMR Spectrum for Compound **12** in $CDCl_3$

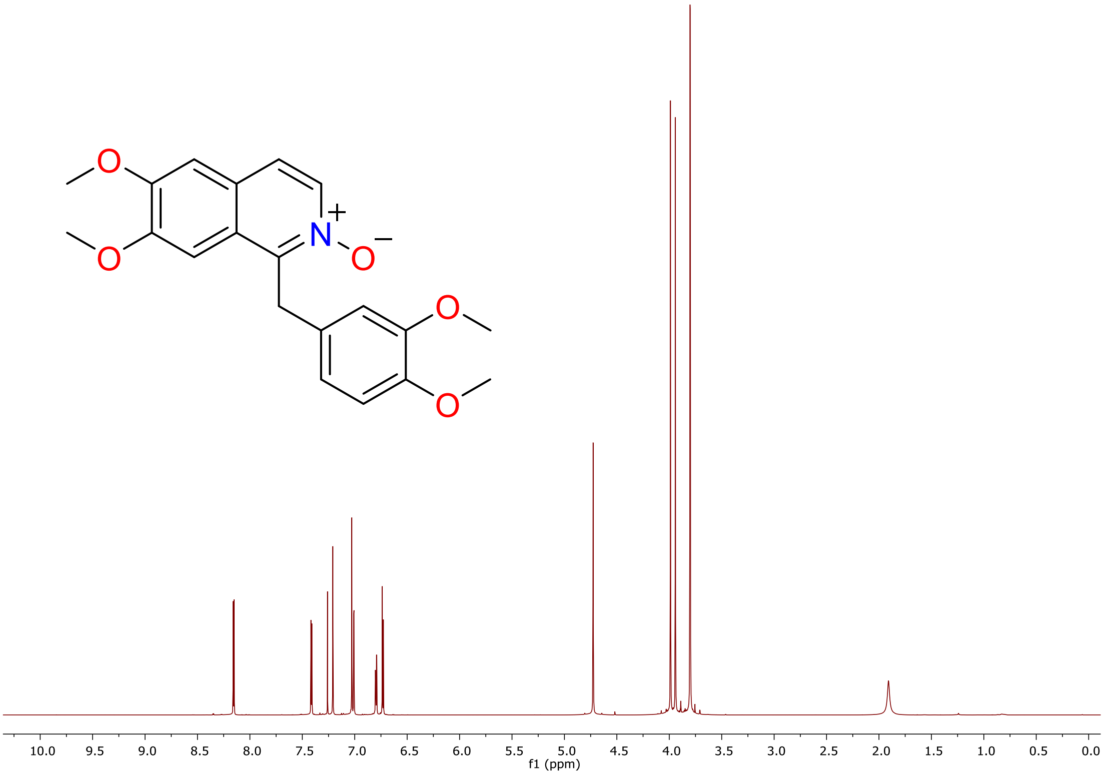

S62: ^{13}C NMR Spectrum for Compound **12** in $CDCl_3$

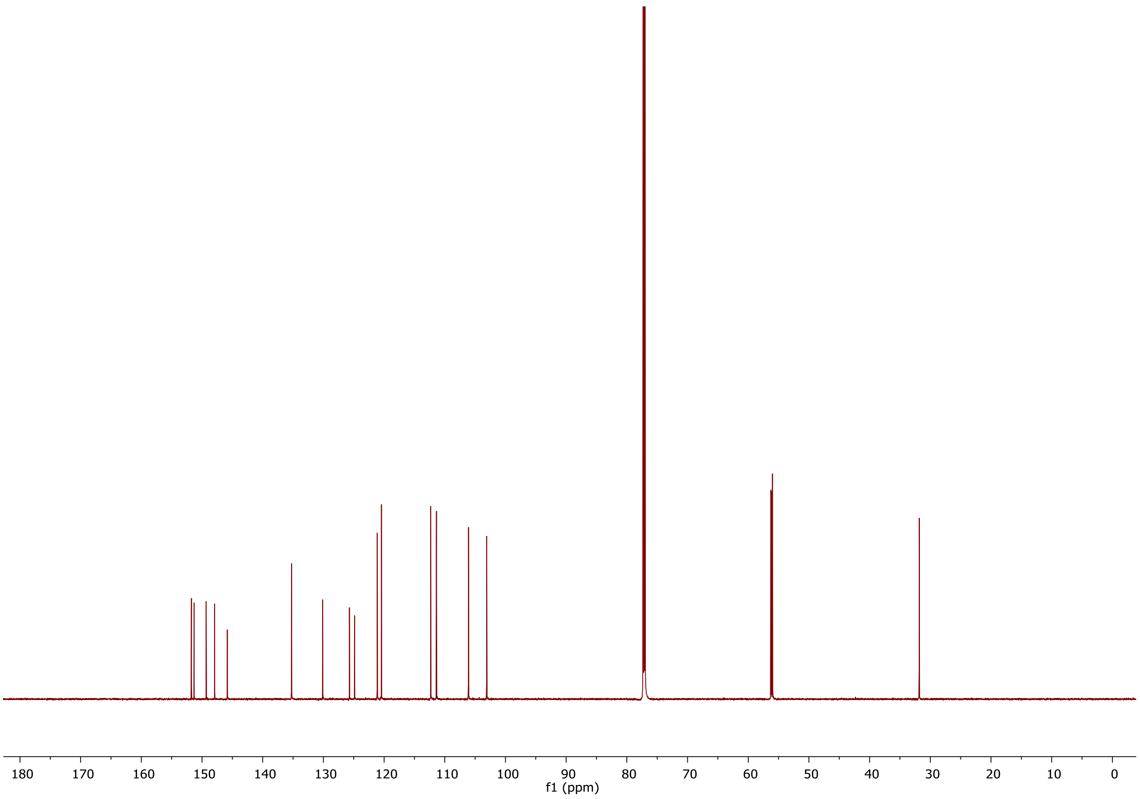

S63: COSY NMR Spectrum for Compound **12** in $CDCl_3$

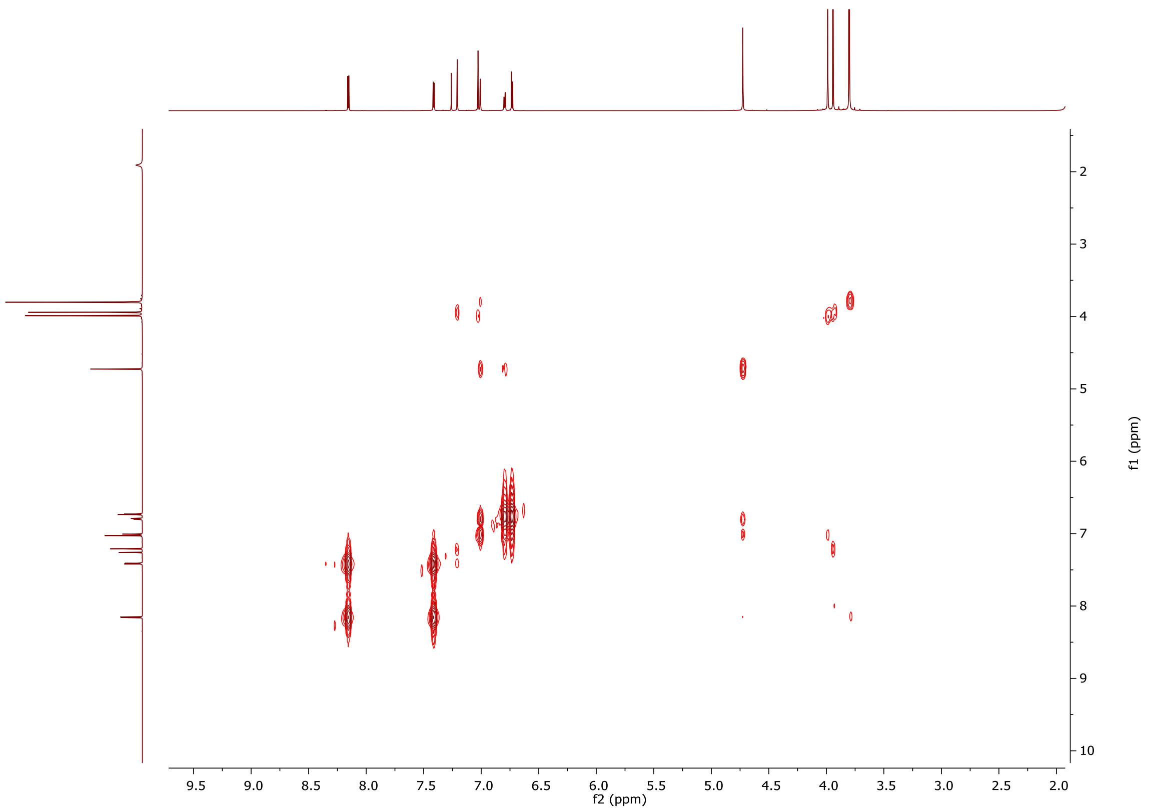

S64: HSQC NMR Spectrum for Compound **12** in $CDCl_3$

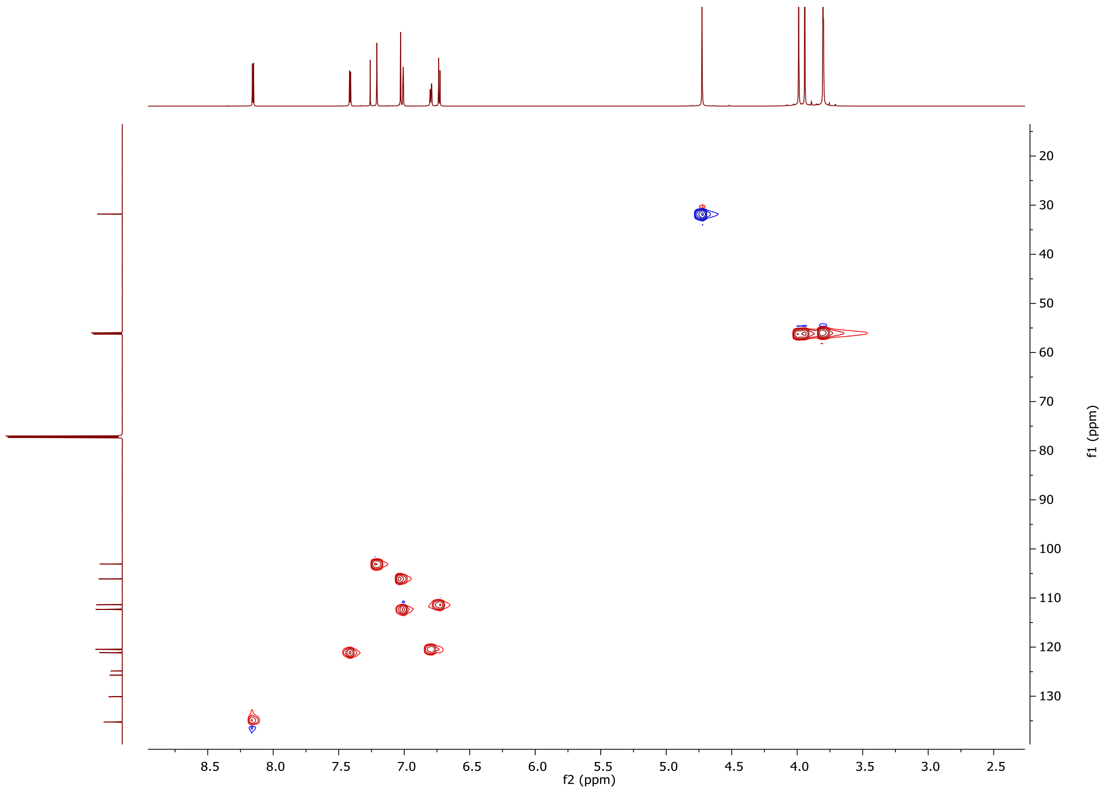

S65: HMBC NMR Spectrum for Compound **12** in $CDCl_3$

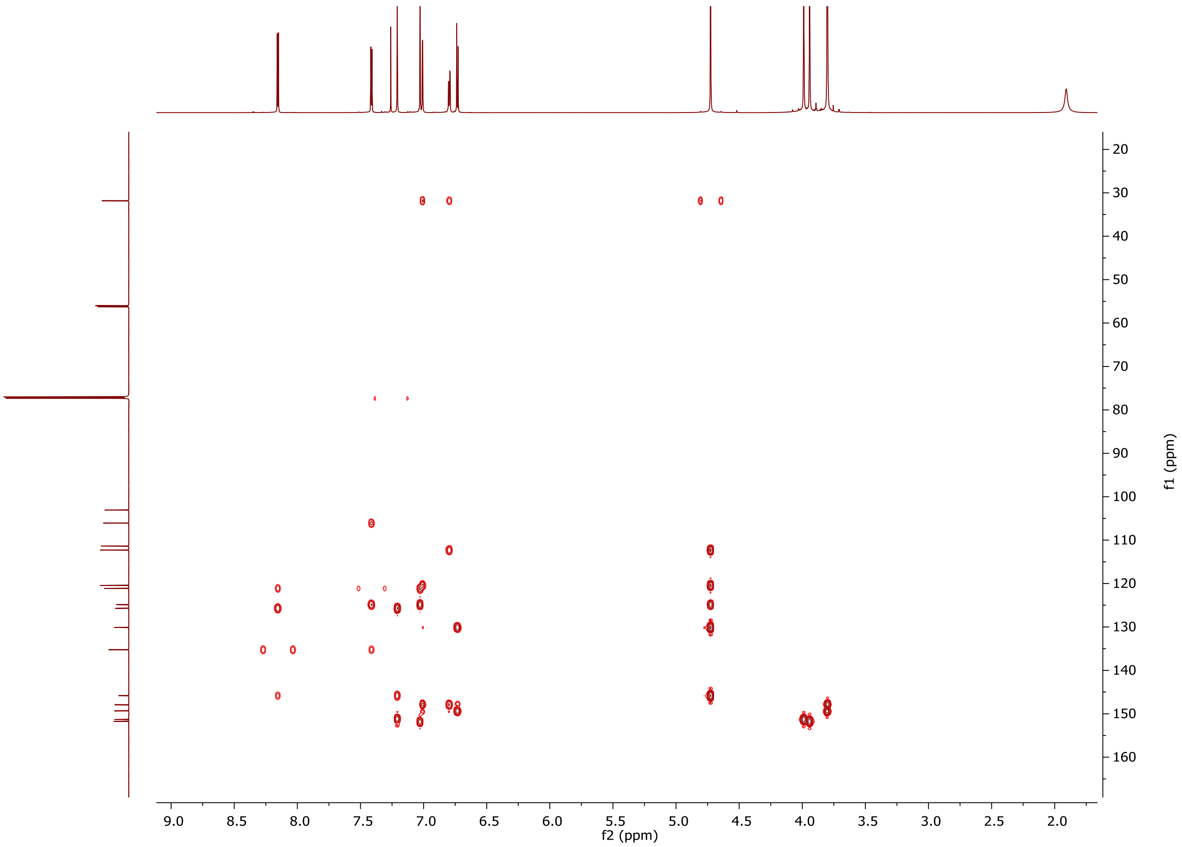

S66: ^{1}H NMR Spectrum for Compound **12** in CD_3OD

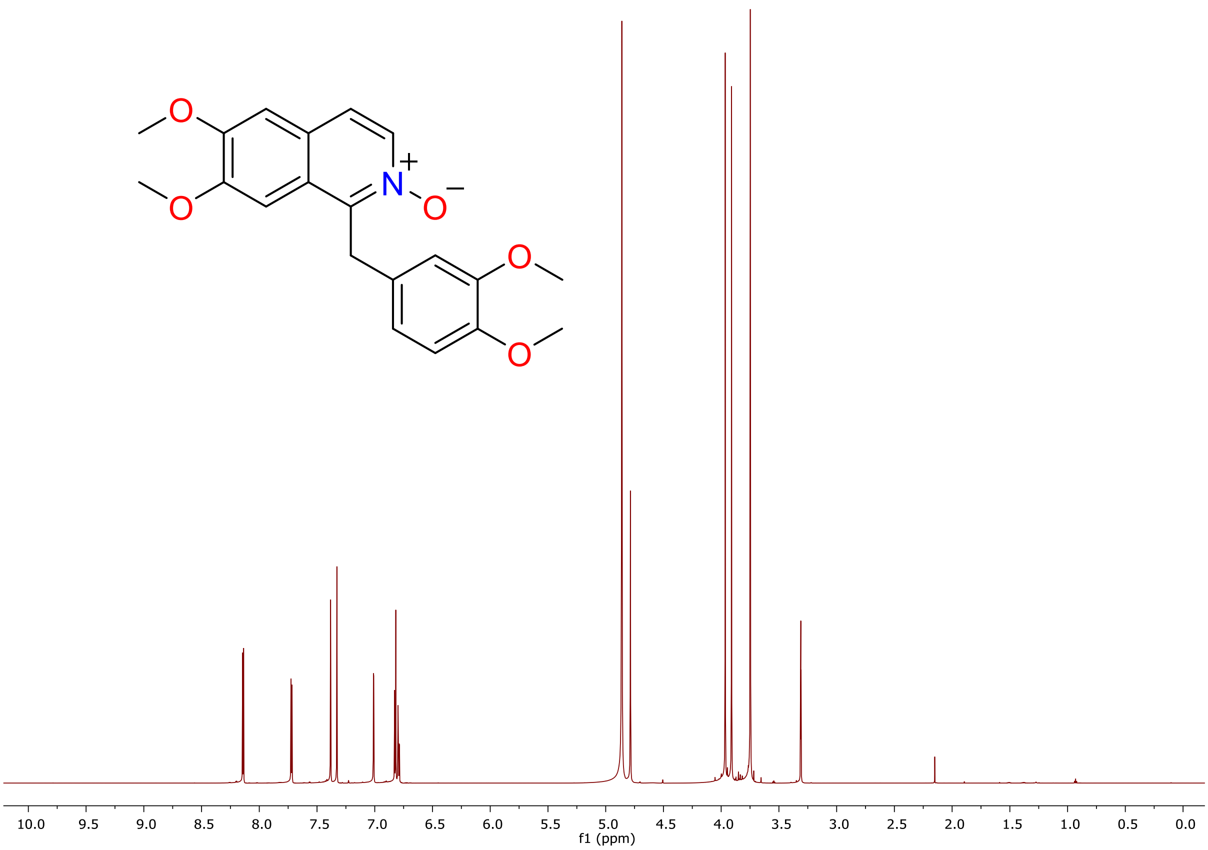

S67: ^{13}C NMR Spectrum for Compound **12** in CD_3OD

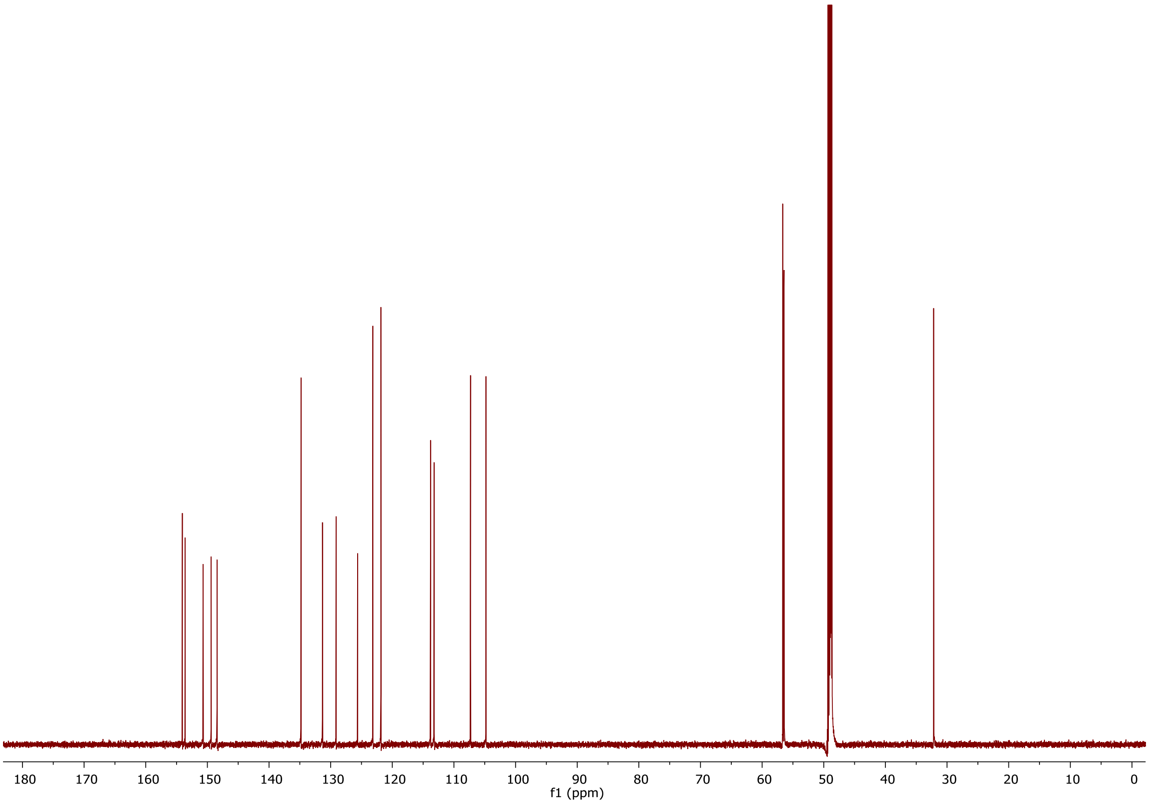

S68: COSY NMR Spectrum for Compound **12** in CD_3OD

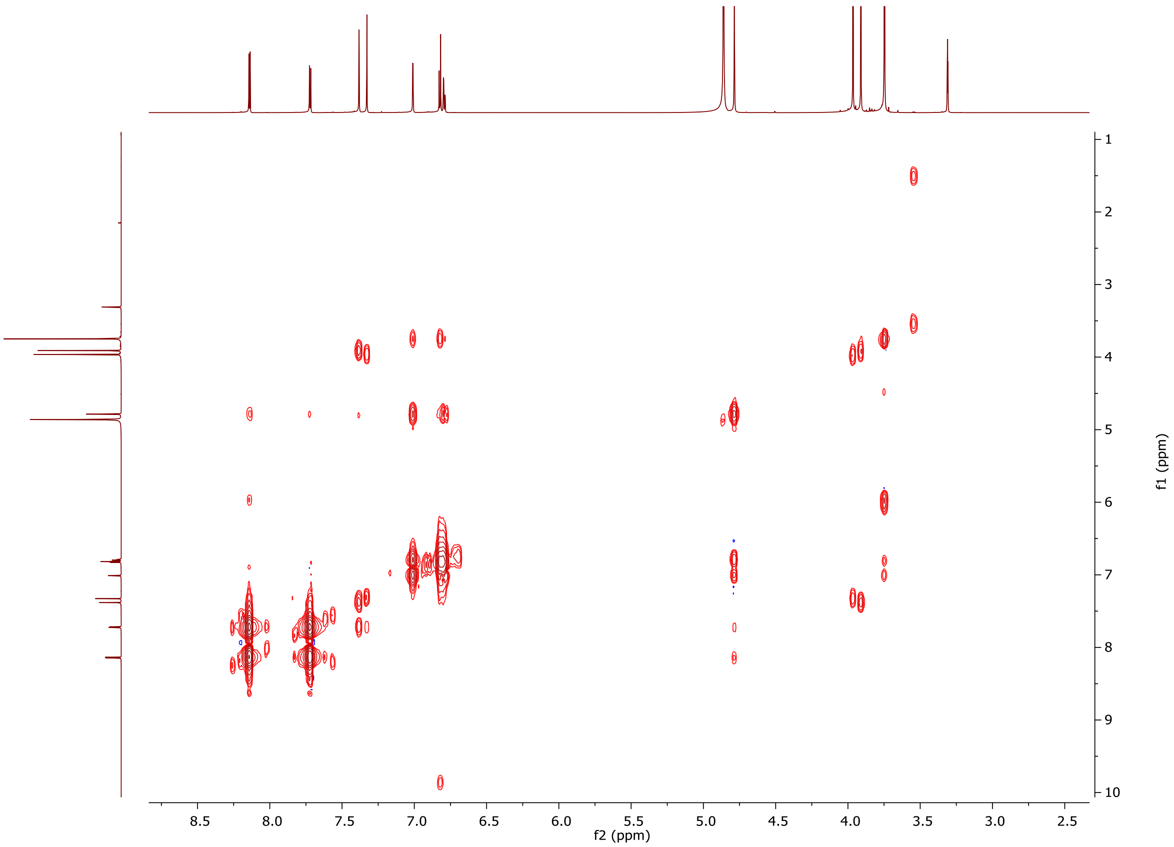

S69: HSQC NMR Spectrum for Compound **12** in CD_3OD

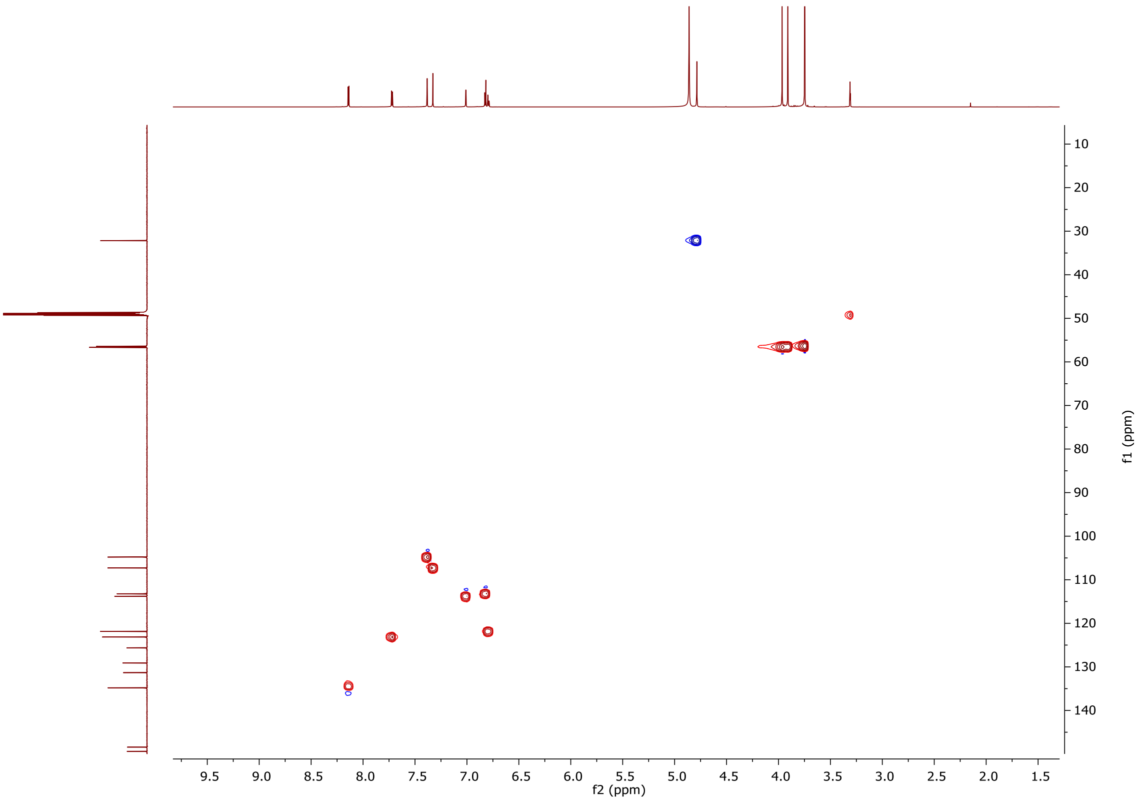

S70: HMBC NMR Spectrum for Compound **12** in CD_3OD

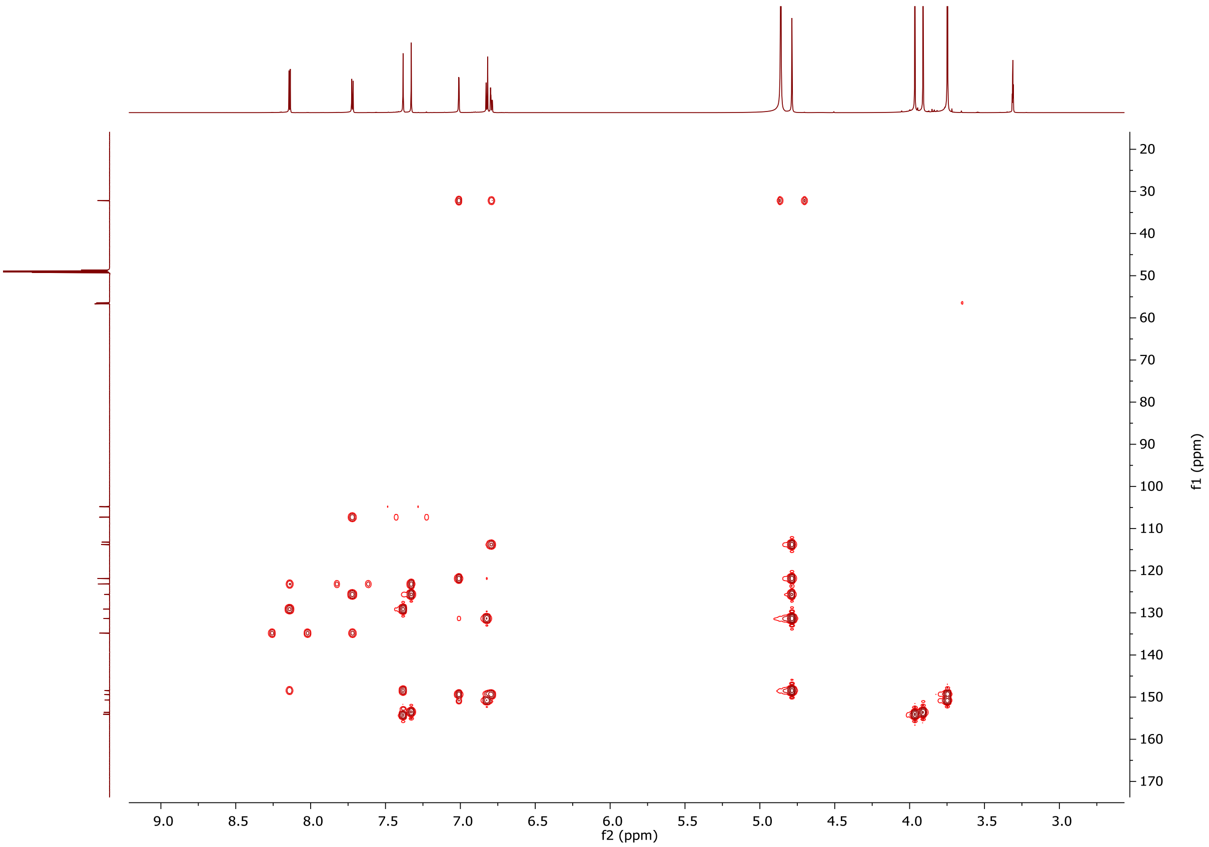

S71: Diversinate optimisation reactions with papaverine HCl (**1**) and the free-base of papaverine (**1b**)

Cmpd	Solvent	Eq. diversinate	Eq. TBHP	Time	% yield of **2**	% of recovered starting material
**1a**	CH_2Cl_2/H_2O	3	3	16 h	15%	8%
**1a**	CH_2Cl_2/H_2O	6	6	16 h	24%	7%
**1a**	$DMSO/H_2O$	3	3	16 h	10%	5%
**1a**	$DMSO/H_2O$	6	6	16 h	11%	8%
**1b**	CH_2Cl_2/H_2O	6	6	16 h	15%	5%
**1b**	$DMSO/H_2O$	6	6	16 h	12%	7%
**1b**	$CH_2Cl_2/TFA/H_2O$	6	6	16 h	10%	9%
**1b**	$DMSO/TFA/H_2O$	6	6	16 h	9%	8%

S72: Stacked ^1H NMR (800 MHz) spectra of the HCl salt (top) and free base (bottom) of papaverine in CD_3OD.

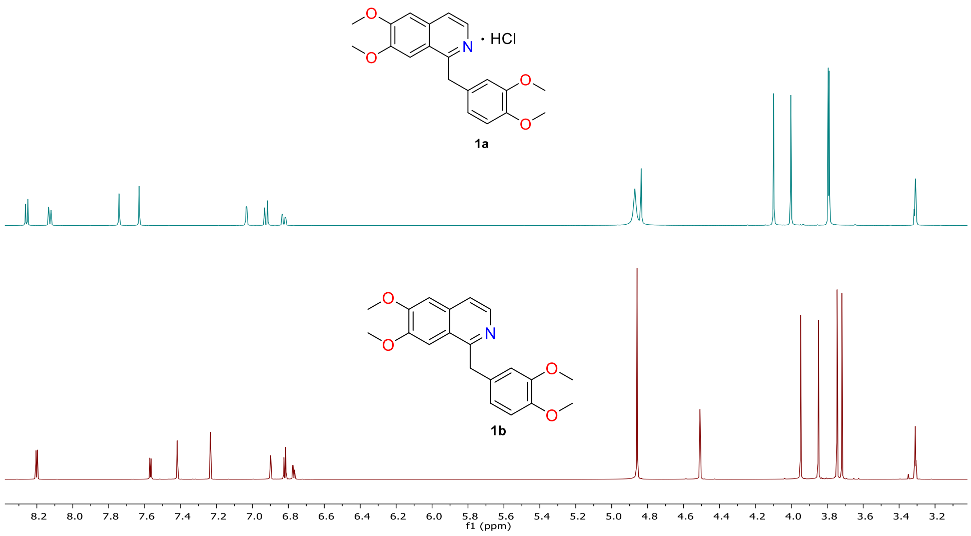

S73: ^{1}H (800 MHz) and ^{13}C (200 MHz) NMR data for papaverine HCl (**1a**) and the free base of papaverine (**1b**) in CD_3OD at 25 °C.

Position	HCl salt of papaverine (**1a**)		Free base of papaverine (**1b**)	
	$\delta_{C,}$ type	δ_H, mult. (*J* in Hz)	$\delta_{C,}$ type	δ_H, mult. (*J* in Hz)
1	155.8, C		159.1, C	
2				
3	130.4, CH	8.26, d, *J* = 6.5 Hz	140.5, CH	8.22, d, *J* = 5.8 Hz
4	123.3, CH	8.13, d, *J* = 6.5 Hz	120.5, CH	7.59, d, *J* = 5.8 Hz
4a	138.9, C		135.4, C	
5	107.5, CH	7.63, s	106.5, CH	7.25, s
6	159.2, C		154.6, C	
7	154.5, C		151.7, C	
8	106.2, CH	7.74, s	105.6, CH	7.44, s
8a	123.9, C		124.3, C	
9	37.8, CH_2	4.84, s	42.0, CH_2	4.53, s
6-OMe	57.4, CH_3	4.10, s	56.48, CH_3	3.97, s
7-OMe	57.1, CH_3	4.00, s	56.45, CH_3	3.87, s
12-OMe	56.5*, CH_3	3.79, s	56.36, CH_3	3.74, s
13-OMe	56.6*, CH_3	3.79, s	56.39, CH_3	3.77, s
10	129.1, C		133.6, C	
11	113.9, CH	7.03, d, *J* = 2.1 Hz	113.6, CH	6.92, d, *J* = 2.0 Hz
12	151.1, C		150.6, C	
13	150.3, C		149.1, C	
14	113.5, CH	6.92, d, *J* = 8.3 Hz	113.2, CH	6.84, d, *J* = 8.3 Hz
15	122.4, CH	6.83, dd, *J* = 8.3, 2.1 Hz	121.9, CH	6.79, dd, *J* = 8.3, 2.0 Hz

*interchangeable signals.

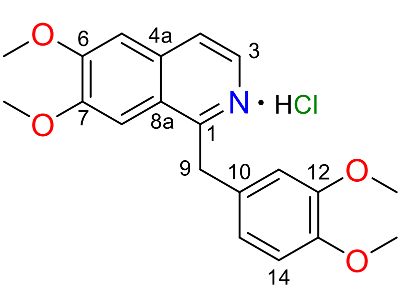

1a

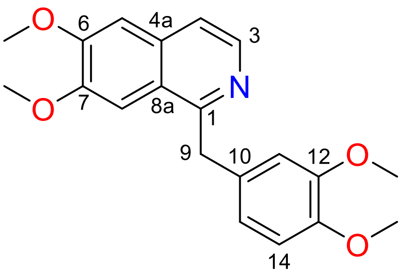

1b

S74: Expansion of ^{13}C NMR spectrum of compound **2** showing the quartet of the CF_3 group.

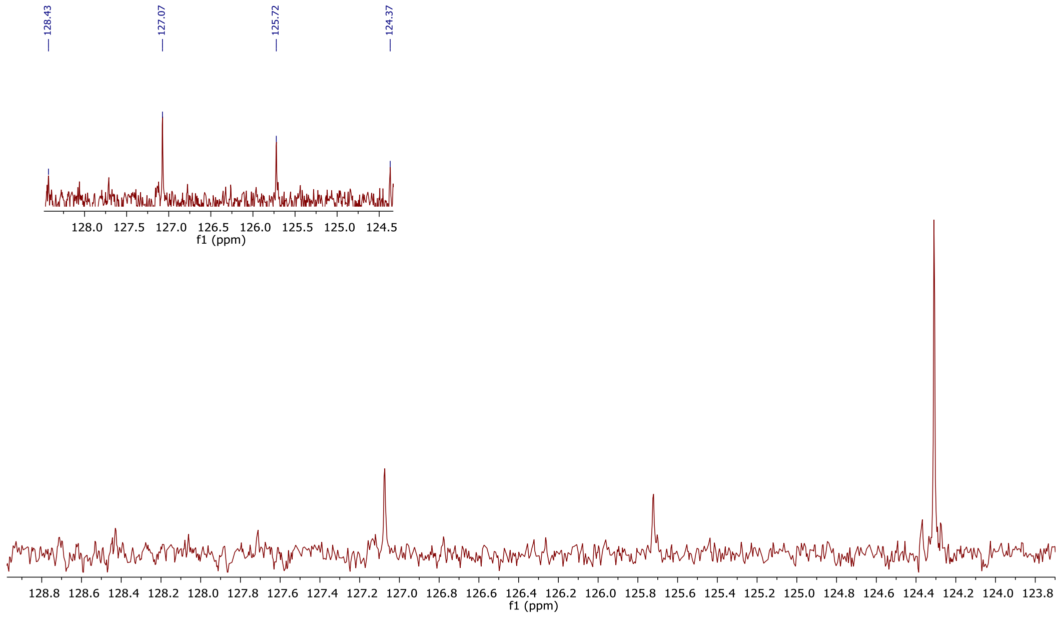

S75: Expansion of HMBC spectrum of compound **3**.

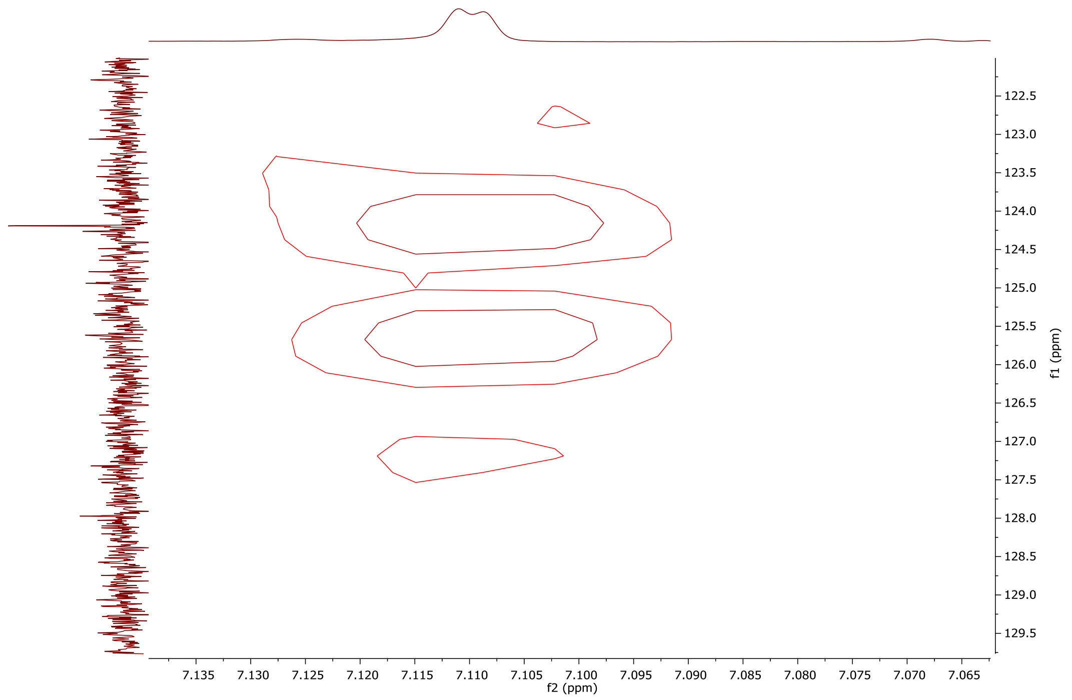

Supplement: Supplementary file 1 [file molecules-24-03938-s001.pdf]
